# Designer Solid Self‐Emulsifying Nanovaccines Enable Dual Modulation of Dendritic Cells and T Cells for Potent Antitumor Immunity

**DOI:** 10.1002/advs.202512139

**Published:** 2025-11-07

**Authors:** Xueying Shen, Shiqi Fan, Jia He, Lanqing Luo, Junyao Li, Chengcheng Wu, Kairu Yang, Xiaojun Xia, Rui Kuai

**Affiliations:** ^1^ School of Pharmaceutical Sciences Tsinghua University Beijing 100084 China; ^2^ Tsinghua‐Peking Center for Life Sciences Beijing 100084 China; ^3^ State Key Laboratory of Oncology in South China Guangdong Provincial Clinical Research Center for Cancer Sun Yat‐sen University Cancer Center Guangzhou 510060 China; ^4^ Hainan Academy of Medical Sciences Hainan Medical University Haikou 571199 China

**Keywords:** lipid raft, protein corona, self‐emulsifying nanovaccine

## Abstract

Peptide‐based cancer vaccines offer favorable safety and stability profiles but are limited by rapid clearance and poor immunogenicity. Here, a ≈20 nm solid self‐emulsifying (SSE) nanovaccine platform that co‐delivers peptide antigens and an oligonucleotide adjuvant containing the 5'‐C‐phosphate‐G‐3' (CpG) motif is reported. This formulation elicits T‐cell responses 40 fold higher than those of conventional emulsified vaccines and even achieves complete tumor regression at low doses. Apolipoprotein E (ApoE) adsorbed on SSE vaccines enhances lymph node targeting and dendritic cell internalization. Furthermore, SSE is internalized by T cells and promotes lipid raft formation, thereby further sensitizing T cells to activation. These findings reveal a dual mechanism of immune regulation through the simultaneous engagement of dendritic cells and T cells. The SSE platform offers a clinically translatable strategy for potent cancer immunotherapy and provides mechanistic insights into nanoparticle–immune cell interactions that may guide the design of next‐generation nanovaccines.

## Graphical abstract

A solid self‐emulsifying (SSE) platform is loaded with peptide antigens and CpG to form the SSE vaccine. An ApoE‐enriched corona promotes dendritic cell internalization, enabling antigen presentation and T cell activation. Squalene from the SSE also sensitizes T cells by promoting lipid raft formation. This elicits robust T cell immunity, leading to tumor regression.

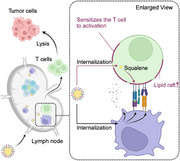

## 1 Introduction

The identification of neoantigens has transformed cancer vaccine development by enabling precise activation of tumor‐specific T cells.^[^ ^1^, ^2^, ^3^ ^]^ Among various platforms, peptide‐based vaccines have attracted interest due to their favorable safety, stability, and manufacturing profiles.^[^ ^4^, ^5^, ^6^, ^7^, ^8^ ^]^ However, their clinical efficacy remains limited by poor lymph node accumulation and rapid clearance following subcutaneous or intramuscular injection,^[^ ^9^ ^]^ which restricts their ability to elicit robust cytotoxic T‐cell responses.

To overcome these barriers, delivery systems that enhance vaccine trafficking to lymphoid tissues and promote antigen presentation by dendritic cells (DCs) are critically needed. Conventional emulsions, such as Montanide, form depot structures that reduce antigen clearance^[^ ^10^, ^11^ ^]^ but can also sequester T cells at the injection site, leading to apoptosis and immune suppression.^[^ ^12^, ^13^ ^]^ Similarly, AS03 emulsions have an average size of 150–200 nm, thus allowing for prolonged retention at the injection site and recruitment of immune cells that migrate to draining lymph nodes to elicit immune responses.^[^ ^14^, ^15^ ^]^ In contrast, ≈20‐nm nanoparticles can directly enter lymphatic vessels, reaching lymph nodes at a higher concentration than 200‐nm nanoparticles.^[^ ^16^, ^17^ ^]^ More recent strategies, including ultrasmall nanodiscs (≈10 nm) and albumin‐binding amphiphilic constructs, have improved lymph node targeting and enabled peptide vaccines to access lymph node‐resident DCs, which process and present antigens to activate T cells. Although these platforms are promising, the surface properties of nanodiscs or albumin are fixed, making it difficult to further tune the delivery profile.^[^ ^18^, ^19^ ^]^ Furthermore, the mechanisms governing nanoparticle trafficking and whether nanoparticles functionally modulate both DCs and T cells remain incompletely understood.

**Figure 1:**
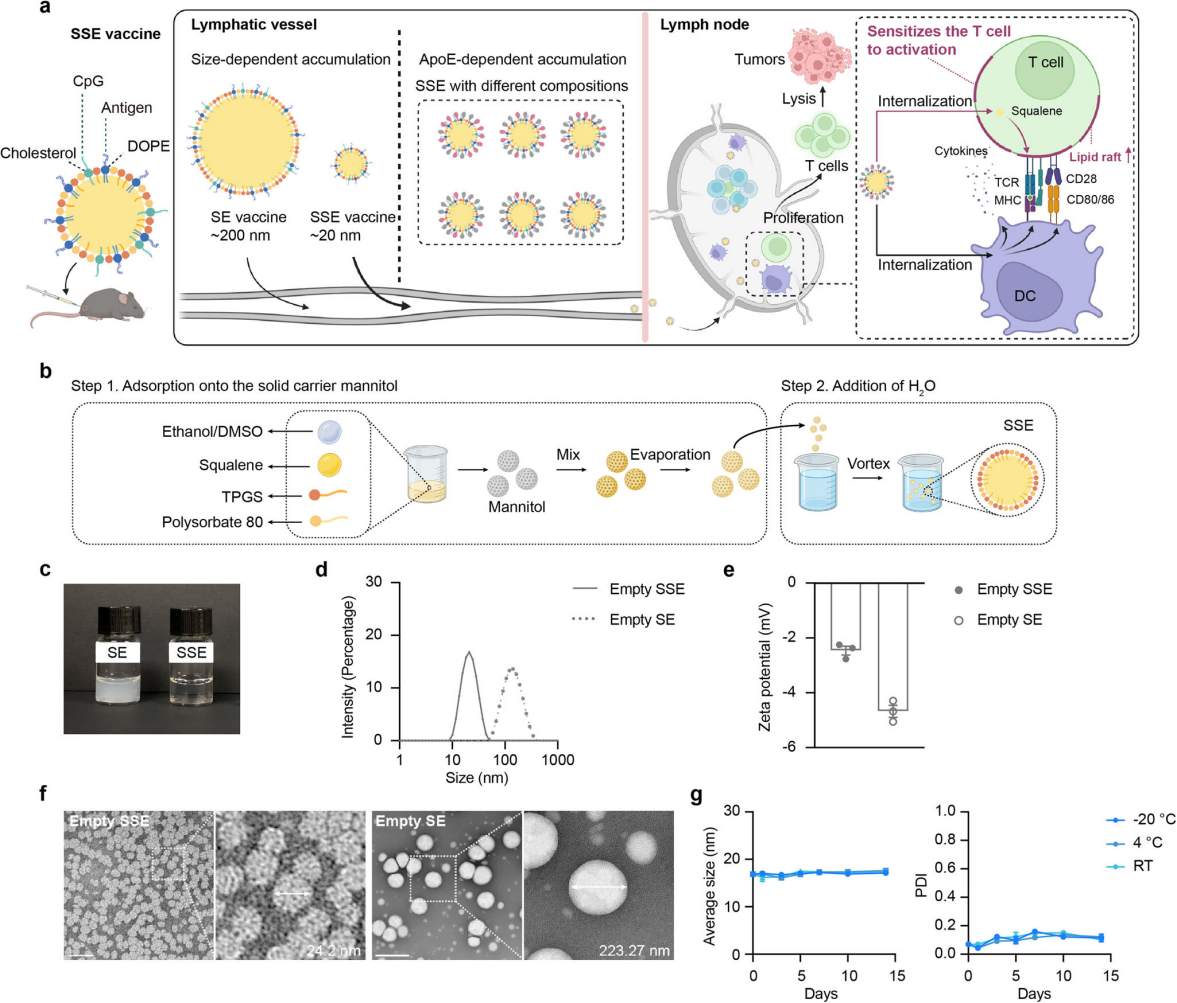
Preparation and characterization of SSE. a) Schematic illustration of the SSE vaccine for cancer immunotherapy. b) The schematic shows the preparation process of SSE. c) The picture of empty SE and SSE. d) The size distribution of SE and SSE. The experiment was performed three times with similar results. e) The zeta potentials of SE and SSE (n = 3 experimental replicates per group). f) The representative TEM images of SE and SSE. The scale bar is 50 nm for SSE and 500 nm for SE. g) The size distribution of SSE after storage under different conditions (n = 3 experimental replicates per group).

Here, we present a solid self‐emulsifying (SSE) nanovaccine platform (≈20 nm) composed of tocofersolan (TPGS), polysorbate 80 (PS80), and squalene. Through systematic formulation optimization, we identify an SSE system that efficiently accumulates in draining lymph nodes. When loaded with peptide antigens and a Toll‐like receptor‐9 (TLR9) agonist (CpG),^[^ ^20^ ^]^ this SSE formulation induces potent antitumor T‐cell immunity. Mechanistic studies reveal that the ultrasmall particle size and an ApoE‐enriched protein corona are critical for efficient DC targeting in lymph nodes. Unexpectedly, SSE nanoparticles are also internalized by T cells, promoting lipid raft formation and enhancing their activation by DCs via a squalene/squalene epoxidase (SQLE)‐dependent pathway (**Figure** **1**a). These findings establish SSE vaccines as a potent and clinically relevant platform for cancer immunotherapy and provide mechanistic insights into the dual regulation of DCs and T cells, offering a blueprint for the design of next‐generation nanovaccines.

## 2 Results

### 2.1 Preparation and Characterization of Solid Self‐Emulsifying Nanoparticles

We first established a robust protocol for preparing solid self‐emulsifying nanoparticles (SSE) (Figure 1b). Briefly, TPGS, PS80, and squalene were dissolved in ethanol, mixed with the solid carrier mannitol, and then dried at 40 °C to remove the solvent (Figure S1a, Supporting Information). Upon hydration with water, this solid mixture formed homogeneous SSE nanoparticles with a diameter of ≈20 nm. In contrast, omitting mannitol during preparation resulted in the formation of larger nanoparticles (≈200 nm), denoted as self‐emulsifying nanoparticles (SE), indicating that mannitol plays a crucial role in facilitating the formation of ultrasmall SSE nanoparticles (Figure 1c,d; Table S1, Supporting Information). Both SSE and SE exhibited near‐neutral zeta potentials, consistent with their non‐ionic chemical structures (Figure 1e). Transmission electron microscopy (TEM) analysis further confirmed SSE's homogeneous ultrasmall size, which was significantly smaller than SE (Figure 1f). We found that when the ratio of total surfactants (TPGS plus PS80) to squalene was maintained above 3:1, SSE retained a size of ≈20 nm. However, when this ratio dropped below 3:1, the SSE size exceeded 100 nm (Figure S1b,c, Supporting Information). Notably, the SSE size remained ≈20 nm even when the TPGS/PS80/squalene ratio varied from 1:6:1 to 6:1:1 (Figure S1d,e, Supporting Information), highlighting the tunability of SSE's surface properties for modulating delivery profiles and immune responses. Moreover, introducing other solvents, such as dimethyl sulfoxide (DMSO), or altering the mannitol‐to‐solvent ratio had no significant impact on particle size, demonstrating the robustness of the preparation process (Figure S1f–h, Supporting Information). SSE also exhibited excellent stability, maintaining its size after 14 days of storage at room temperature, 4, or −20 °C (Figure 1g). In contrast, SE exhibited noticeable aggregation and an increase in particle size after 14 days at 4 °C (Figure S1i,j, Supporting Information).

### 2.2 Efficient Accumulation of SSE in the Draining Lymph Nodes

**Figure 2:**
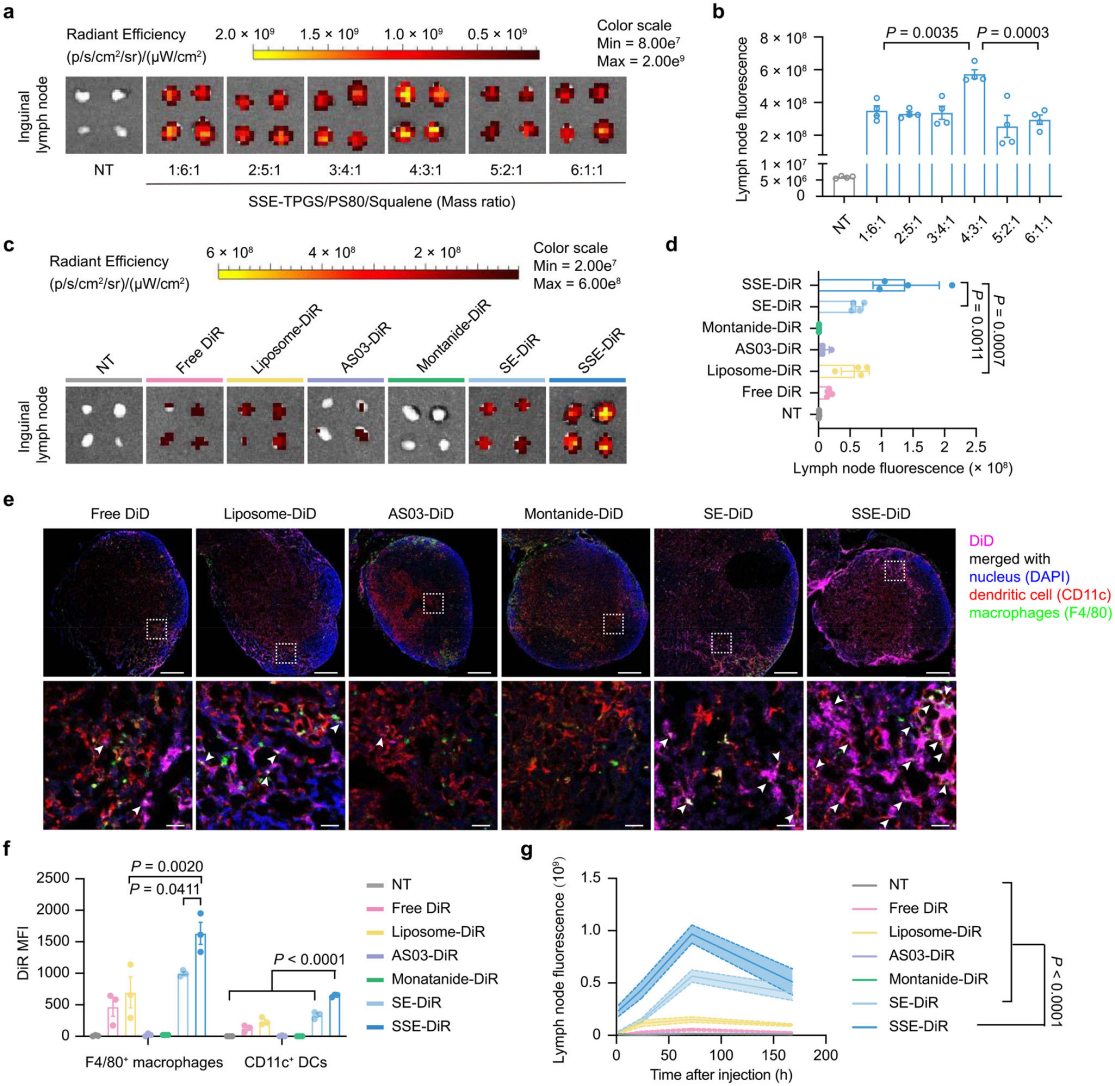
SSE accumulates in the draining lymph nodes in a composition‐dependent manner. a) The accumulation of various formulations of SSE in inguinal lymph nodes 24 h after subcutaneous injection (n = 4 experimental replicates per group). b) Quantitative results of average fluorescence intensity in (a) (n = 4 experimental replicates per group). c) The accumulation of various formulations in inguinal lymph nodes 24 h after subcutaneous injection (n = 4). d) Quantitative results of average fluorescence intensity in (c) (n = 4 experimental replicates per group). e) Immunofluorescence of inguinal lymph nodes 24 h after subcutaneous injection of indicated formulations (DiD, purple; F4/80, green; CD11c, red; nucleus, blue). Scale bars represent 500 µm (overview) and 50 µm (inset). f) The internalization of various formulations by F4/80^+^ macrophages and CD11c^+^ DCs in the inguinal lymph nodes 24 h after subcutaneous injection (n = 3 experimental replicates per group). g) Accumulation of various formulations in inguinal lymph nodes at different time points (n = 4 experimental replicates per group). Data represent mean ± SEM. Data were analyzed by one‐way ANOVA with Tukey's multiple comparisons test (b,d,f) or two‐way ANOVA with Dunnett's multiple comparisons test (g).

Having generated a library of ultrasmall SSE nanoparticles with varying ratios of TPGS, PS80, and squalene, we next investigated their accumulation in draining lymph nodes, the primary sites of T‐cell activation. We subcutaneously injected DiR‐labeled SSE into C57BL/6 mice and imaged the harvested lymph nodes 24 h post‐injection. SSE accumulation in the lymph nodes was composition‐dependent, with the formulation containing TPGS, PS80, and squalene at a 4:3:1 ratio exhibiting the highest accumulation. In contrast, SSE with excessively high ratios of either TPGS or PS80 showed reduced lymph node accumulation (**Figure** **2**a,b). Based on these findings, we selected SSE (4:3:1) for further studies.

To benchmark SSE's lymph node drainage efficiency, we compared it to other commonly used peptide vaccine delivery systems, such as Montanide, AS03, and liposomes (Figure 2c,d; Figure S2a–c, Supporting Information). Notably, SSE exhibited 181.9, 13.9, and 2.4 fold greater lymph node accumulation than Montanide, AS03, and liposomes, respectively. Moreover, SSE accumulated two times more in the lymph nodes than SE with the same composition, highlighting the critical role of particle size in enhancing lymphatic delivery. Immunofluorescence imaging of draining lymph nodes revealed substantial colocalization of SSE‐DiD with antigen‐presenting cells (APCs), such as DCs and macrophages (Figure 2e). In contrast, only minimal amounts of other formulations were associated with APCs. Flow cytometry analysis further confirmed that SSE was taken up more efficiently by CD11c^+^ DCs and F4/80^+^ macrophages in inguinal lymph nodes compared to other formulations (Figure 2f). Interestingly, we also observed SSE internalization by T and B cells, although at lower levels than APCs (Figure S2d,e, Supporting Information). Additionally, SSE accumulated more efficiently in inguinal lymph nodes and persisted longer than other formulations (Figure 2g; Figure S3, Supporting Information). Collectively, these results demonstrate that the uniform 20 nm SSE exhibits robust lymph node accumulation, making it a promising vaccine delivery platform.

### 2.3 SSE Vaccine Exhibits Efficient Antigen Internalization and APC Activation

**Figure 3:**
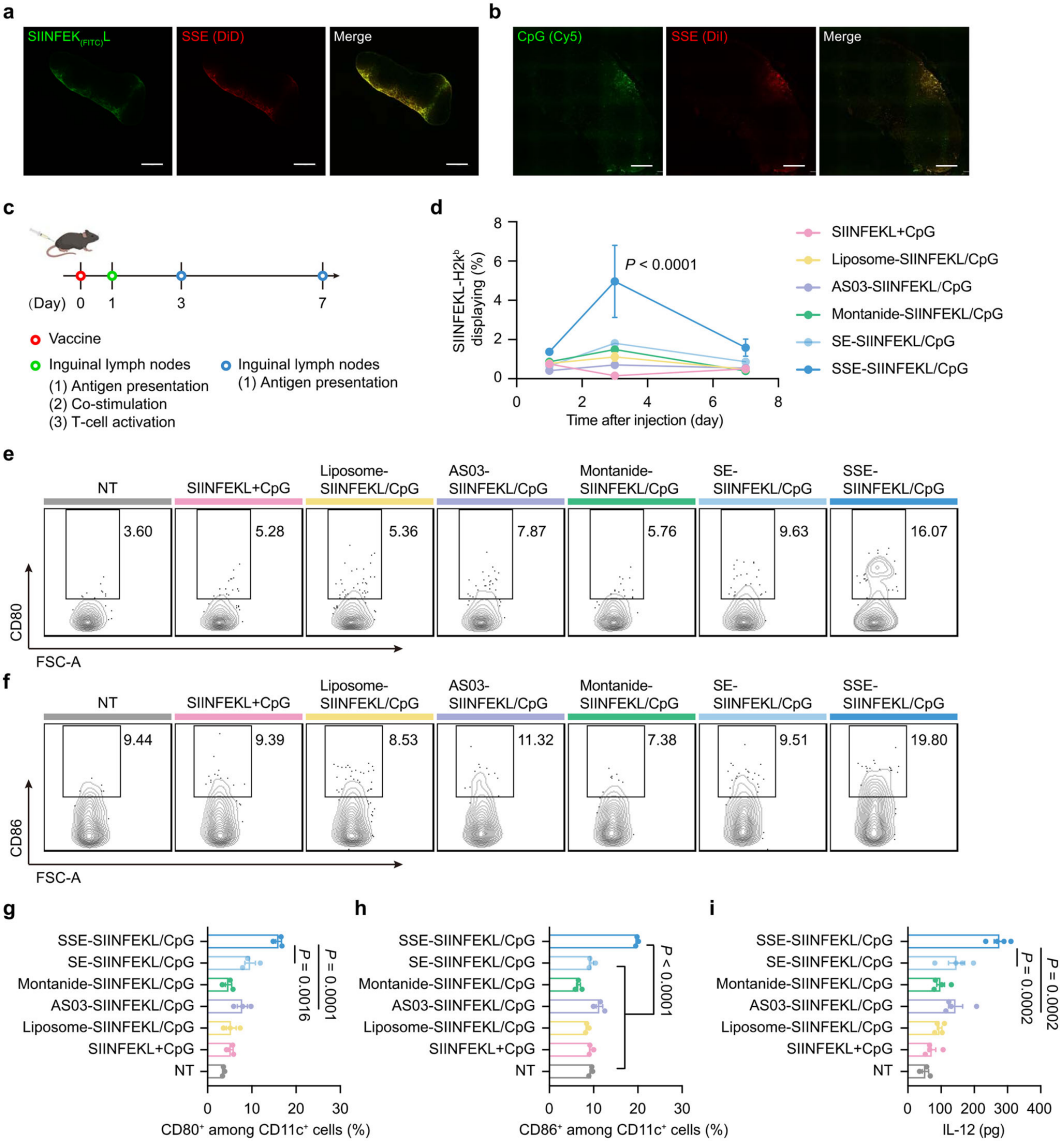
The SSE vaccine efficiently activates DC and induces strong antigen presentation. a,b) C57BL/6 mice were subcutaneously injected with the SSE vaccine containing SIINFEK_(FITC)_L and CpG‐Cy5. Shown are (a) the colocalization of SIINFEK_(FITC)_L and SSE, and (b) the colocalization of CpG‐Cy5 with SSE in the inguinal lymph nodes 24 h after subcutaneous injection. Scale bars represent 500 µm. c) Strategy of APC activation analyses. d–i) C57BL/6 mice were subcutaneously injected with different vaccines containing SIINFEKL and CpG. Shown are (d) antigen presentation profiles on DCs at different time points post‐vaccination. e,f) Representative flow cytometry scatter plots showing maturation markers CD80 and CD86 on DCs 24 h after injection of various formulations (n = 3 experimental replicates per group). g,h) Quantification of maturation markers CD80 (e) and CD86 (f) (n = 3 experimental replicates per group). i) IL‐12 production in inguinal lymph nodes 24 h after injection (n = 3 or 4 experimental replicates per group). Data represent mean ± SEM. Data were analyzed by two‐way ANOVA with Dunnett's multiple comparisons test (d) or one‐way ANOVA with Tukey's multiple comparisons test (g–i).

After confirming the efficient lymph node drainage of SSE, we explored its potential as a vaccine delivery platform. To this end, we loaded SSE with lipid‐modified antigen peptides and cholesterol‐modified CpG to formulate the SSE vaccine (Figure S4a, Supporting Information). Loading antigen peptides and CpG did not significantly alter SSE's size or structure (Figure S4b–d and Table S1, Supporting Information). However, the zeta potential decreased further due to the negative charge of CpG (Figure S4e, Supporting Information). The encapsulation efficiencies of lipid‐modified antigen and cholesterol‐modified CpG were ≈60% and 20%, respectively (Figure S4f,g, and Table S2, Supporting Information). Importantly, the SSE vaccine was amenable to freeze‐drying and could be readily reconstituted into homogeneous 20 nm nanoparticles with comparable encapsulation efficiencies (Figure S4h and Table S2, Supporting Information), demonstrating superior physicochemical stability and offering clear advantages for long‐term storage, cold‐chain independence, and ease of distribution in diverse settings. Next, we assessed the in vivo lymphatic delivery of antigens and adjuvants. To enable visualization, the model antigen SIINFEKL was fluorescently labeled with FITC on its lysine residue, denoted as SIINFEK_(FITC)_L, a modification previously reported to retain H‐2K^b^ binding.^[^ ^21^ ^]^ CpG was labeled with Cy5. At 24 h post‐subcutaneous injection, SIINFEK_(FITC)_L and Cy5‐labeled CpG colocalized with SSE in the lymph nodes (**Figure** **3**a,b), confirming that SSE successfully delivered both antigen peptides and CpG to lymphatic tissues. We then examined the cellular internalization of antigen peptides by BMDCs. Free SIINFEK_(FITC)_L and CpG exhibited weak BMDC internalization at all observed time points (2, 6, 24, and 48 h). In contrast, SSE‐SIINFEK_(FITC)_L/CpG was rapidly and extensively internalized as early as 2 h, with strong antigen signals persisting in BMDCs for over 48 h (Figure S5a,b, Supporting Information). Notably, by 48 h, some SIINFEK_(FITC)_L from SSE‐SIINFEK_(FITC)_L/CpG but not from SIINFEK_(FITC)_L+CpG was detected outside lysosomes and within the cytoplasm, suggesting successful antigen cross‐presentation. SSE vaccines also exhibited a favorable safety profile in bone marrow‐derived dendritic cells (BMDCs) (Figure S5c,d, Supporting Information).

We next analyzed antigen presentation and costimulatory molecules of APCs and T cell activation in inguinal lymph nodes (Figure 3c). Compared to other formulations, SSE‐SIINFEKL/CpG induced stronger and more sustained antigen presentation by DCs (Figure 3d; Figure S6, Supporting Information). Additionally, it upregulated CD80 and CD86 expression on DCs and macrophages to a greater extent than other formulations (Figure 3e–h; Figure S7, Supporting Information). SSE‐SIINFEKL/CpG also triggered the highest levels of IL‐12 and TNF‐α production in inguinal lymph nodes (Figure 3i; Figure S8a, Supporting Information). Notably, the level of IL‐12 produced by BMDCs treated with SSE‐CpG was comparable to that of free CpG in vitro, suggesting that the strong adjuvant effects in vivo are also dependent on the robust accumulation of SSE in the draining lymph nodes (Figure S8b, Supporting Information). Consistent with enhanced DC activation, it induced the highest levels of CD69^+^ T cells in inguinal lymph nodes compared to control formulations (Figure S9, Supporting Information). Collectively, these findings demonstrate that the SSE vaccine facilitates efficient antigen internalization, promotes DC activation, and enhances antigen presentation, highlighting its potential as a potent vaccine delivery system.

### 2.4 SSE Vaccine Induces Potent T‐Cell Responses

**Figure 4:**
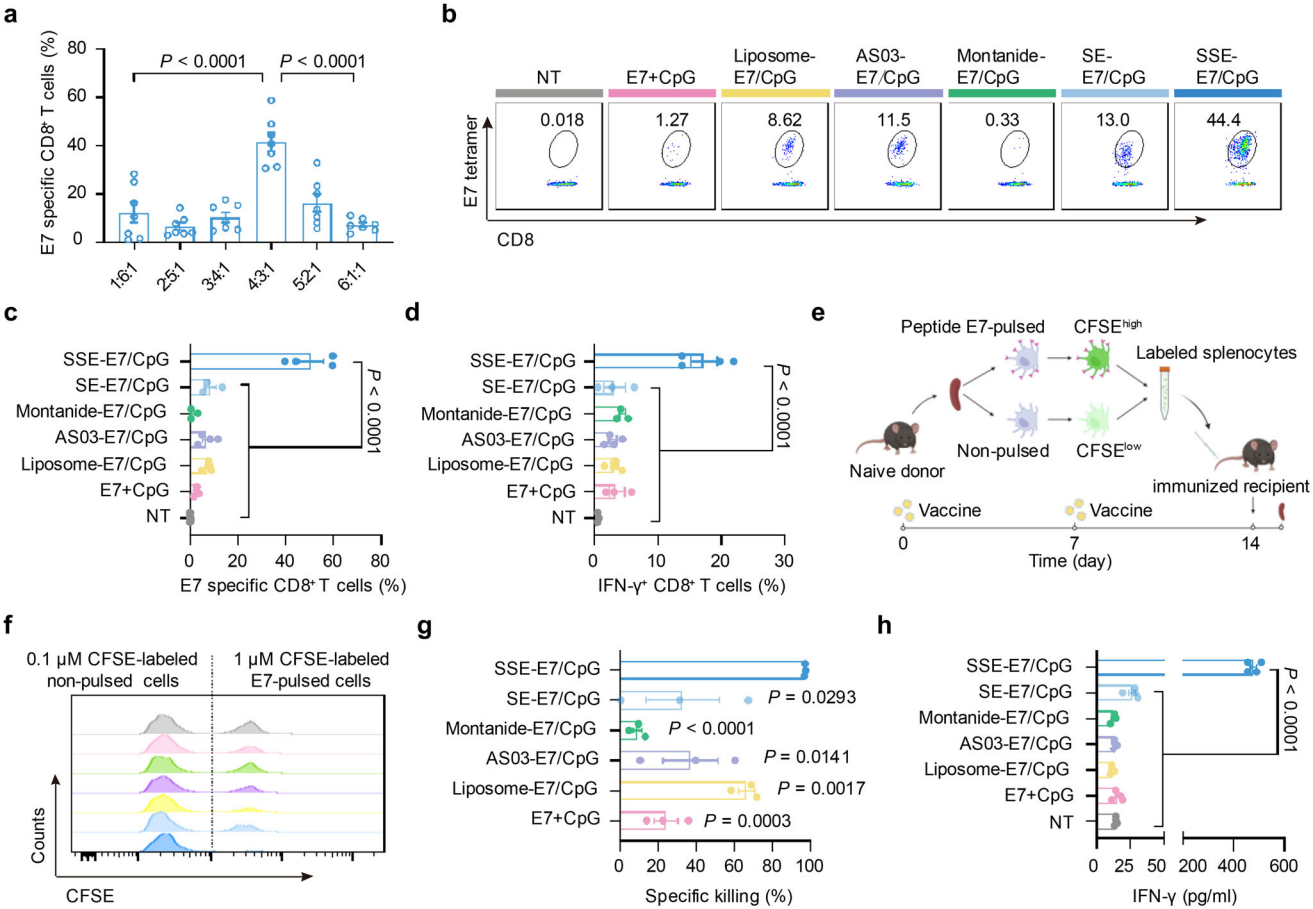
SSE vaccine induces potent T‐cell responses. Mice were immunized with vaccines containing 3 µg E7 and 1 µg CpG on days 0 and 7. The immune responses were analyzed on day 14. a) Quantitative analyses of antigen‐specific CD8^+^ T cells among PBMCs on day 14 after immunization with SSE with different compositions (n = 7 mice per group). The numbers along the *x*‐axis represent the ratios of TPGS, PS80, and squalene. b,c) Representative flow cytometry scatter plots and quantitative analyses of antigen‐specific CD8^+^ T cells on day 14 after immunization with indicated formulations. (n = 3 or 4 mice per group). d) The quantification of IFN‐γ‐producing CD8^+^ T cells on day 14. (n = 3 or 4 mice per group). e) The schematic of the in vivo cytotoxicity assay. f,g) Representative flow cytometry histograms and quantitative analyses of E7‐specific cytotoxicity in vivo. (n = 3 mice per group). h) ELISA analysis of IFN‐γ produced by splenocytes after ex vivo restimulation with E7 on day 14 (n = 4 experimental replicates per group). Data represent mean ± SEM. Data were analyzed by one‐way ANOVA with Tukey's multiple comparisons test (a,c,d,g,h).

To assess the impact of lymph node targeting on immune responses, we immunized mice with two doses of SSE vaccines containing 3 µg per dose of E7_49_₋_57_ (an HPV‐16 epitope)^[^ ^22^ ^]^ and 1 µg per dose of CpG (Figure S10, Supporting Information). Consistent with its efficient lymph node accumulation, SSE‐E7/CpG (TPGS/PS80/squalene = 4:3:1) elicited robust antigen‐specific CD8^+^ T cell responses (**Figure** **4**a; Figure S11, Supporting Information). Due to its superior potency, SSE‐E7/CpG (4:3:1) was selected for further evaluation.

We next compared SSE‐E7/CpG (4:3:1) with other widely used peptide vaccine formulations. Remarkably, SSE‐E7/CpG induced antigen‐specific T‐cell responses that were 19.7, 42.6, 7.3, and 6.9 fold stronger than those elicited by soluble vaccines, Montanide, AS03, and liposomes, respectively (Figure 4b,c). In contrast, SE‐E7/CpG triggered weaker T‐cell responses, highlighting the critical role of SSE's ultrasmall particle size in driving potent T‐cell immunity. Notably, at 1.5 µg per dose of E7 and 0.5 µg per dose of CpG, SSE‐E7/CpG still elicited ≈10% antigen‐specific CD8^+^ T‐cell responses, underscoring the high potency of the SSE vaccine (Figure S12, Supporting Information). To further characterize the function of activated CD8^+^ T cells, we assessed cytokine production and cytotoxic activity in vivo. SSE‐E7/CpG significantly enhanced IFN‐γ production compared to control formulations (Figure 4d) and exhibited superior cytolytic activity (Figure 4e–g). Enhanced antigen‐specific immune responses were observed not only in peripheral blood but also in the spleen (Figure 4h; Figure S13, Supporting Information). To determine whether SSE itself possesses intrinsic immune‐stimulating properties, we immunized mice with SSE‐E7 and analyzed antigen‐specific CD8^+^ T‐cell responses. SSE‐E7 induced a modest immune response compared to the PBS control, suggesting SSE may independently enhance immune activation (Figure S14a,b, Supporting Information). This effect was not attributable to endotoxin contamination, as SSE‐E7 and PBS exhibited similarly low endotoxin levels (Figure S14c, Supporting Information). Collectively, these findings demonstrate that SSE vaccines elicit potent antigen‐specific CD8^+^ T‐cell responses, further underscoring the potential of SSE as an effective vaccine delivery platform.

### 2.5 SSE Vaccine Induces Potent T Cell Immunity in an ApoE‐Dependent Manner

**Figure 5:**
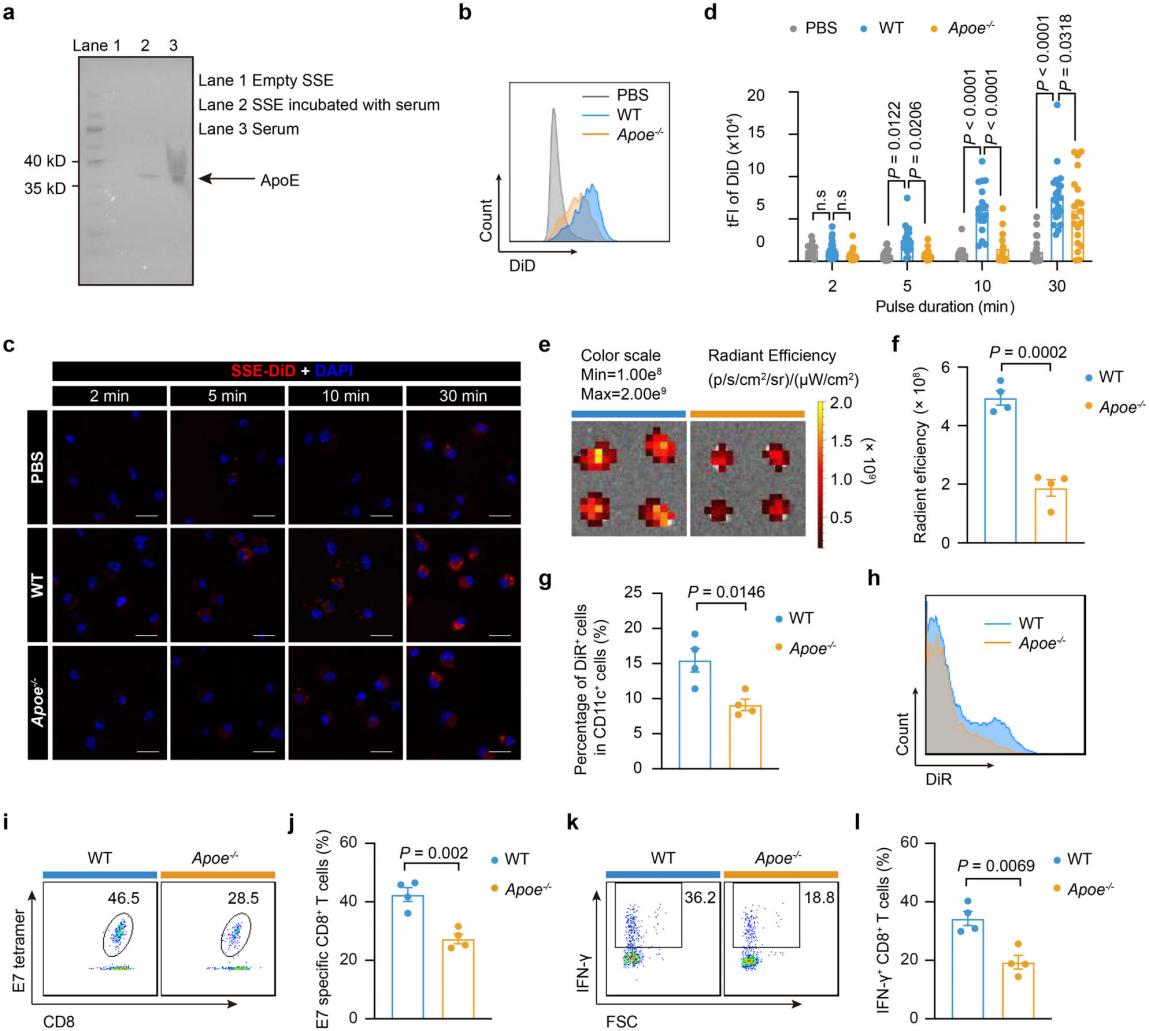
The ApoE‐containing protein corona of the SSE vaccine is critical for maintaining efficacy. a) Western blot confirms the presence of ApoE adsorbed on SSE. b–d) SSE‐DiD was pretreated with PBS, WT or *Apoe^−/−^* serum for 1 h, and then incubated with WT BMDCs for different lengths of time, and the cellular internalization of SSE‐DiD was analyzed by confocal microscopy or flow cytometry. Shown are (b) representative flow cytometry histograms of cellular internalization of SSE‐DiD by BMDCs at 30 min after incubation, c) confocal images of BMDCs after incubation with SSE‐DiD for indicated lengths of time (Scale bar, 20 µm), and d) the fluorescence quantification of cellular internalization of SSE‐DiD (pretreated under indicated conditions) in (c). (n = 20 cells). e–h) WT or *Apoe^−/−^* mice were subcutaneously injected with SSE‐DiR, and the lymph nodes were harvested at 24 h post‐injection for analysis. (n = 4 experimental replicates per group). Shown are (e) the accumulation of SSE‐DiR in WT or *Apoe^−/−^* mice analyzed by the IVIS imaging system, f) the quantification of the fluorescence signals in (e), g) the internalization of SSE‐DiR by CD11c^+^ DCs in the inguinal lymph nodes of WT and *Apoe^−/−^* mice 24 h after subcutaneous injection, and h) representative histograms of SSE‐DiR internalization by CD11c^+^ DCs. (n = 4). i–l) WT or *Apoe^−/−^* mice were subcutaneously injected with the SSE vaccine on days 0 and 7, and the immune responses were analyzed on day 14. (n = 4 mice per group). Shown are i) representative flow cytometry scatter plots and j) quantitative analyses of E7‐specific CD8^+^ T cells among PBMCs on day 14. k,l) Representative flow cytometry scatter plots and quantification of IFN‐γ‐producing CD8^+^ T cells among PBMCs on day 14. Data represent mean ± SEM. Data were analyzed by two‐way ANOVA with Dunnett's multiple comparisons test (d) or two‐sided unpaired Student's *t*‐test (f,g,j,l).

We next sought to elucidate the mechanism underlying the superior delivery and potency of the SSE vaccine. Given that modulating the protein corona has emerged as a promising strategy to enhance nanoparticle delivery, we investigated whether the efficacy of the SSE vaccine depended on ApoE, a key protein known to interact with PS80 of SSE.^[^ ^23^, ^24^, ^25^, ^26^ ^]^ To assess this, we incubated SSE with mouse serum and analyzed the proteins bound to SSE. As shown in **Figure** **5**a, ApoE was detected in the protein corona of SSE. Additionally, molecular docking confirms that polysorbate 80 binds to a pocket in ApoE (Figure S15, Supporting information).

To evaluate the functional role of ApoE in SSE vaccine delivery, we incubated SSE with serum from ApoE‐deficient (*Apoe^−/−^*) mice and observed a 17.7% reduction in cellular internalization by BMDCs compared to SSE incubated with serum from wild‐type (WT) mice (Figure 5b; Figure S16, Supporting Information). Confocal microscopy further validated the role of ApoE in promoting SSE internalization (Figure 5c,d). Furthermore, ApoE deficiency impaired SSE accumulation in draining lymph nodes in vivo. Within the first 2 h, fluorescence signals in the inguinal lymph nodes of *Apoe^−/−^* mice were slightly weaker than those in WT mice (Figure S17a,b, Supporting Information). At 24 h post‐injection, the fluorescence intensity in the inguinal lymph nodes of WT mice was significantly higher than that of *Apoe^−/−^* mice (Figure 5e,f). ApoE deficiency also resulted in a reduced number of SSE‐positive APCs and other immune cells in the lymph nodes (Figure 5g,h; Figure S17c–i, Supporting Information).

We next examined whether ApoE influenced the T‐cell responses elicited by the SSE vaccine. Indeed, *Apoe^−/−^* mice exhibited significantly lower numbers of antigen‐specific CD8^+^ T cells and cytokine‐producing CD8^+^ T cells compared to WT mice following immunization with SSE‐E7/CpG (Figure 5i–l). Given that LDLR serves as the receptor for ApoE,^[^ ^27^, ^28^ ^]^ we also investigated whether LDLR deficiency affected SSE vaccine potency. Similar to ApoE deficiency, LDLR deficiency impaired SSE accumulation in draining lymph nodes (Figure S18a,b, Supporting Information) and reduced SSE‐DiR internalization by immune cells (Figure S18c, Supporting Information). Additionally, LDLR‐deficient (*Ldlr^−/−^*) mice exhibited weaker antigen‐specific and cytokine‐producing CD8^+^ T‐cell responses compared to WT mice after SSE‐E7/CpG immunization (Figure S18d–h, Supporting Information). Overall, our findings demonstrate that the SSE vaccine induces potent T‐cell responses in an ApoE‐dependent manner, highlighting the critical role of the protein corona in mediating efficient vaccine delivery.

### 2.6 SSE Vaccine Facilitates CD8^+^ T Activation in a Squalene‐Dependent Manner

**Figure 6:**
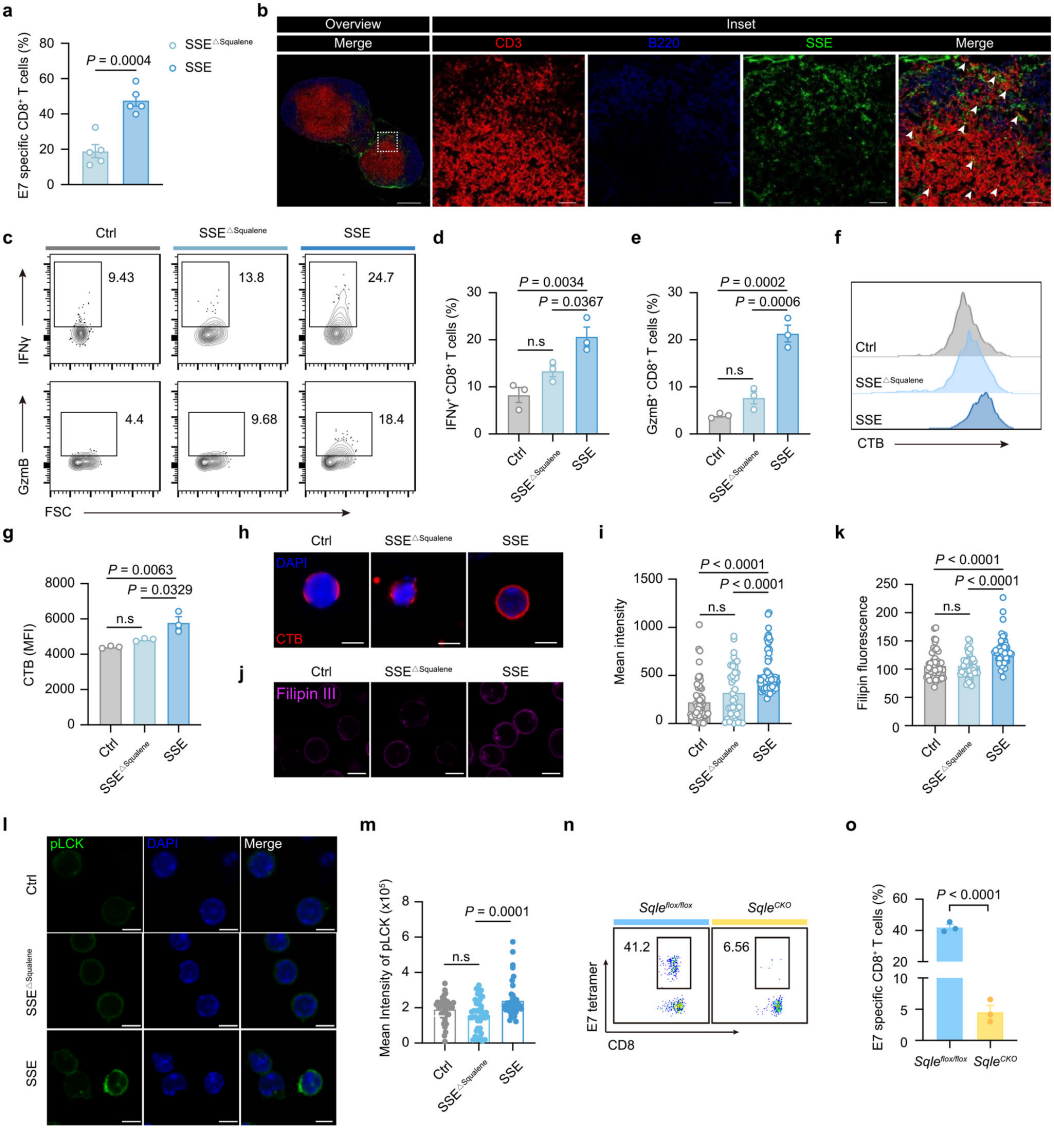
Squalene promotes CD8^+^ T‐cell activation via cholesterol synthesis. a) C57BL/6 mice were treated with the indicated vaccines on days 0 and 7. On day 14, quantitative analyses of E7‐specific CD8^+^ T‐cells among PBMCs. (n = 5 mice per group). b) Immunofluorescence of inguinal lymph nodes 24 h after subcutaneous injection of SSE (DiD, green; CD3, red; B220, blue). Scale bars represent 500 µm (overview) and 50 µm (inset). c–e) Cytokine and cytolytic granule production of CD8^+^ T cells stimulated with 2 µg mL^−1^ anti‐CD3/CD28. (n = 3 experimental replicates per group). f,g) Representative flow cytometry scatter plots and quantitative analyses of CTB on CD8^+^ T cells by flow cytometry. (n = 3 experimental replicates per group). h) Immunofluorescence staining for CTB on the cell membrane. Scale bars, 4 µm. i) Fluorescence quantification of CTB staining in h. (n = 50 cells). j,k) Membrane cholesterol quantification of CD8^+^ T cells treated with indicated formulations by filipin III staining. j) Immunofluorescence staining images for filipin III on the cell membrane. Scale bars, 5 µm. k) Fluorescence quantification of filipin III staining in j. (n = 50 cells). l) Confocal images of phosphorylated LCK (pLCK) in CD8^+^ T cells treated with SSE or SSE^△Squalene^. Scale bars, 5 µm. m) Fluorescence quantification of pLCK staining in l. (n = 50 cells). n,o) Representative flow cytometry scatter plots and quantitative analyses of antigen‐specific CD8^+^ T cells among PBMCs on day 14 after immunization with SSE. (n = 3 mice per group). Data represent mean ± SEM. Data were analyzed by two‐sided unpaired Student's *t*‐test (a,o) or one‐way ANOVA with Tukey's multiple comparisons test (d,e,g,i,k,m).

Given the unexpected potency of SSE vaccines, we hypothesized that mechanisms beyond enhanced lymph node drainage may also contribute to their immune activation. Because the surface composition of SSE determined the protein corona and lymph node drainage efficiency (Figure 2a; Figure S19, Supporting Information), we next focused on the internal squalene, whose role was still unclear. We hypothesized that squalene might modulate the efficacy of the SSE vaccine. To test this hypothesis, we prepared SSE vaccines and squalene‐deficient SSE vaccines (denoted as SSE^△Squalene^), both of which exhibit similar sizes and loading doses of antigen peptide and adjuvant (Figure S20a–c, Supporting Information). Mice immunized with SSE^△Squalene^ exhibited reduced total CD8^+^ T cell proportions, diminished antigen‐specific and cytokine‐producing CD8^+^ T cell induction compared to the SSE vaccine (**Figure** **6**a; Figure S20d–f, Supporting Information). However, no differences were observed between SSE and SSE^△Squalene^ in lymph node drainage efficiency, phagocytosis by immune cells, or in vitro BMDC internalization preference (Figure S20g–j, Supporting Information). Furthermore, squalene deficiency did not affect DC activation or antigen presentation in vivo (Figure S20k,l, Supporting Information). These findings collectively indicate that squalene enhances the immunogenicity of the SSE vaccine, independent of lymph node drainage or APC activation.

Notably, immunofluorescence revealed colocalization of SSE with T cells, with significantly greater internalization of SSE by T cells compared to other formulations (Figure 6b; Figure S2a, Supporting Information). Previous studies have demonstrated that lipid rafts are critical regulators of TCR signaling and T cell activation,^[^ ^29^, ^30^, ^31^, ^32^ ^]^ and squalene is an intermediate in the synthesis of cholesterol, a major structural component of lipid rafts.^[^ ^33^, ^34^ ^]^ These insights prompted us to investigate the role of squalene in modulating lipid rafts and T cell activation.

To investigate SSE regulation on T cells, we first examined the SSE internalization by T cells in vitro. In comparison, we used 200‐nm SE with the same composition as the control group. The results showed that T cells internalized more SSE compared with SE (Figure S21, Supporting Information). In the presence of anti‐CD3/CD28 stimulation, SSE significantly enhanced CD8⁺ T cell activation, as indicated by IFNγ and granzyme B secretion, whereas SSE^△Squalene^ failed to elicit a comparable response (Figure 6c–e). Notably, in the absence of anti‐CD3/CD28, both SSE and SSE^△Squalene^ induced CD8⁺ T cell activation at levels comparable to the PBS control (Figure S22, Supporting Information), suggesting that SSE alone does not directly activate CD8⁺ T cells, but rather sensitizes them to activation. We employed terbinafine, an inhibitor of squalene epoxidase, to block cholesterol synthesis regulated by squalene in CD8^+^ T cells. The result showed that terbinafine reduced T cell activation promoted by SSE (Figure S23, Supporting Information).

To investigate the underlying mechanism, we employed cholera toxin subunit B (CTB) to label lipid rafts and found that SSE treatment markedly increased lipid raft enrichment on CD8⁺ T cell membranes relative to naïve cells or those treated with SSE^△Squalene^ (Figure 6f–i). Additionally, the membrane cholesterol level of CD8^+^ T cells treated with SSE, but not SSE^△Squalene^, was substantially higher than that of untreated controls (Figure 6j,k). Moreover, we found elevated phosphorylated LCK, a T‐cell receptor (TCR) signaling protein in lipid rafts, in SSE‐treated CD8^+^ T cells (Figure 6l,m). Together, these findings indicate that squalene regulates membrane cholesterol levels to facilitate lipid raft formation and thereby enhance TCR signaling.

To further elucidate the role of squalene in T cell activation, we generated T cell‐specific *Sqle* knockout (*Sqle^CKO^*) mice, as *Sqle* encodes squalene epoxidase, a key enzyme involved in the conversion of squalene to cholesterol.^[^ ^35^ ^]^ We investigated the effect of SSE on lipid raft formation in *Sqle^CKO^* CD8^+^ T cells in vitro. The results showed that SSE enhanced lipid raft in *Sqle^flox/flox^* CD8^+^ T cells but not in those from *Sqle^CKO^* mice (Figure S24, Supporting Information). Notably, the lipid raft in CD8^+^ T cells incubated with SSE^△Squalene^ was comparable with that incubated with SSE in *Sqle^CKO^* CD8^+^ T cells. These results suggested that the change of lipid raft is dependent on squalene from SSE. Following immunization with SSE vaccines, *Sqle^CKO^* mice exhibited markedly reduced frequencies of antigen‐specific, cytokine‐producing CD8^+^ T cells, along with diminished cytolytic activity, compared to the *Sqle^flox/flox^* controls (Figure 6n,o; Figure S25, Supporting Information). Collectively, these findings reveal a previously unrecognized role for squalene in promoting CD8⁺ T cell activation via lipid raft modulation, highlighting its importance in generating potent T cell responses.

### 2.7 SSE Vaccine Induces Potent Therapeutic Effects

**Figure 7:**
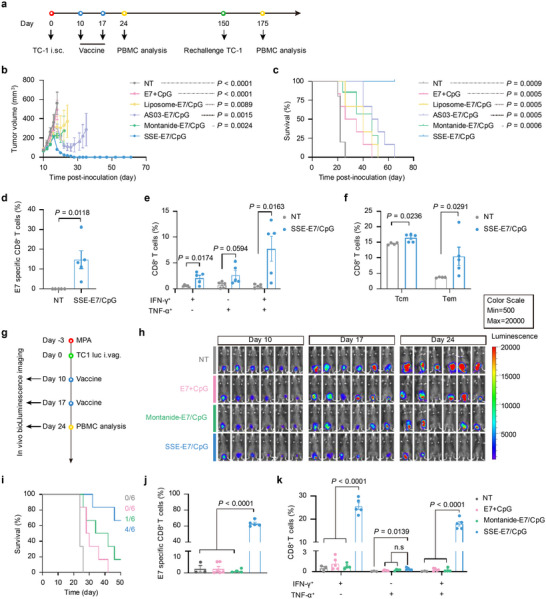
SSE‐E7/CpG exhibits potent therapeutic effects on subcutaneous and orthotopic TC‐1 tumors. a) The dosing regimen for treating the subcutaneous TC‐1 tumor model. b) Average growth curves of subcutaneous TC‐1 tumors in C57BL/6 mice treated with the indicated formulations (n = 6 mice per group). c) The survival curves of animals treated with the indicated formulations. d) Percentages of the E7‐specific CD8^+^ T cells in PBMCs from the rechallenged mice. (n = 5 mice per group). e) Cytokine‐producing CD8^+^ T cells in PBMCs from the rechallenged mice, and f) memory CD8^+^ T cells in PBMCs on day 175. (n = 5 mice per group). g) The dosing regimen for treating the orthotopic TC‐1 tumor model. h) The tumor burden was monitored over time using an IVIS imaging system after treatment with indicated formulations (n = 6 mice per group). i) Survival of orthotopic tumor‐bearing mice after treatment with indicated formulations. j) Percentage of E7‐specific CD8^+^ T cells, and k) cytokine‐producing CD8^+^ T cells among PBMCs in orthotopic tumor‐bearing mice on day 24. (n = 3–6 mice per group). Data represent mean ± SEM. Data were analyzed by two‐way ANOVA with Dunnett's multiple comparisons test (b), the log‐rank (Mantel–Cox) test (c), two‐sided unpaired Student's *t*‐test (d–f), or one‐way ANOVA with Tukey's multiple comparisons test (j,k).

To evaluate the anti‐tumor efficacy of SSE‐E7/CpG, we first established a TC‐1 tumor model in C57BL/6 mice by subcutaneously inoculating 2 × 10⁵ TC‐1 cells on day 0 (**Figure** **7**a). Mice were then immunized on days 10 and 17 with different formulations containing 3 µg per dose of E7 and 1 µg per dose of CpG. Treatment with soluble E7 and CpG led to moderate tumor suppression, whereas liposomes, Montanide, and AS03 showed comparable, but superior, tumor inhibition compared with the soluble group. Notably, SSE‐E7/CpG demonstrated the most significant tumor growth suppression (Figure 7b,c; Figure S26a,b, Supporting Information). On day 24, SSE‐E7/CpG induced a higher frequency of antigen‐specific CD8^+^ T cells than other formulations (Figure S26c,d, Supporting Information). Mice treated with SSE‐E7/CpG experienced slight body weight loss but recovered quickly (Figure S26e, Supporting Information). Histological analysis showed no notable changes in major organs (Figure S27a, Supporting Information), and biomedical markers from mice injected with either empty SSE or SSE‐E7/CpG were comparable to those of control mice (Figure S27b, Supporting Information), confirming the vaccine's safety.

By day 150, five mice immunized with SSE‐E7/CpG remained tumor‐free. These cured mice were rechallenged with 2 × 10⁵ TC‐1 cells on the contralateral side, alongside age‐matched control mice. While all control mice developed tumors, the rechallenged mice remained tumor‐free (Figure S28a–d, Supporting Information). Peripheral blood analysis 20 days post‐rechallenge revealed that SSE‐E7/CpG‐immunized mice maintained high levels of antigen‐specific and cytokine‐producing CD8^+^ T cells (Figure 7d,e; Figure S28e,f, Supporting Information). Moreover, SSE‐E7/CpG increased populations of effector memory (CD44^+^CD62L^−^, Tem) and central memory (CD44^+^CD62L^+^, Tcm) CD8^+^ T cells in peripheral blood (Figure 7f; Figure S28g, Supporting Information). These findings demonstrate that SSE‐E7/CpG elicits strong and durable anti‐tumor immunity.

To further validate its therapeutic efficacy, we tested SSE‐E7/CpG in an orthotopic cervical cancer model (Figure 7g). Mice were immunized with different formulations on days 10 and 17, and tumor progression was monitored via bioluminescence imaging on days 10, 17, and 24. Soluble antigen treatment led to moderate tumor suppression, while Montanide exhibited better tumor inhibition, with one out of six mice becoming tumor‐free. Remarkably, SSE‐E7/CpG induced the strongest tumor suppression, with four out of six mice achieving complete tumor regression (Figure 7h,i). Additionally, SSE‐E7/CpG elicited a higher frequency of antigen‐specific and cytokine‐producing CD8^+^ T cells compared to other formulations (Figure 7j,k; Figure S29, Supporting Information).

Encouraged by the potent effects of SSE‐E7/CpG, we next evaluated SSE in a refractory melanoma tumor model. Mice were immunized with different formulations containing multiple antigen peptides identified in melanoma cells, including M27, M30, and Trp1.^[^ ^36^, ^37^ ^]^ Compared with vaccines in the form of liposomes, Montanide, and AS03, SSE‐multiple B16F10 antigens/CpG (denoted as SSE‐multiAgs/CpG) elicited a stronger cytokine‐producing T‐cell response and enhanced cytolytic T‐cell activity (Figure S30a–c, Supporting Information). To assess the therapeutic efficacy of SSE‐multiAgs/CpG, we established melanoma tumors in C57BL/6 mice by subcutaneously inoculating B16F10 cells on day 0 and immunized them with various formulations containing 9 µg per dose of each antigen peptide and 3 µg per dose of CpG on days 8 and 15. Untreated mice reached the endpoint before day 20. As expected, SSE‐multiAgs/CpG exhibited the strongest tumor regression (Figure S30d–f, Supporting Information), while other formulations provided only moderate tumor suppression. These findings highlight the significant anti‐tumor potential of SSE across multiple tumor models, suggesting its broad applicability in cancer immunotherapy.

## 3 Discussion

In this study, we developed an ultrasmall squalene‐based stable emulsion (SSE) vaccine that elicits potent T‐cell responses against diverse tumor antigens by simultaneously regulating DCs and T cells, ultimately leading to tumor suppression or even complete elimination.

Although emulsion‐based vaccine platforms, such as Montanide and AS03, are known to induce both humoral and cellular immunity, Montanide emulsions form a depot at the injection site, thereby inducing T‐cell exhaustion, whereas AS03 emulsions reside at the injection site to recruit immune cells, thereby limiting the immune response.^[^ ^12^, ^14^, ^15^, ^38^ ^]^ Smaller nanoparticles (below 5 nm) are transported to the bloodstream, leading to less accumulation in the lymph nodes. Nanoparticles larger than 50 nm are trapped in the tissue and, therefore, less efficiently reach the lymph nodes. Nanoparticles between 5 and 50 nm exhibit preferential trafficking into lymphatic vessels, thereby reaching lymph nodes efficiently.^[^ ^39^ ^]^ Previous studies have demonstrated that 20 nm nanoparticles can reach lymph nodes rapidly and accumulate at a higher concentration than 200 nm nanoparticles.^[^ ^16^ ^]^ Additionally, the small particles also enhanced DC activation.^[^ ^40^ ^]^ Observing that SSE with a ≈20 nm size accumulated in lymph nodes more efficiently than SE with ≈200 nm is consistent with previous reports.

Other nanoscale vaccine platforms, including nanodiscs and albumin‐based carriers, exhibit efficient lymph node delivery due to their small size, but their fixed surface properties limit the ability to fine‐tune delivery profiles and optimize immunogenicity.^[^ ^18^, ^19^ ^]^ In contrast, SSE offers a versatile platform in which surface properties can be precisely modulated by adjusting the ratios of different excipients without altering particle size (Figure 2b; Figure S1e, Supporting Information). This tunability enabled the identification of an optimized SSE formulation with superior performance. Notably, a stable emulsion (SE) formulation with an identical composition but larger particle size induced weaker T‐cell responses, further underscoring the critical importance of the ultrasmall size of SSE particles.

Mechanistically, this enhanced potency can also be partially attributed to its unique protein corona. Although direct profiling of the protein corona in the interstitial fluid after subcutaneous vaccination remains challenging, our data suggest that ApoE, which binds PS80 and is abundant in the interstitial space,^[^ ^41^ ^]^ plays an essential role in promoting vaccine transport to lymph nodes and internalization by DCs. ApoE deficiency abolished the immune responses induced by SSE vaccines. Furthermore, genetic knockout of LDLR significantly impaired SSE vaccine efficacy, substantiating the importance of ApoE‐mediated delivery. Together, SSE vaccines rapidly accumulate in lymph nodes due to their small size and unique protein corona, eliciting robust and durable immune responses with low peptide and CpG content.

Beyond DCs, we observed that the SSE vaccine was also internalized by T cells and directly facilitated their activation, indicating a dual mechanism of action. Lipid rafts are dynamic membrane microdomains that serve as signaling platforms, such as T cell activation, by concentrating key signaling molecules upon T cell receptor activation.^[^ ^42^ ^]^ Cholesterol metabolic alterations contributed to the formation of dysfunctional lipid rafts and reduced T cell proliferation.^[^ ^43^ ^]^ Our findings suggest that SSE enhances lipid rafts on the CD8^+^ T cell membrane and promotes T cell activation by altering membrane cholesterol, consistent with previous studies. Squalene itself had minimal observable effects on APCs under the conditions tested, likely due to masking by CpG or insensitivity of APCs to the squalene dose, consistent with previous findings.^[^ ^33^ ^]^ As far as we are aware, vaccines that systemically harness both DC and T cell modulation to maximize antitumor immunity have not been extensively explored. These findings provide critical insights for the design of effective vaccines.

The development of efficient and stable delivery systems is critical for peptide vaccines to induce strong antigen‐specific T cell responses. Our results show that the SSE preparation process is suitable for large‐scale production and stable storage. The small size (≈20 nm) enables efficient accumulation of SSE in draining lymph nodes, where T cells prime and boost. SSE is also amenable to loading with various tumor antigens, eliciting broad‐spectrum T cell responses. These results indicate that SSE is a versatile platform for cancer vaccines. Nonetheless, several limitations warrant consideration. Although the SSE vaccine elicited potent T cell responses and therapeutic effects in murine models, its translation to the human immune system remains to be evaluated. Moreover, while this study focuses on the subcutaneous administration route, which has been widely used for vaccine delivery,^[^ ^44^, ^45^ ^]^ other routes of administration, such as intramuscular injection or intratumoral injection, are also employed in certain circumstances, and their impact on immune activation warrants further investigation. Future studies should also evaluate the efficacy of SSE‐based vaccines in combination with immune checkpoint inhibitors or in highly immunosuppressive tumor microenvironments to fully unleash the therapeutic potential of T cells.

## 4 Conclusion

In conclusion, we developed an ultrasmall, tunable SSE vaccine platform capable of inducing broad‐spectrum T‐cell responses and outperforming current peptide vaccine delivery systems. Its favorable safety profile and scalable manufacturing process position it as a strong candidate for clinical development, including studies involving personalized neoantigen peptides. Furthermore, the dual regulation of DCs and T cells by SSE provides a conceptual framework for the design of next‐generation cancer vaccines that leverage multi‐pronged immune activation for enhanced therapeutic benefit.

## 5 Experimental Section

### Synthesis of Lipid‐Modified Antigens

All materials used in the study are listed in Table S3 (Supporting Information). Each antigen peptide was conjugated to a lipid tail before vaccine formulation, as reported previously.^[^ ^46^ ^]^ Briefly, 1,2‐dioleoyl‐sn‐glycero‐3‐phosphoethanolamine‐N‐[3‐(2‐pyridyldithio)‐propionate] (DOPE‐PDP) and each antigen peptide (1:1.1 molar ratio) were dissolved in dimethylformamide and allowed to react at room temperature for 3 h under magnetic stirring. The reaction mixture was then diluted 10 fold with distilled water and freeze‐dried overnight. The obtained conjugate was dissolved in DMSO and kept at −20 °C until use.

### Preparation of Solid Self‐Emulsifying Nanoparticles or Vaccines

Briefly, 20 mg TPGS, 15 mg PS80, and 5 mg the oil phase squalene were mixed at 75 °C for ≈1 min and transferred to room temperature. Then, 50 µL of ethanol was added to the mixture and dispersed onto 90 mg of solid carrier mannitol. The uniform mixture was dried at 40 °C in an oven for 6 h. The solid mixture was hydrated in 1 mL of water to obtain SSE.

To synthesize DiR‐labeled SSE, DiR in ethanol was incubated with the preformed SSE described above under magnetic stirring for 2 h at room temperature. The product was then added to a dialysis bag and dialyzed in phosphate‐buffered saline (PBS) at 4 °C under gentle shaking overnight.

To prepare SSE‐based vaccines, 20 µg of lipid‐antigen peptide was mixed with 20 mg TPGS, 15 mg PS80, and 5 mg squalene in ethanol, then combined with 90 mg mannitol and dried as described above. The solid mixture was dissolved in 1 mL of water containing 20 µg cholesterol‐cytosine phosphorothioate guanine (cho‐CpG) to obtain the SSE‐based vaccine (SSE‐antigen/CpG). The product was added to a dialysis bag (100 kDa) and dialyzed in PBS at 4 °C under gentle shaking overnight. SSE‐antigen/CpG was concentrated according to the administration volume. In some experiments, the solid mixture was directly hydrated in water without cho‐CpG to obtain SSE‐antigen.

### Preparation of Control Formulations

SE was prepared according to the SSE formulation, except for the omission of mannitol. TPGS, PS80, squalene, and lipid‐antigen were mixed in ethanol and DMSO, followed by direct injection into water containing cho‐CpG to obtain SE‐antigen/CpG. Liposomes were prepared using the ethanol injection method. Briefly, to obtain a liposomal vaccine, 1,2‐dioleoyl‐sn‐glycero‐3‐phosphocholine (DOPC), lipid antigen, and 1,2‐distearoyl‐sn‐glycero‐3‐phosphoethanolamine‐methoxypolyethyleneglycol‐2000 (DSPE‐PEG2000) were dissolved in ethanol. Then the mixture was injected into water containing cho‐CpG, followed by dialysis to remove unloaded antigen and CpG. To obtain DiR‐labeled liposomes, DiR in ethanol was incubated with the prepared liposomes described above for 2 h under magnetic stirring. To prepare the Montanide‐based vaccine, antigen peptides and CpG in PBS were thoroughly emulsified in an equal volume of Montanide ISA51 until a homogeneous mixture was formed. In some experiments, DiR was added to the mixture for imaging studies. The AS03 vaccine was prepared as described previously.^[^ ^47^ ^]^ It consisted of 21.38 mg mL^−1^ squalene, 23.72 mg mL^−1^ α‐tocopherol, and 9.72 mg mL^−1^ PS80 in PBS. The mixture was emulsified to obtain AS03. The prepared AS03 was diluted with an equal volume of PBS containing the antigen and cho‐CpG before administration. The prepared AS03 was diluted with an equal volume of PBS containing DiR to obtain DiR‐labeled AS03 for imaging studies.

### Characterization of Nanoparticles or Vaccines

The morphologies of SSE and SE were observed by transmission electron microscopy after negative staining with 2% uranyl acetate solution. All images were taken with the Hitachi HT‐7800. The DLS and zeta potential of nanoparticles were measured on a Zetasizer size analyzer. The encapsulation rates of E7 on SSE vaccines with different ratios of TPGS to PS80, SE vaccines, and liposomes were determined by silver staining, as previously reported.^[^ ^48^ ^]^ Briefly, nanoparticles were loaded onto a Tris‐Tricine SDS‐polyacrylamide gel for electrophoresis. The gel was then imaged after silver staining (ThermoFisher Scientific). SIINFEKL was labeled with FITC, and the encapsulation rates of SIINFEKL on the indicated formulations were measured by FITC fluorescence signals. The encapsulation rates of B16F10 tumor neoantigens (M27, M30, and Trp1) were measured by the BCA kit according to the manufacturer's instructions. The encapsulation rates of CpG were measured by gel electrophoresis.

### Cell Culture

TC1 and TC1‐luc cells were cultured in RPMI 1640 medium. B16F10 was cultured in Dulbecco's modified Eagle medium (DMEM). All media were supplemented with 10% fetal bovine serum (FBS) and 1% penicillin‐streptomycin solution. Immature BMDCs were prepared as described previously.^[^ ^46^ ^]^ On day 0, naïve C57BL/6 mice were euthanized to collect the femur and tibia. The entire bone marrow was flushed out. Cells were cultured in RPMI 1640 medium supplemented with 10% FBS, 1% penicillin‐streptomycin, 50 nM β‐mercaptoethanol, and 20 ng mL^−1^ GM‐CSF. The medium was added on day 3. Immature BMDCs were used on day 6. All cells were maintained in a humidified atmosphere containing 5% CO_2_ at 37 °C.

### Cellular Internalization and Cytotoxicity

To learn the cellular internalization efficiency of SSE, BMDCs were plated at 4 × 10^5^ cells per well in 24‐well plates overnight. SSE‐DiD (0.1 µg mL^−1^ DiD) was pretreated with PBS, WT or *Apoe^−/−^* serum and incubated with BMDCs for different lengths of time (2, 5, 10, 30 min). Then, the cells were washed three times with PBS and labeled with a PE anti‐mouse CD11c antibody. Flow cytometry was used to analyze the cellular internalization of SSE.

To directly visualize the cellular internalization of SSE, immature BMDCs were seeded at 2 × 10^5^ cells per well in confocal glass dishes and cultured overnight. SSE‐DiD (0.1 µg mL^−1^ DiD) was pretreated with PBS, WT, or *Apoe^−/−^* serum and incubated with BMDCs for different lengths of time. Then, BMDCs were washed three times with PBS before being stained with Hoechst. Images were taken by confocal microscopy.

For antigen internalization of BMDCs, immature BMDCs were plated as described above in confocal dishes and cultured overnight. BMDCs were incubated with SSE loaded with SIINFEK_(FITC)_L or free SIINFEK_(FITC)_L (0.025 µg mL^−1^ SIINFEK_(FITC)_L). Then, BMDCs were cultured for different lengths of time (2, 6, 24, 48 h) and washed three times with PBS. Cells were stained with Lysotracker Deep Red for 30 min at 37 °C. The nuclei were stained with Hoechst before confocal microscopy.

To learn the cytotoxicity, immature BMDCs were plated at 1 × 10^5^ cells per well in 96‐well plates overnight. Different doses of SSEs were added and further cultured for different lengths of time (2, 6, 12, 24, 48 h). Cell viability was assessed using the Cell Counting Kit‐8 (CCK‐8) according to the manufacturer's instructions.

### Analysis of Protein Corona

The protein corona on SSEs was analyzed using a previously reported method.^[^ ^49^ ^]^ Briefly, SSEs were prepared as described above and diluted in PBS to the desired concentration. Naive mouse serum was added to the formulation at a 1:1 volume ratio and incubated at 37 °C for 60 min. The obtained mixture was centrifuged at 17 000 g and 4 °C for 60 min using an ultracentrifuge. The supernatant was carefully removed, and the pellet was washed with PBS gently twice. ApoE was tested by Western blot. Briefly, to quantify ApoE absorbed on SSEs, the pellet obtained as described above was resuspended in 2 × loading buffer and then boiled at 95 °C for 10 min before being loaded onto an SDS‐polyacrylamide gel. After completing gel electrophoresis, the proteins were transferred to a 0.22 µm polyvinylidene fluoride membrane. The membrane was then blocked with 5% skim milk for 1 h, followed by overnight incubation with the primary antibody solution (ApoE, 1:1000) at 4 °C. The membrane was washed four times for 10 min in PBS containing 0.1% Tween 20, and horseradish peroxidase–linked goat anti‐rabbit IgG (1:5000) was used as the secondary antibody at room temperature for 1 h. Then, the membrane was washed 4 times for 10 min each in PBS containing 0.1% Tween 20. The blot signals were visualized using Tanon imaging before incubation with the Pierce ECL Western Blotting Substrate.

### Animals

WT C57BL/6 were from Vital River Laboratory Animal Technology Co., Ltd. *Apoe^−/−^* mice on a C57BL/6 background were from GemPharmatech Co., Ltd. *Ldlr^−/−^* mice on a C57BL/6 background were from Cyagen. T‐cell‐specific depletion of *Sqle* mice was obtained by crossing Sqleflx*^/flox^* mice with *CD4^cre^* mice. Six‐ to eight‐week‐old, gender‐matched mice were used in the study. All animal procedures were performed following the Guidelines for Care and Use of Laboratory Animals of Tsinghua University and approved by the Animal Ethics Committee of Tsinghua University (22‐KR3.G23‐1). Mice were housed in a 12 h light/12 h dark cycle with ad libitum access to food and water at a controlled temperature. Animals were euthanized by CO_2_ inhalation, followed by cervical dislocation when the tumor reached 15 mm in any dimension or when they became moribund with severe weight loss or unhealing ulceration. This limit was not exceeded at any point.

### Lymph Node Draining Studies

Naive C57BL/6 mice were subcutaneously administered with various formulations containing 3 µg per dose of DiR. After 24 h, mice were euthanized, and the draining lymph nodes were harvested for imaging on an IVIS Spectrum Imaging System (PE Lumina III). An image set (Ex: 740, Em: 790, f 2, 0.5 s) was collected. Living Image software was used to obtain and quantify the fluorescence. In some experiments, *Apoe^−/−^* mice or *Ldlr^−/−^* mice were used for lymph node‐draining studies.

After imaging, the inguinal lymph nodes were prepared as single‐cell suspensions by grinding with a syringe to analyze the internalization of formulations by different cell subsets. The cells were incubated with CD16/32 for 10 min at room temperature, and then stained with PE anti‐mouse CD11c, BV605 anti‐mouse F4/80, APC anti‐mouse B220, FITC anti‐mouse CD3, and DAPI for 20 min at 4 °C. All flow data were obtained using a BD LSRFortessa SORP flow cytometer. Data analysis was performed using the FlowJo software.

For the immunofluorescence study, naïve mice were subcutaneously administered various formulations containing 3 µg per dose of DiD. After 24 h, mice were euthanized to harvest the inguinal lymph nodes. The inguinal lymph nodes were fixed in 4% paraformaldehyde overnight after IVIS imaging. Then, inguinal lymph nodes were cut into 10 µm‐thick sections on a Leica freezing microtome. The sections were incubated with the blocking solution containing 5% BSA in PBS for 60 min at room temperature. Next, the sections were stained using PE anti‐mouse CD11c and FITC anti‐mouse F4/80 for 60 min at room temperature. The sections were then mounted on coverslips with Fluoromount‐G with DAPI. The sections were observed by confocal microscopy.

### APC Activation and Antigen Persistence in dLNs

Female C57BL/6 mice were subcutaneously injected with SSE‐E7/CpG and SE‐E7/CpG containing 3 µg per dose of E7 and 1 µg per dose of CpG. Mice were euthanized at different time points, and inguinal lymph nodes were excised and ground mechanically to prepare single‐cell suspensions. The suspension was centrifuged at 4 °C for 5 min. The cell pellet was used to assess antigen presentation and APC activation, while the supernatant was used to determine inflammatory cytokine levels in lymph nodes by enzyme‐linked immunosorbent assay (ELISA).

The cells were divided into two parts. To measure activation of antigen‐presenting cells (APCs), cells were stained using a mixture of antibodies (APC anti‐mouse CD80, PE‐cy7 anti‐mouse CD86, PE anti‐mouse CD11c, and BV605 anti‐mouse F4/80) and DAPI at 4 °C for 20 min. For antigen presentation by APCs, cells were stained using fluorescent antibodies, PE anti‐mouse CD11c, BV605 anti‐mouse F4/80, and APC OVA_257‐264_ (SIINFEKL) peptide bound to H‐2Kb monoclonal antibody. Data were collected using a BD LSRFortessa SORP flow cytometer. Data analysis was performed using the FlowJo software. Additionally, the supernatant obtained above was centrifuged at 12 000 rpm for 10 min, and the levels of IL‐12 and TNF‐α were measured by ELISA according to the manufacturer's instructions.

### Immunization Studies

Female C57BL/6 mice (6–8 weeks) were subcutaneously injected with various vaccines by a homologous prime‐boost regimen. Mice were primed on day 0 and boosted on day 7 with vaccines containing 3 µg per dose of E7 peptide and 1 µg per dose of CpG or 9 µg per dose of each peptide (M27, M30, and Trp1) and 3 µg per dose of CpG.

For tetramer staining, blood was collected on day 14, and red blood cells were depleted by ACK (Ammonium chloride potassium) lysing buffer. Cells were resuspended in cold FACS buffer (PBS with 2% FBS) and then blocked with CD16/32 antibody and stained using PE HPV16 E7 (RAHYNIVTF) tetramer for 30 min at room temperature, followed by 20 min staining of APC anti‐mouse CD8 and DAPI at 4 °C. Cells were washed twice with the FACS buffer and then resuspended in FACS buffer before analysis using a BD LSRFortessa SORP flow cytometer.

For intracellular cytokine staining, blood was collected on day 14, and red blood cells were depleted by ACK lysing buffer. Cells were washed with cold FACS buffer and resuspended in T cell media (RPMI 1640 media supplemented with 10% FBS, 1% penicillin‐streptomycin solution, 1 mM sodium pyruvate, 50 µM β‐mercaptoethanol, 100 µg mL^−1^ HEPES, and 1 × non‐essential amino acids). Cells were plated in the 96‐well round‐bottom plate and pulsed with 20 µg mL^−1^ antigen peptides (HPV E7_49‐57_, RAHYNIVTF; M27, LCPGNKYEM; M30, CSSVDWENVSPELNSTDQ; and Trp‐1_455‐463_ CTAPDNLGYM) for 2 h at 37 °C, followed by the addition of 3 µg mL^−1^ brefeldin A for 4 h. Cells were washed with cold FACS buffer. Then, cells were stained with APC anti‐mouse CD8 or APC anti‐mouse CD4 for 20 min at 4 °C. The stained cells were then permeabilized and fixed using the Fixation/Permeabilization kit according to the manufacturer's instructions. Then, Intracellular staining for PE anti‐mouse IFN‐γ and FITC anti‐mouse TNF‐α was performed, and cells were analyzed on a BD LSRFortessa SORP flow cytometer.

### Biosafety Studies

Naïve mice were subcutaneously treated with PBS, SSE‐E7/CpG, empty SSE, and Montanide‐E7/CpG on day 0, and the blood of mice was collected on day 3. The serum was used to detect biochemical markers (ALT, AST, BUN, and CRE). In the subcutaneous TC1 model, mice were subcutaneously treated twice with PBS, SSE‐E7/CpG, E7+CpG, and Montanide‐E7/CpG on days 10 and 17. Then, three mice from each group were euthanized on day 22, and major organs (heart, liver, spleen, lung, and kidney) were harvested for pathological analysis. The images were taken by a scanning system (3DHISTECH).

### Measurement of T‐Cell Cytotoxicity

Detection of T cell cytotoxicity in vitro was performed as reported previously.^[^ ^34^ ^]^ Mice were immunized subcutaneously with various formulations loaded with multiple antigens (M27, M30, and Trp1) and CpG on day 0 and day 7. On day 14, immunized mice were euthanized, and splenocytes were collected. Splenocytes and B16F10 cells were mixed in a ratio of 5:1. After 8 h, the cytotoxic efficiency was measured by quantifying the release of endogenous lactate dehydrogenase (LDH) from B16F10 cells using a CytoTox 96 Non‐Radioactive Cytotoxicity kit (Promega).

Detection of T cell cytotoxicity in vivo was performed as reported previously.^[^ ^50^ ^]^ Briefly, mice were immunized subcutaneously with various formulations loaded with E7_49_₋_57_ (RAHYNIVTF) and CpG on days 0 and 7. On day 14, splenocytes harvested from syngenic mice were pulsed with 20 µg mL^−1^ RAHYNIVTF peptide for 30 min at 37 °C (peptide‐pulsed) or left unpulsed (peptide‐free). The peptide‐pulsed cells were labeled with 1 µM CFSE, and peptide‐free cells were labeled with 0.1 µM CFSE at 37 °C for 10 min in a serum‐free medium. The two cell populations were mixed (1:1 ratio) and intravenously injected into immunized mice or age‐matched naive mice at a dose of 1 × 10^7^ cells per mouse. After 18 h, mice were euthanized, and splenocytes from each mouse were analyzed on a BD LSRFortessa SORP flow cytometer to quantify the percentages of peptide‐pulsed and peptide‐free cells.

The percent killing was calculated as follows^[^ ^51^ ^]^:

(1) $$100\;-\;\frac{A\;\%\;\mathrm{in}\;\mathrm{immunized}\;\mathrm{mice}/B\;\%\;\mathrm{in}\;\mathrm{immunized}\;\mathrm{mice}}{A\;\%\;\mathrm{in}\;\mathrm{naive}\;\mathrm{mice}/B\;\%\;\mathrm{in}\;\mathrm{naive}\;\mathrm{mice}}\;\times \;100$$

where A represents peptide‐pulsed cells, and B represents peptide‐free cells.

### CD8^+^ T‐Cell Isolation and Function Analysis

CD8^+^ T cells were isolated from the mouse spleen using a CD8^+^ T‐cell negative selection kit (Biolegend). To measure the effector function of CD8^+^ T cells, the isolated cells were pretreated with SSE or SSE^△Squalene^ for 6 h, and then activated with anti‐CD3 and anti‐CD28 in the presence of 10 ng mL^−1^ IL‐2 for 24 h. Afterward, the cells were stimulated with stimulation cocktails plus protein transporter inhibitors (ThermoFisher Scientific) for 4 h, and then stained with APC anti‐mouse CD8α and fixable viability dye eFluor 450. Next, cells were permeabilized and fixed using the Fixation/Permeabilization kit according to the manufacturer's instructions. Then, Intracellular staining for PE anti‐mouse IFN‐γ and PE‐cy7 anti‐mouse granzyme B (GzmB) was performed, and cells were analyzed on a BD LSRFortessa SORP flow cytometer.

To measure lipid rafts, CD8^+^ T cells were treated with SSE or SSE^△Squalene^ and stained with cholera toxin subunit b conjugated to Alexa Fluor 555 (CTB‐555) for 1 h. Cells were analyzed on a BD LSRFortessa SORP flow cytometer or imaged with confocal microscopy. Images were processed with Nikon NIS‐elements 6.1.

### Therapeutic Studies

For the therapeutic study on TC1 tumor models, C57BL/6 mice of age 6–8 weeks were inoculated subcutaneously on the right flank with 2 × 10^5^ TC1 cells in 100 µl of cold PBS. Tumors were allowed to grow for 10 days before treatment. TC1‐bearing mice were randomly divided into the following groups: No treatment, E7+CpG, Liposome‐E7/CpG, AS03‐E7/CpG, Montanide‐E7/CpG, and SSE‐E7/CpG. The mice were subcutaneously injected with different formulations containing 3 µg per dose of E7 and 1 µg per dose of CpG at the base of the tail on days 10 and 17. The mouse weight and tumor size were monitored every 2 or 3 days. The tumor volume was calculated as length × width^2^ × 0.5. Mice were euthanized when individual tumor sizes reached 15 mm in any dimension or when animals exhibited severe body weight or temperature loss. On day 24, peripheral blood was collected to test the T cell response by tetramer staining. On day 150, survival mice were rechallenged with 2 × 10^5^ TC1 cells in 100 µl of cold PBS on the left flank, and age‐ and gender‐matched naïve C57BL/6 mice were inoculated with 2 × 10^5^ TC1 cells as the control group. The tumor size was monitored once every two days. Memory T cells were analyzed as reported previously. Briefly, peripheral blood was collected for staining with antibodies (FITC anti‐mouse CD44, PE anti‐mouse CD62L, APC anti‐mouse CD8). E7‐specific CD8^+^ T cell percentage was determined by tetramer staining, and T cell functions were analyzed by intracellular cytokine staining.

For therapeutic studies using orthotopic TC1 tumor models, 6–8‐week‐old female C57BL/6 mice were subcutaneously injected with medroxyprogesterone (3 mg per mouse) to synchronize diestrus 3 days before tumor cell inoculation.^[^ ^52^ ^]^ On day 0, the animals were inoculated with 1 × 10^6^ TC1‐luc cells by intravaginal administration. On days 10 and 17, tumor‐bearing mice were intravenously injected with the indicated formulations containing 3 µg per dose of antigen peptide and 1 µg per dose of CpG. Bioluminescence from tumor cells was visualized using IVIS after intraperitoneal injection of luciferin on days 10, 17, and 24. On day 24, peripheral blood was collected, and E7‐specific CD8^+^ T cells were identified by tetramer staining. T‐cell functions were then analyzed using intracellular cytokine staining.

For therapeutic studies on B16F10 tumor‐bearing mice, 6–8‐week female C57BL/6 mice were inoculated subcutaneously with 1 × 10^5^ B16F10 tumor cells on the right flank on day 0. On day 8, tumor‐bearing mice were randomly assigned to various groups. Mice were subcutaneously injected with different formulations containing 9 µg per dose of each peptide (M27, M30, and Trp1) and 3 µg per dose of CpG on day 8 and day 15. Tumor growth was monitored every two days. Animals were euthanized when individual tumor sizes reached 15 mm in any dimension or when animals exhibited severe body weight or temperature loss. To analyze the levels of CD8^+^ T cell response, peripheral blood was collected from animals on day 23. Intracellular staining for PE anti‐mouse IFN‐γ and FITC anti‐mouse TNF‐α was performed as described above, and the cells were analyzed on a BD LSRFortessa SORP flow cytometer.

### Statistical Analysis

All animal studies were performed after randomization. Each experiment was repeated three times, and similar results were obtained. All values were expressed as the mean ± SEM. All statistical analyses were performed using GraphPad Prism 8.0. Statistical analysis was performed using the two‐sided unpaired Student's *t*‐test for the comparison of two groups, one‐way analysis of variance (ANOVA) with Tukey's multiple comparisons test for various groups, and two‐way ANOVA with Dunnett's multiple comparisons test for multiple‐factor analysis. Survival curves were generated using the Kaplan–Meier method and compared using the log‐rank (Mantel–Cox) test.

## Conflict of Interest

A patent application (2023102586420) has been filed based on the SSE platform for vaccine delivery, with X.S., J.H., and R.K. as inventors.

## Author Contributions

X.S. and R.K. designed the experiments. X.S., S.F., J.H., L.L., C.W., K.Y., and J.H. performed the experiments. X.S., X.X., and R.K. analyzed the data. X.S. and R.K. wrote the manuscript.

## Supporting information



Supporting Information

## Data Availability

The data that support the findings of this study are available from the corresponding author upon reasonable request.

## References

[1] T. N. Schumacher , R. D. Schreiber , Science 2015, 348, 69. 25838375 10.1126/science.aaa4971

[2] T. L. Darrow , C. L. Slingluff Jr. , H. F. Seigler , J. Immunol. 1989, 142, 3329. 2785141

[3] D. A. Braun , G. Moranzoni , V. Chea , B. A. McGregor , E. Blass , C. R. Tu , A. P. Vanasse , C. Forman , J. Forman , A. B. Afeyan , N. R. Schindler , Y. Liu , S. Li , J. Southard , S. L. Chang , M. S. Hirsch , N. R. LeBoeuf , O. Olive , A. Mehndiratta , H. Greenslade , K. Shetty , S. Klaeger , S. Sarkizova , C. B. Pedersen , M. Mossanen , I. Carulli , A. Tarren , J. Duke‐Cohan , A. A. Howard , J. B. Iorgulescu , et al., Nature 2025, 639, 474. 39910301 10.1038/s41586-024-08507-5 PMC11903305

[4] W. Liu , H. Tang , L. Li , X. Wang , Z. Yu , J. Li , Cell Prolif. 2021, 54, 13025. 10.1111/cpr.13025 PMC8088465 33754407

[5] D. Shae , J. J. Baljon , M. Wehbe , P. P. Christov , K. W. Becker , A. Kumar , N. Suryadevara , C. S. Carson , C. R. Palmer , F. C. Knight , S. Joyce , J. T. Wilson , ACS Nano 2020, 14, 9904. 32701257 10.1021/acsnano.0c02765 PMC7775800

[6] D. J. Schwartzentruber , D. H. Lawson , J. M. Richards , R. M. Conry , D. M. Miller , J. Treisman , F. Gailani , L. Riley , K. Conlon , B. Pockaj , K. L. Kendra , R. L. White , R. Gonzalez , T. M. Kuzel , B. Curti , P. D. Leming , E. D. Whitman , J. Balkissoon , D. S. Reintgen , H. Kaufman , F. M. Marincola , M. J. Merino , S. A. Rosenberg , P. Choyke , D. Vena , P. Hwu , N. Engl. J. Med. 2011, 364, 2119. 21631324 10.1056/NEJMoa1012863 PMC3517182

[7] Y. Shi , Y. Sun , S. Zhao , Z. Sun , M. Xia , Z. Zhong , F. Meng , Adv. Mater. 2025, 37, 2420630. 10.1002/adma.202420630 40489066

[8] Y. Sun , G. Cui , Y. Shi , B. Xu , L. Qu , F. Meng , Adv. Funct. Mater. 2024, 35, 2416147.

[9] T. Stack , Y. Liu , M. Frey , S. Bobbala , M. Vincent , E. Scott , Nanoscale Horiz. 2021, 6, 393. 33884386 10.1039/d0nh00679c PMC8127988

[10] J. Aucouturier , L. Dupuis , S. Deville , S. Ascarateil , V. Ganne , Expert Rev. Vaccines 2002, 1, 111. 12908518 10.1586/14760584.1.1.111

[11] E. van Doorn , H. Liu , A. Huckriede , E. Hak , Hum. Vaccin. Immunother. 2016, 12, 159. 26378866 10.1080/21645515.2015.1071455 PMC4962750

[12] Y. Hailemichael , Z. Dai , N. Jaffarzad , Y. Ye , M. A. Medina , X. F. Huang , S. M. Dorta‐Estremera , N. R. Greeley , G. Nitti , W. Peng , C. Liu , Y. Lou , Z. Wang , W. Ma , B. Rabinovich , R. T. Sowell , K. S. Schluns , R. E. Davis , P. Hwu , W. W. Overwijk , Nat. Med. 2013, 19, 465. 23455713 10.1038/nm.3105 PMC3618499

[13] S. Gnjatic , N. Bhardwaj , Nat. Med. 2013, 19, 397. 23558621 10.1038/nm.3113

[14] T. Zhao , Y. Cai , Y. Jiang , X. He , Y. Wei , Y. Yu , X. Tian , Signal Transduct. Target Ther. 2023, 8, 283. 37468460 10.1038/s41392-023-01557-7 PMC10356842

[15] C. Cohet , R. van der Most , V. Bauchau , R. Bekkat‐Berkani , T. M. Doherty , A. Schuind , F. T. Da Silva , R. Rappuoli , N. Garcon , B. L. Innis , Vaccine 2019, 37, 3006. 31031030 10.1016/j.vaccine.2019.04.048

[16] L. Tang , X. Yang , L. W. Dobrucki , I. Chaudhury , Q. Yin , C. Yao , S. Lezmi , W. G. Helferich , T. M. Fan , J. Cheng , Angew. Chem. Int. Ed. Engl. 2012, 51, 12721. 23136130 10.1002/anie.201205271 PMC4486261

[17] J. McCright , R. Naiknavare , J. Yarmovsky , K. Maisel , Front. Pharmacol. 2022, 13, 887402. 35721179 10.3389/fphar.2022.887402 PMC9203826

[18] H. Liu , K. D. Moynihan , Y. Zheng , G. L. Szeto , A. V. Li , B. Huang , D. S. Van Egeren , C. Park , D. J. Irvine , Nature 2014, 507, 519. 24531764 10.1038/nature12978 PMC4069155

[19] R. Kuai , L. J. Ochyl , K. S. Bahjat , A. Schwendeman , J. J. Moon , Nat. Mater. 2017, 16, 489. 28024156 10.1038/nmat4822 PMC5374005

[20] H. Hemmi , O. Takeuchi , T. Kawai , T. Kaisho , S. Sato , H. Sanjo , M. Matsumoto , K. Hoshino , H. Wagner , K. Takeda , S. Akira , Nature 2000, 408, 740. 11130078 10.1038/35047123

[21] S. K. Saini , K. Ostermeir , V. R. Ramnarayan , H. Schuster , M. Zacharias , S. Springer , Proc. Natl. Acad. Sci. U S A 2013, 110, 15383. 24003162 10.1073/pnas.1308672110 PMC3780906

[22] M. C. Feltkamp , H. L. Smits , M. P. Vierboom , R. P. Minnaar , B. M. de Jongh , J. W. Drijfhout , J. ter Schegget , C. J. Melief , W. M. Kast , Eur. J. Immunol. 1993, 23, 2242. 7690326 10.1002/eji.1830230929

[23] T. Cedervall , I. Lynch , S. Lindman , T. Berggard , E. Thulin , H. Nilsson , K. A. Dawson , S. Linse , Proc. Natl. Acad. Sci. U S A 2007, 104, 2050. 17267609 10.1073/pnas.0608582104 PMC1892985

[24] K. Liu , R. Nilsson , E. Lazaro‐Ibanez , H. Duan , T. Miliotis , M. Strimfors , M. Lerche , A. R. Salgado Ribeiro , J. Ulander , D. Linden , A. Salvati , A. Sabirsh , Nat. Commun. 2023, 14, 4007. 37414857 10.1038/s41467-023-39768-9 PMC10325984

[25] Y. Sakurai , K. Yoshikawa , K. Arai , A. Kazaoka , S. Aoki , K. Ito , Y. Nakai , K. Tange , T. Furihata , H. Tanaka , H. Akita , J. Control Release 2023, 353, 125. 36414194 10.1016/j.jconrel.2022.11.036

[26] S. A. Dilliard , Q. Cheng , D. J. Siegwart , Proc. Natl. Acad .Sci. U S A 2021, 118, 2109256118. 10.1073/pnas.2109256118 PMC8719871 34933999

[27] S. Ishibashi , J. Herz , N. Maeda , J. L. Goldstein , M. S. Brown , Proc. Natl. Acad. Sci. USA 1994, 91, 4431. 8183926 10.1073/pnas.91.10.4431 PMC43799

[28] A. Akinc , W. Querbes , S. De , J. Qin , M. Frank‐Kamenetsky , K. N. Jayaprakash , M. Jayaraman , K. G. Rajeev , W. L. Cantley , J. R. Dorkin , J. S. Butler , L. Qin , T. Racie , A. Sprague , E. Fava , A. Zeigerer , M. J. Hope , M. Zerial , D. W. Sah , K. Fitzgerald , M. A. Tracy , M. Manoharan , V. Koteliansky , A. Fougerolles , M. A. Maier , Mol. Ther. 2010, 18, 1357. 20461061 10.1038/mt.2010.85 PMC2911264

[29] A. Cherukuri , M. Dykstra , S. K. Pierce , Immunity 2001, 14, 657. 11420035 10.1016/s1074-7613(01)00156-x

[30] M. Dykstra , A. Cherukuri , H. W. Sohn , S. J. Tzeng , S. K. Pierce , Annu. Rev. Immunol. 2003, 21, 457. 12615889 10.1146/annurev.immunol.21.120601.141021

[31] T. Zech , C. S. Ejsing , K. Gaus , B. de Wet , A. Shevchenko , K. Simons , T. Harder , EMBO J. 2009, 28, 466. 19177148 10.1038/emboj.2009.6 PMC2657588

[32] M. Belabed , M. D. Park , C. M. Blouin , S. Balan , C. Y. Moon , G. Freed , M. Quijada‐Alamo , A. Peros , R. Mattiuz , A. M. Reid , N. Yatim , J. Boumelha , C. S. Azimi , N. M. LaMarche , L. Troncoso , A. Amabile , J. Le Berichel , S. T. Chen , C. M. Wilk , B. D. Brown , K. J. Radford , S. Ghosh , C. V. Rothlin , L. Yvan‐Charvet , T. U. Marron , D. J. Puleston , E. Wagenblast , N. Bhardwaj , C. Lamaze , M. Merad , Nat. Immunol. 2025, 26, 188. 39838105 10.1038/s41590-024-02065-8

[33] J. Surls , C. Nazarov‐Stoica , M. Kehl , C. Olsen , S. Casares , T. D. Brumeanu , PLoS One 2012, 7, 38733. 10.1371/journal.pone.0038733 PMC3378591 22723880

[34] W. Yang , Y. Bai , Y. Xiong , J. Zhang , S. Chen , X. Zheng , X. Meng , L. Li , J. Wang , C. Xu , C. Yan , L. Wang , C. C. Chang , T. Y. Chang , T. Zhang , P. Zhou , B. L. Song , W. Liu , S. C. Sun , X. Liu , B. L. Li , C. Xu , Nature 2016, 531, 651. 26982734 10.1038/nature17412 PMC4851431

[35] H. Yoshioka , H. W. Coates , N. K. Chua , Y. Hashimoto , A. J. Brown , K. Ohgane , Proc. Natl. Acad. Sci. U S A 2020, 117, 7150. 32170014 10.1073/pnas.1915923117 PMC7132291

[36] S. Kreiter , M. Vormehr , N. van de Roemer , M. Diken , M. Lower , J. Diekmann , S. Boegel , B. Schrors , F. Vascotto , J. C. Castle , A. D. Tadmor , S. P. Schoenberger , C. Huber , O. Tureci , U. Sahin , Nature 2015, 520, 692. 25901682 10.1038/nature14426 PMC4838069

[37] G. Ghanem , J. Fabrice , Mol. Oncol. 2011, 5, 150. 21324755 10.1016/j.molonc.2011.01.006 PMC5528278

[38] L. Baitsch , P. Baumgaertner , E. Devevre , S. K. Raghav , A. Legat , L. Barba , S. Wieckowski , H. Bouzourene , B. Deplancke , P. Romero , N. Rufer , D. E. Speiser , J. Clin. Invest. 2011, 121, 2350. 21555851 10.1172/JCI46102 PMC3104769

[39] P. Yousefpour , K. Ni , D. J. Irvine , Nat. Rev. Bioeng. 2023, 1, 107. 37772035 10.1038/s44222-022-00016-2 PMC10538251

[40] J. Xu , Q. Ma , Y. Zhang , Z. Fei , Y. Sun , Q. Fan , B. Liu , J. Bai , Y. Yu , J. Chu , J. Chen , C. Wang , Nat. Commun. 2022, 13, 110. 35013252 10.1038/s41467-021-27750-2 PMC8748771

[41] D. Reichl , T. M. Forte , J. L. Hong , D. N. Rudra , J. Pflug , J. Lipid Res. 1985, 26, 1399. 4086943

[42] K. Simons , D. Toomre , Nat. Rev. Mol. Cell Biol. 2000, 1, 31. 11413487 10.1038/35036052

[43] C. F. Jacobs , F. S. Peters , E. Camerini , G. Cretenet , J. Rietveld , B. V. Schomakers , M. van Weeghel , N. Hahn , S. G. S. Verberk , J. Van den Bossche , M. Langeveld , F. Kleijwegt , E. Eldering , N. Zelcer , A. P. Kater , H. Simon‐Molas , Cell Mol. Immunol. 2025, 22, 485. 40033083 10.1038/s41423-025-01262-1 PMC12041523

[44] A. Bouazzaoui , A. A. H. Abdellatif , Vaccine 2024, 19, 100500; 10.1016/j.jvacx.2024.100500 PMC11170481 38873639

[45] H. M. Kinnunen , R. J. Mrsny , J. Control Release 2014, 182, 22. 24631859 10.1016/j.jconrel.2014.03.011

[46] L. Luo , J. Li , X. Shen , X. Li , C. Peng , S. Li , R. Kuai , Nano Lett. 2024, 24, 15758. 39585971 10.1021/acs.nanolett.4c04557

[47] S. Morel , A. Didierlaurent , P. Bourguignon , S. Delhaye , B. Baras , V. Jacob , C. Planty , A. Elouahabi , P. Harvengt , H. Carlsen , A. Kielland , P. Chomez , N. Garcon , M. Van Mechelen , Vaccine 2011, 29, 2461. 21256188 10.1016/j.vaccine.2011.01.011

[48] A. Yeste , M. Nadeau , E. J. Burns , H. L. Weiner , F. J. Quintana , Proc Natl Acad Sci.U S A 2012, 109, 11270. 22745170 10.1073/pnas.1120611109 PMC3396465

[49] Z. Zhang , J. Guan , Z. Jiang , Y. Yang , J. Liu , W. Hua , Y. Mao , C. Li , W. Lu , J. Qian , C. Zhan , Nat. Commun. 2019, 10, 3561. 31395892 10.1038/s41467-019-11593-z PMC6687821

[50] J. Ramos da Silva , K. Bitencourt Rodrigues , G. Formoso Pelegrin , N. Silva Sales , H. Muramatsu , M. de Oliveira Silva , B. Porchia , A. C. R. Moreno , L. Aps , A. A. Venceslau‐Carvalho , I. Tombacz , W. L. Fotoran , K. Kariko , P. J. C. Lin , Y. K. Tam , M. de Oliveira Diniz , N. Pardi , L. C. de Souza Ferreira , Sci. Transl. Med. 2023, 15, abn3464. 10.1126/scitranslmed.abn3464 36867683

[51] D. L. Barber , E. J. Wherry , R. Ahmed , J. Immunol. 2003, 171, 27. 12816979 10.4049/jimmunol.171.1.27

[52] J. He , C. Wang , X. Fang , J. Li , X. Shen , J. Zhang , C. Peng , H. Li , S. Li , J. M. Karp , R. Kuai , Nat. Commun. 2024, 15, 8121. 39284814 10.1038/s41467-024-52104-z PMC11405680

