# Supplementary material for: Designer Solid Self‐Emulsifying Nanovaccines Enable Dual Modulation of Dendritic Cells and T Cells for Potent Antitumor Immunity
Source: Adv Sci (Weinh). 2025 Nov 7;13(5):e12139. doi: 10.1002/advs.202512139 (PMC12849924; doi:10.1002/advs.202512139)
Supplement: Supplementary file 1 — Supporting Information [file ADVS-13-e12139-s001.docx]

Supporting Information

**Designer solid self-emulsifying nanovaccines enable dual modulation of dendritic cells and T cells for potent antitumor immunity**

Xueying Shen^1,2^, Shiqi Fan^1,2^, Jia He^1,2^, Lanqing Luo^1,2^, Junyao Li^1,2^, Chengcheng Wu^1,2^, Kairu Yang^1,2^, Xiaojun Xia^3,4^, Rui Kuai^1,2^*


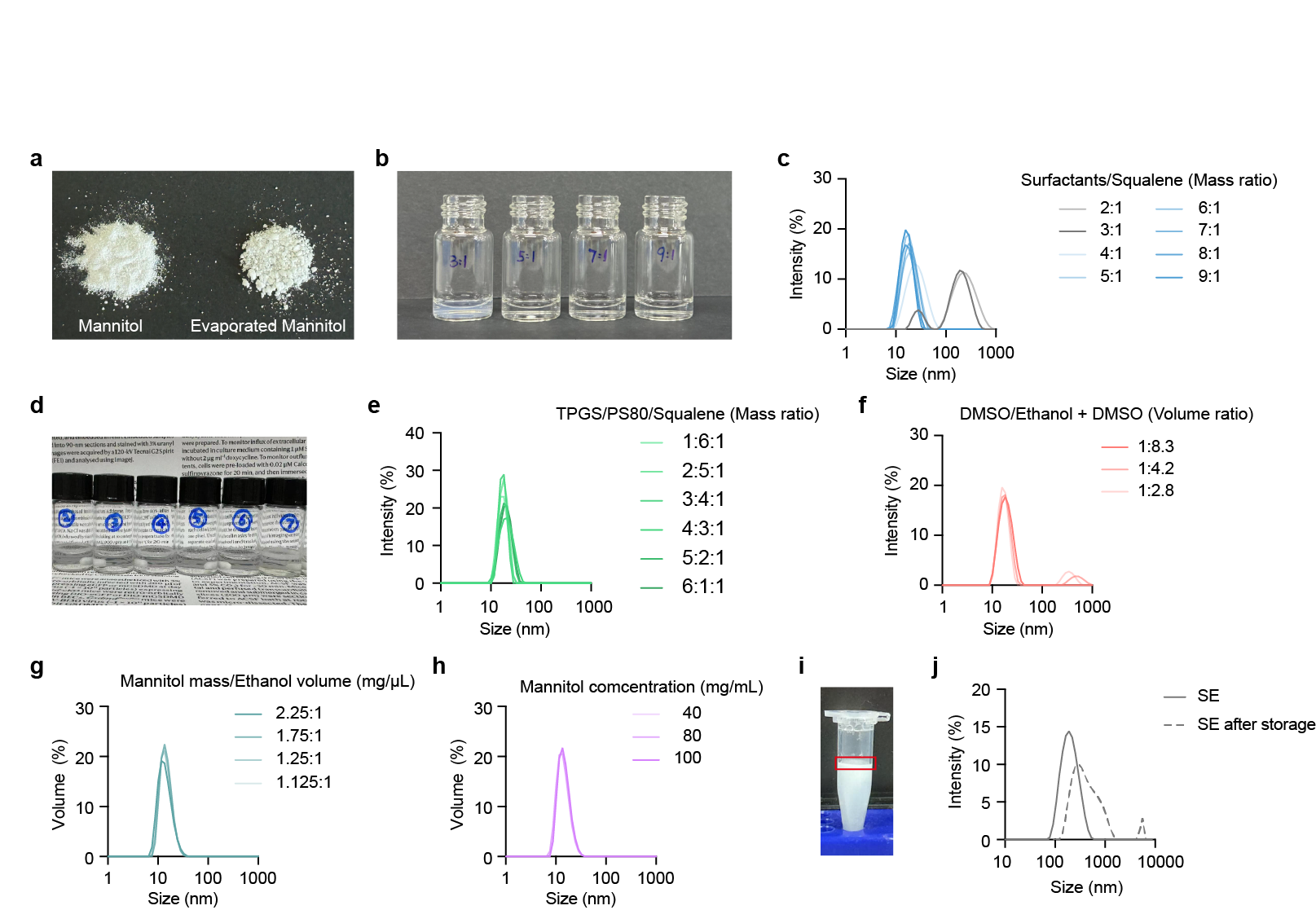


**Figure S1. Characterization of SSE formulations.** a) The image of mannitol and evaporated mannitol after adsorption of surfactants and squalene. The image (b) and size distribution (c) of SSE containing different ratios of surfactants (TPGS+PS80) and squalene. The image (d) and size distribution (e) of SSE containing different ratios of TPGS, PS80, and squalene. f) The size distribution of SSE prepared with different ratios of ethanol and DMSO. g) The size distribution of SSE with different amounts of mannitol. h) The size distribution of SSE with the addition of different volumes of water. The image (i) and size distribution (j) of SE after two weeks of storage at 4 ℃. The experiments were performed three times with similar results.


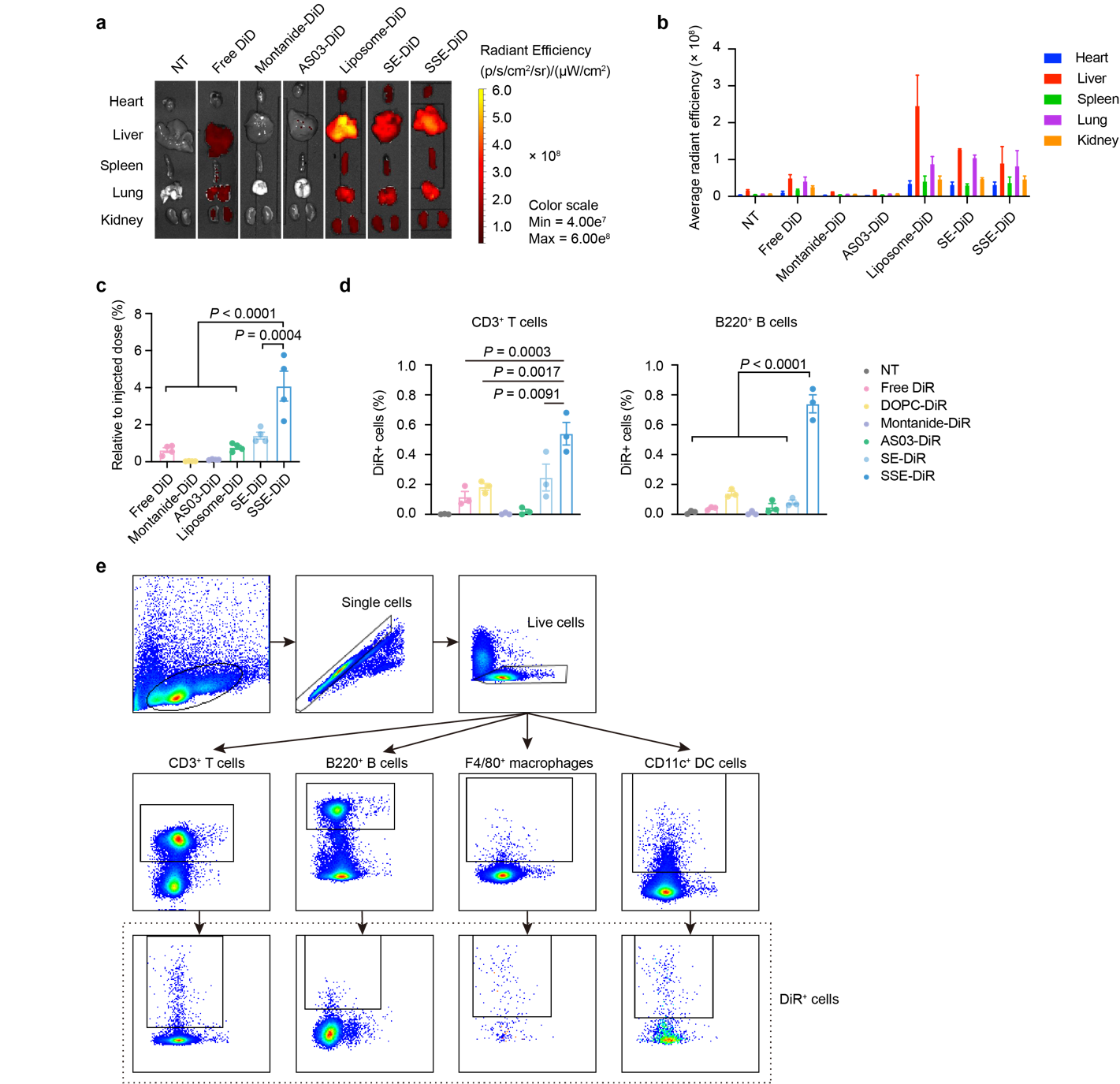


**Figure S2. The accumulation and internalization of various formulations.** a-c) Mice were injected with indicated formulations encapsulated with 3 μg DiD subcutaneously. a) The accumulation of various formulations in the main organs 24 h after injection. b) Quantitative results of average fluorescence intensity in (a). c) DiD accumulation in draining lymph nodes relative to injected dose. (n = 4 experimental replicates per group). C57BL/6 mice were subcutaneously injected with the indicated formulations labeled with DiR. At 24 h post-injection, inguinal lymph nodes were harvested, and the cellular uptake of indicated formulations was analyzed by flow cytometry. d) percentages of DiR^+^ cells among CD3^+^ T cells and B220^+^ B cells. (n = 3 experimental replicates per group). b) Gating strategies of DiR^+^ cells among different types of cells. Data represent mean ± SEM. Data were analyzed by one-way ANOVA with Tukey’s multiple comparisons test (c, d).


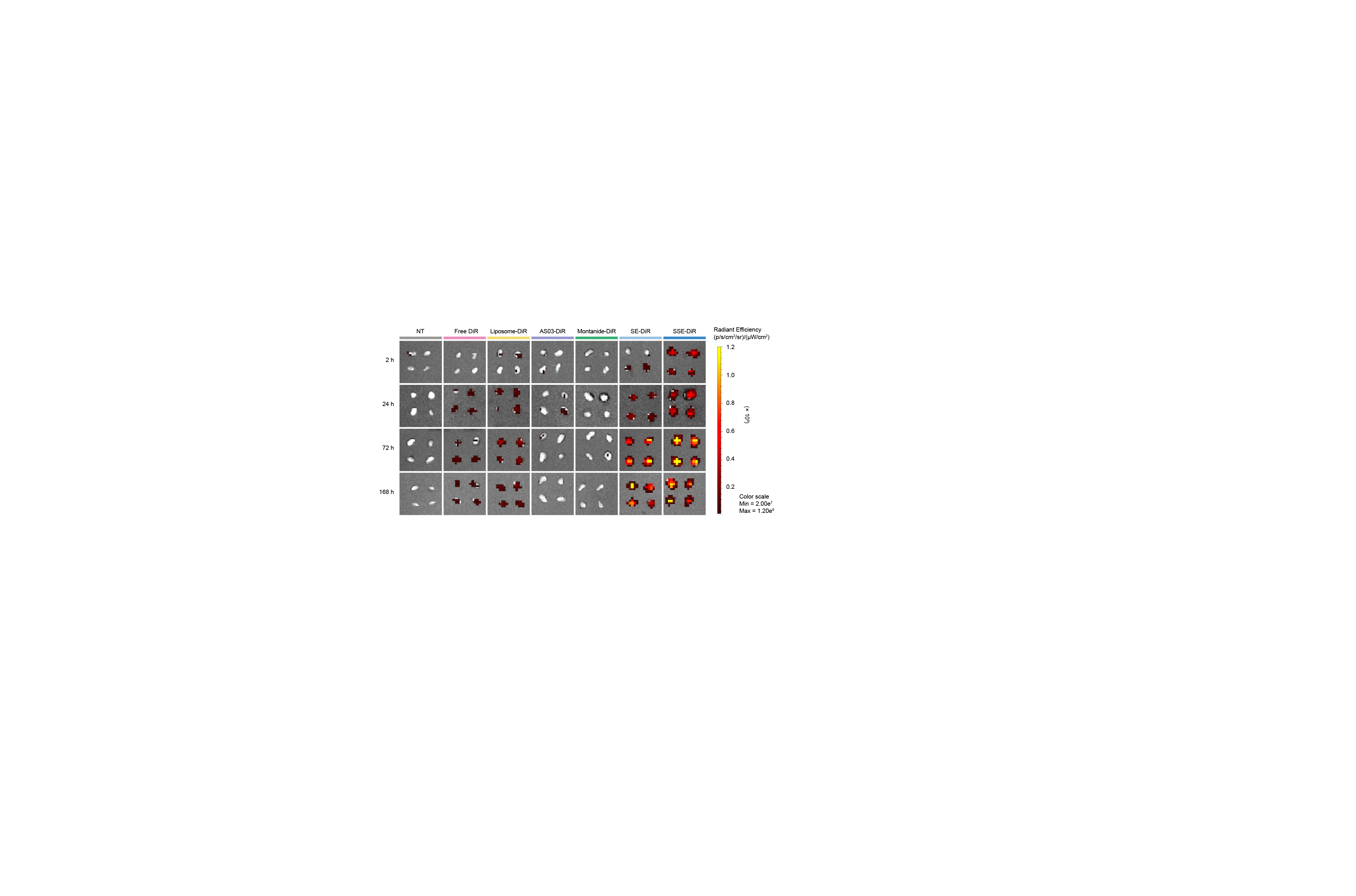


**Figure S3. The accumulation of various formulations in the inguinal lymph nodes over time after subcutaneous injection.**

**
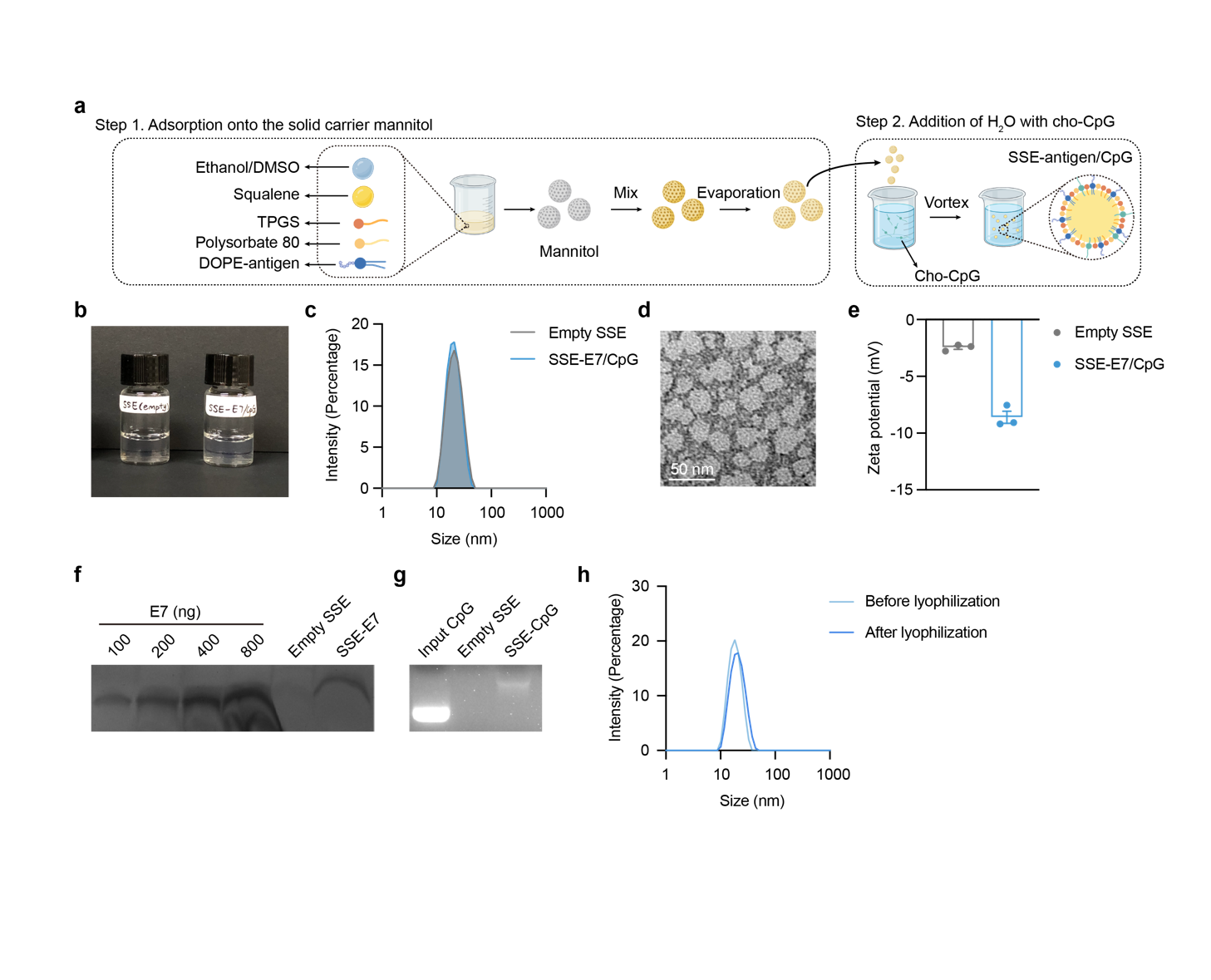
**

**Figure S4. Preparation and characterization of SSE-E7/CpG.** a) The schematic for the preparation of SSE-antigen/CpG. b) The image of empty SSE and SSE-E7/CpG. c) Size distribution of empty SSE and SSE-E7/CpG. d) The representative TEM image of SSE-E7/CpG. e) The zeta potentials of empty SSE and SSE-E7/CpG (n = 3 experimental replicates per group). The encapsulation efficiency of E7 (f) and CpG (g) was measured by gel electrophoresis. The experiments were performed three times. h) The size distribution of lyophilized SSE vaccines. Data represent mean ± SEM.

**
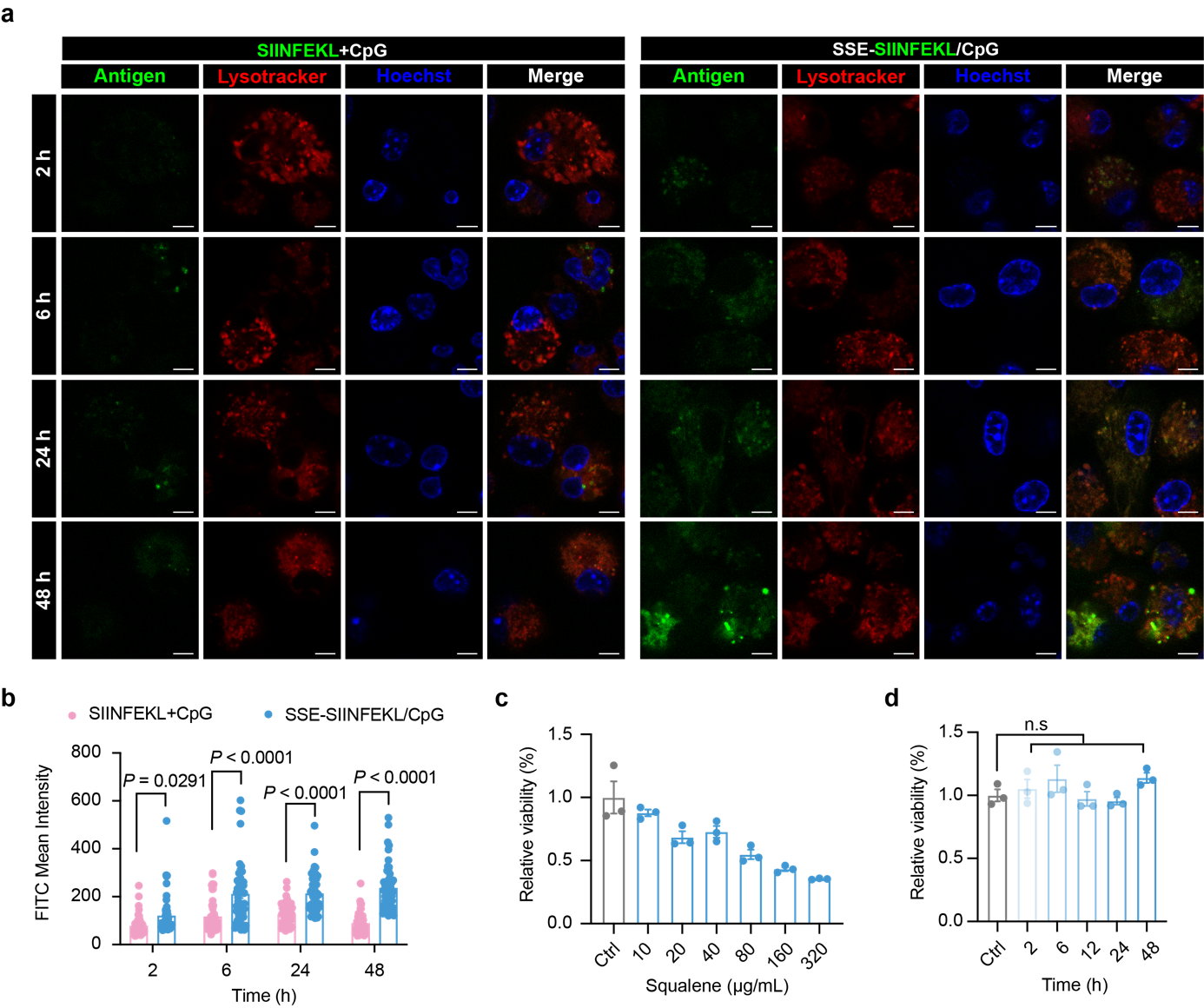
**

**Figure S5. Intracellular delivery of SIINFEKL.** BMDCs were incubated with SIINFEK_(FITC)_L+CpG or SSE-SIINFEK_(FITC)_L/CpG for different lengths of time, and the cellular uptake was monitored by confocal microscopy. a) Intracellular delivery profile of SIINFEK_(FITC)_L in BMDCs at different time points. b) The quantitative analyses of FITC fluorescence intensity in (a) (n = 50 cells). c, d) The viabilities of BMDCs after treatment with SSE vaccines at indicated concentrations and time points (n = 3 experimental replicates per group). Data represent mean ± SEM. Data were analyzed by two-way ANOVA with Dunnett’s multiple comparisons test (b) or one-way ANOVA with Tukey’s multiple comparisons test (d).

**
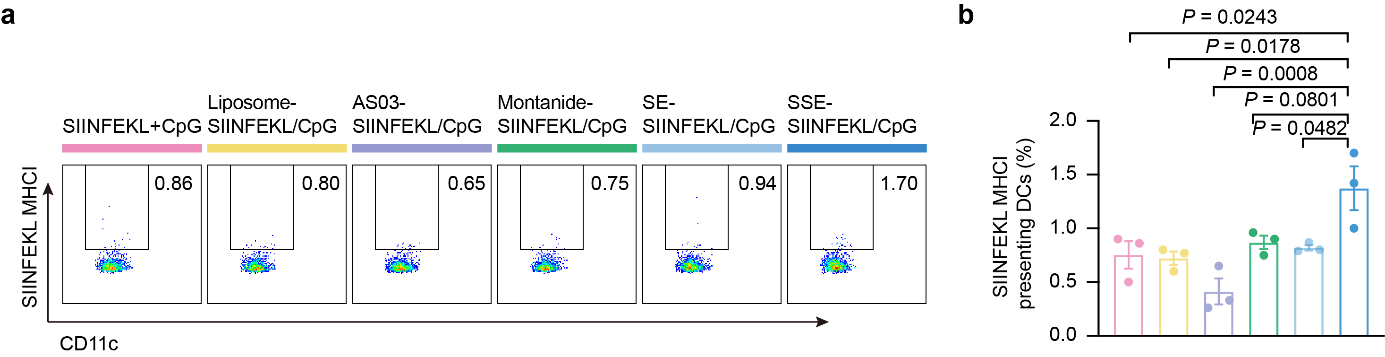
**

**Figure S6. The SIINFEKL-MHCI display on DCs in draining lymph nodes 24 h after injection of various formulations.** a) Representative flow cytometry scatter plots, and b) quantitative analyses of DCs displaying SIINFEKL-MHCI (n = 3 experimental replicates per group). Data represent mean ± SEM. Data were analyzed by one-way ANOVA with Tukey’s multiple comparisons test (b).


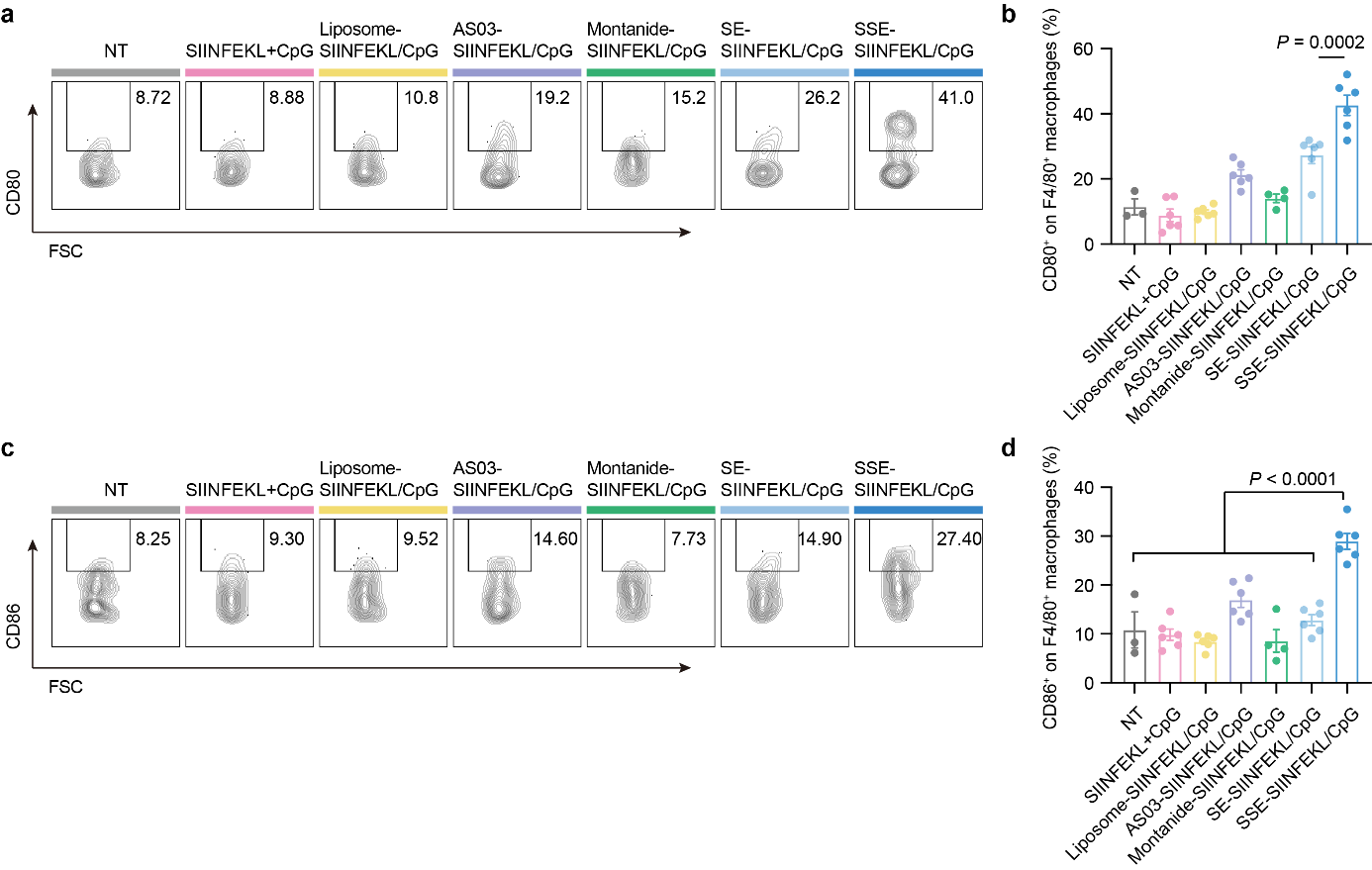


**Figure S7. Macrophage maturation in inguinal lymph nodes.** a, b) Representative flow cytometry scatter plots and quantitative analyses of CD80 expression on F4/80^+^ macrophages. c, d) Representative flow cytometry scatter plots and quantitative analyses of CD86 expression on F4/80^+^ macrophages. (n = 3 - 6 experimental replicates per group). Data represent mean ± SEM. Data were analyzed by one-way ANOVA with Tukey’s multiple comparisons test (b, d).


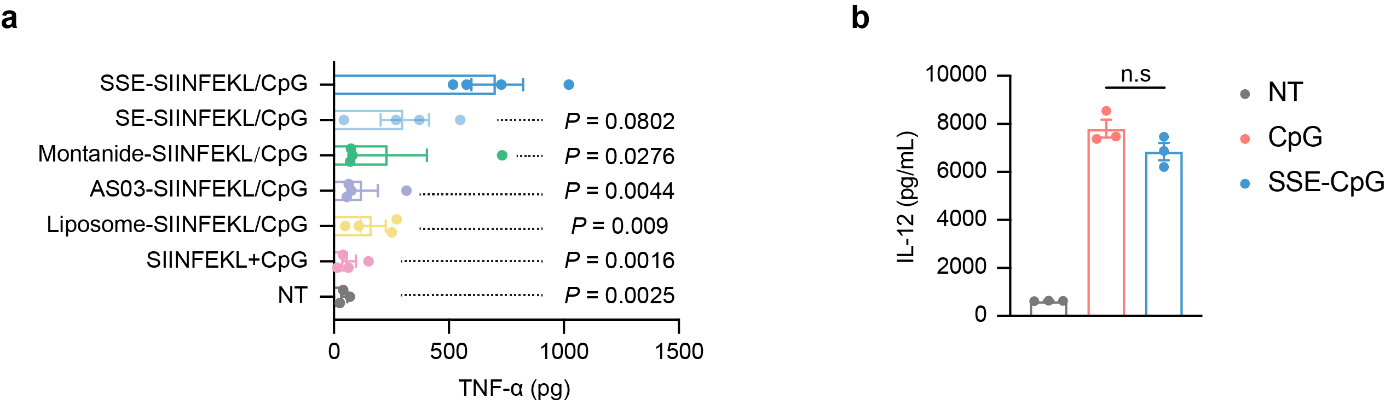


**Figure S8. Cytokine production activated by various formulations.** a) TNF-α production in inguinal lymph nodes 24 h after injection of various formulations. (n = 4 experimental replicates per group). b) BMDCs were incubated with 0.125 μg ml^-1^ CpG formulations for 24 h. The levels of IL-12 were measured by ELISA analysis. Data represent mean ± SEM. Data were analyzed by one-way ANOVA with Tukey’s multiple comparisons test.


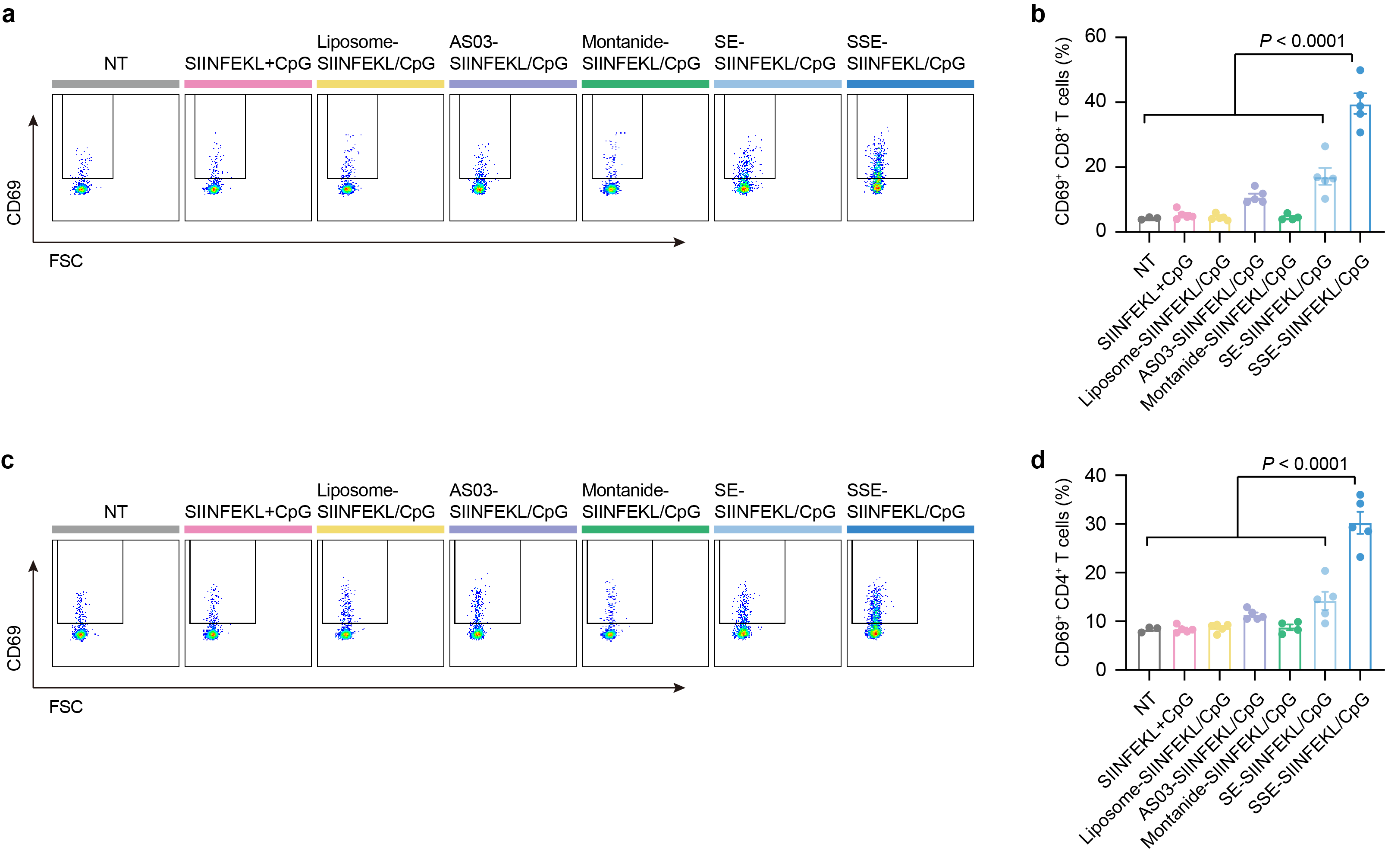


**Figure S9. T cell activation in inguinal lymph nodes 24 h after injection with various formulations.** a, b) Representative flow cytometry scatter plots and quantitative analyses of CD69 expression on CD8^+^ T cells. c, d) Representative flow cytometry scatter plots and quantitative analyses of CD69 expression on CD4^+^ T cells. (n = 3 -5 experimental replicates per group). Data represent mean ± SEM. Data were analyzed by one-way ANOVA with Tukey’s multiple comparisons test (b, d).


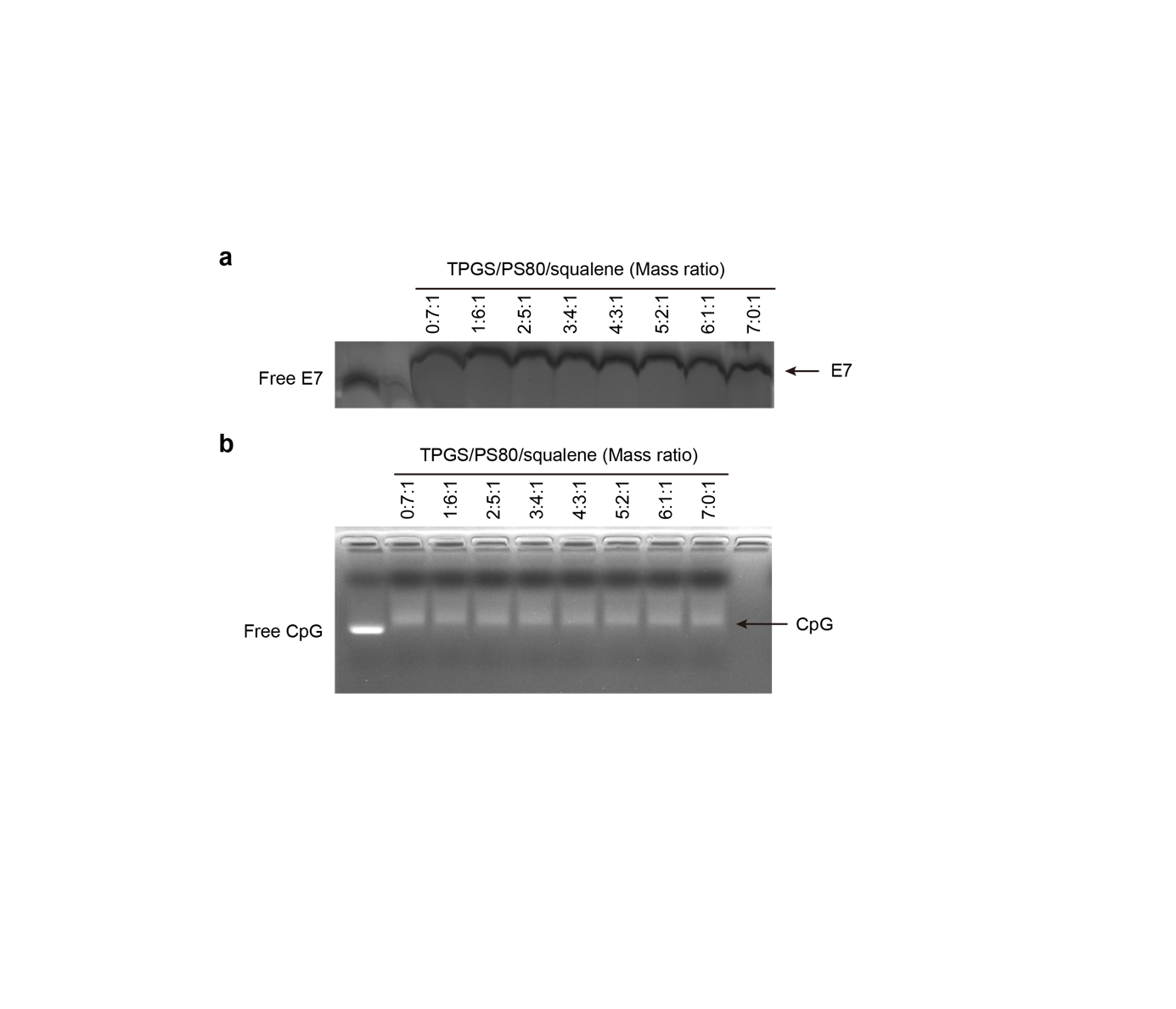


**Figure S10. Encapsulation efficacies of E7 (a) and CpG (b) for SSE-E7/CpG containing different ratios of TPGS, PS80, and squalene.**


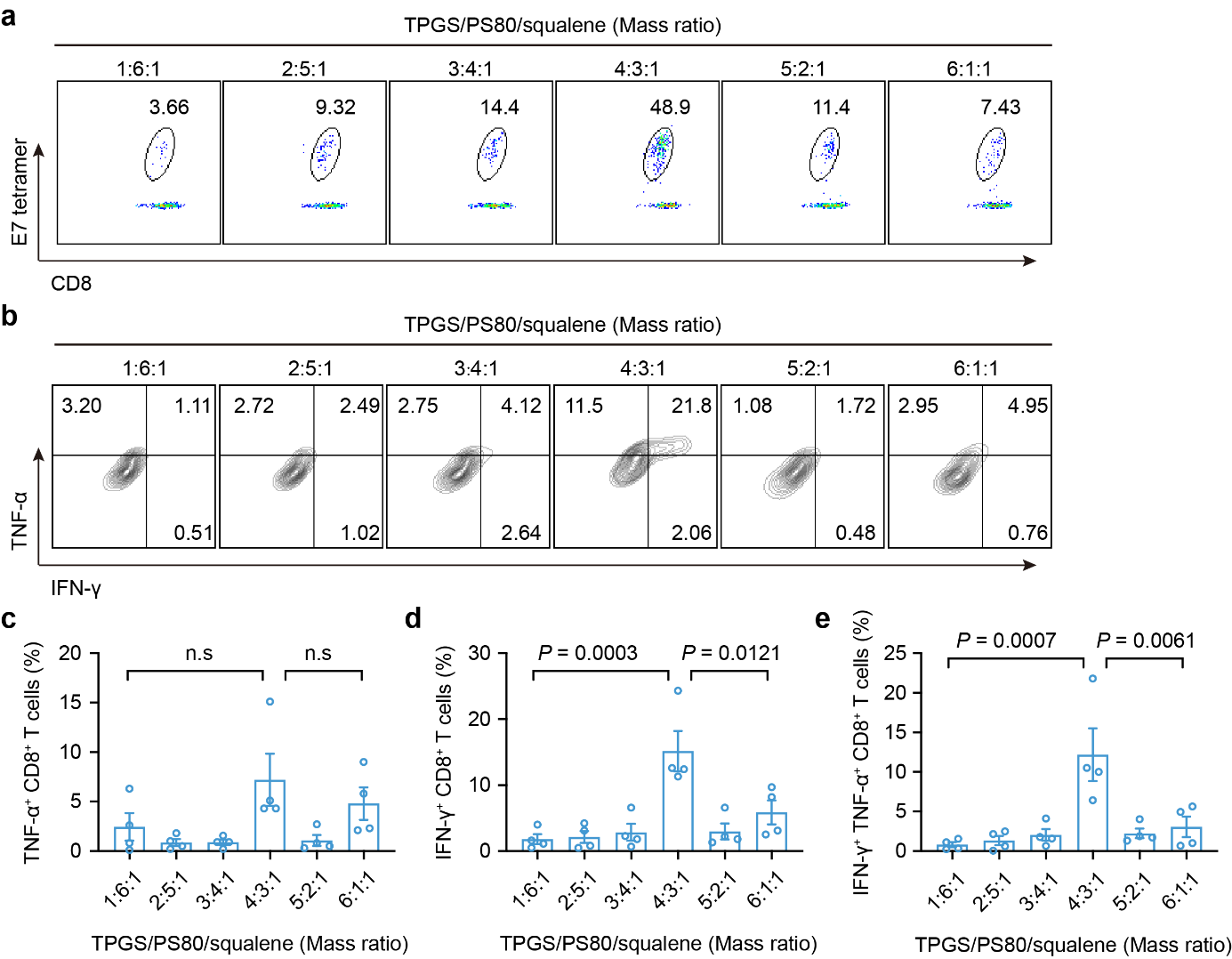


**Figure S11. Immune responses induced by various formulations of SSE-E7/CpG.** C57BL/6 mice were immunized with SSE-E7/CpG on days 0 and 7. On day 14, the antigen-specific T cells among PBMCs were analyzed by tetramer staining or intracellular cytokine staining. (n = 4 mice per group). a) Representative flow cytometry scatter plots for E7-specific CD8^+^ T cells among PBMCs on day 14 after immunization with SSE-E7/CpG. b-e) Representative flow cytometry scatter plots and quantitative analyses of cytokine-producing CD8^+^ T cells. Data represent mean ± SEM. Data were analyzed by one-way ANOVA with Tukey’s multiple comparisons test (c-e).


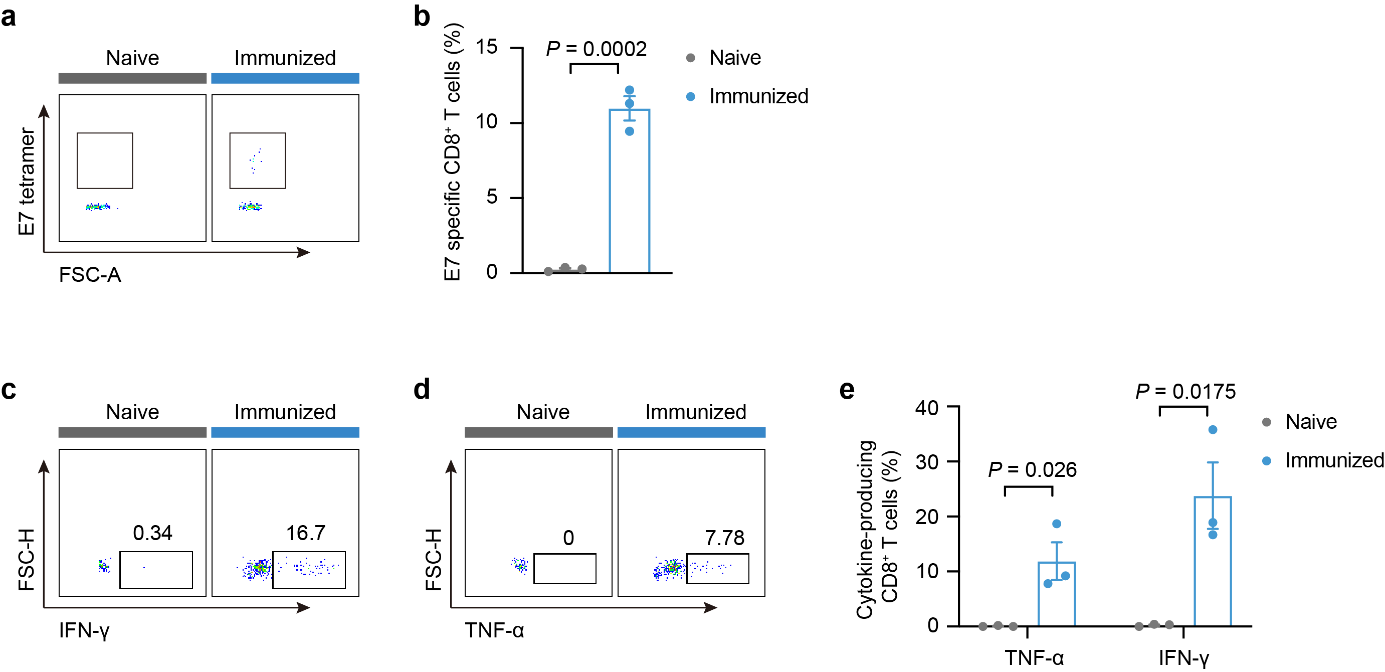


**Figure S12. Immune responses induced by a low dose of SSE-E7/CpG.** a, b) Representative flow cytometry scatter plots and quantitative analyses of E7-specific CD8^+^ T cells among PBMCs on day 14. c-e) Representative flow cytometry scatter plots and quantitative analyses of cytokine-producing CD8^+^ T cells among PBMCs on day 14. (n = 3 mice per group). Data represent mean ± SEM. Data were analyzed by a two-sided unpaired Student’s *t*-test (b, e).


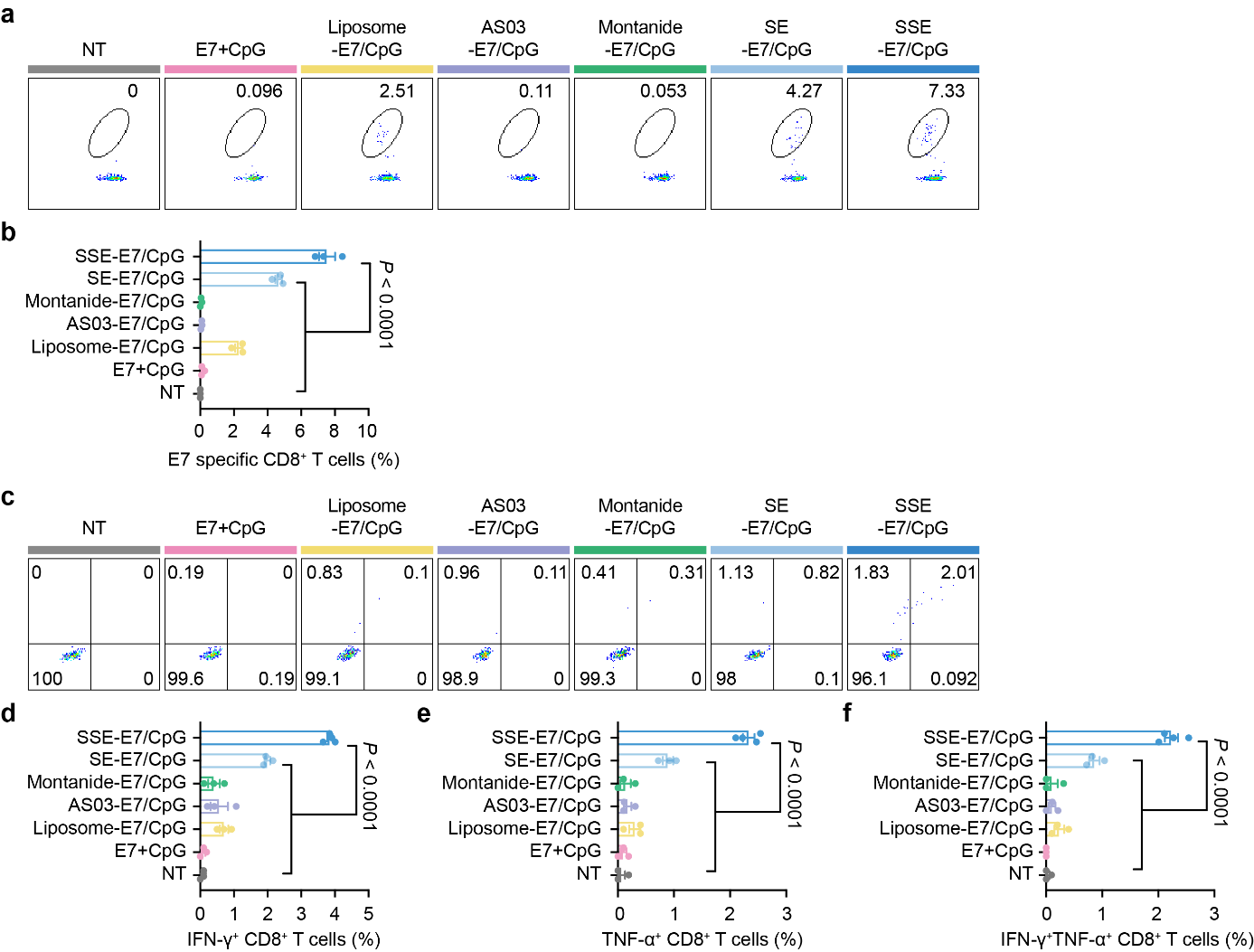


**Figure S13. Immune responses in the spleens of mice immunized with the indicated formulations.** C57BL/6 mice were immunized with SSE-E7/CpG on days 0 and 7. On day 14, the antigen-specific T cells in the spleen were analyzed by tetramer staining or intracellular cytokine staining. (n = 3 mice per group). a) Representative flow cytometry scatter plots, and (b) quantitative analyses of E7-specific CD8^+^ T cells in the spleen. c-f) Representative flow cytometry scatter plots and quantitative analyses of cytokine-producing CD8^+^ T cells in the spleen on day 14. Data represent mean ± SEM. Data were analyzed by one-way ANOVA with Tukey’s multiple comparisons test (b, d-f).


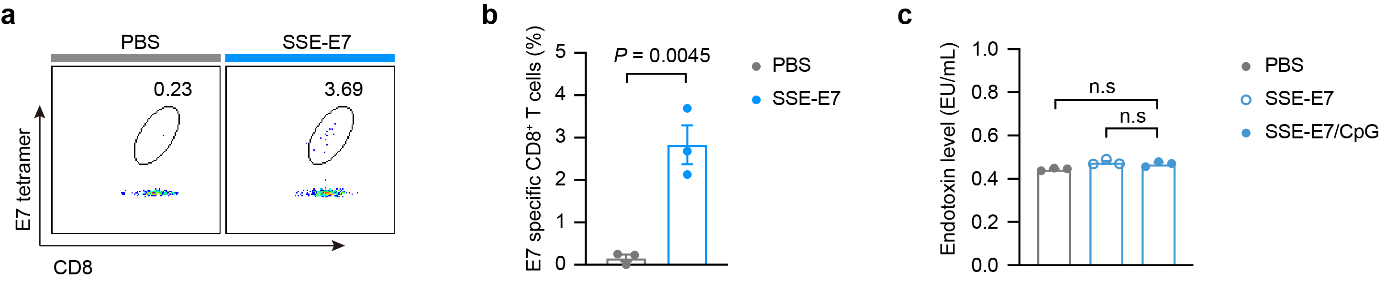


**Figure S14. Immunogenicity of SSE-E7.** C57BL/6 mice were immunized with SSE-E7 on days 0 and 7. On day 14, the antigen-specific T cells among PBMCs were analyzed by tetramer staining. (n = 3 experimental replicates per group). a) Representative flow cytometry scatter plots, and b) quantitative analyses of E7-specific CD8^+^ T cells among PBMCs on day 14. c) Endotoxin levels of SSE-E7 and SSE-E7/CpG. Data represent mean ± SEM. Data were analyzed by two-sided unpaired Student’s *t*-test (b) or one-way ANOVA with Tukey’s multiple comparisons test (c).


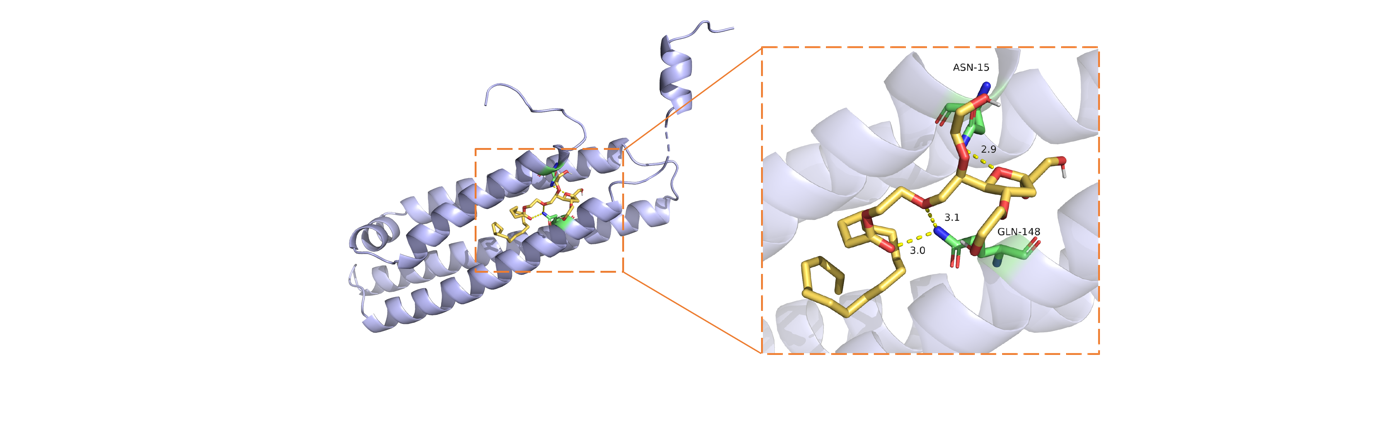


**Figure S15. Molecule docking representations of polysorbate 80 binding to ApoE.** Polysorbate 80 and ApoE are depicted in purple and color, respectively. A magnified view of the interaction of ApoE with polysorbate 80 is shown in the dashed box.


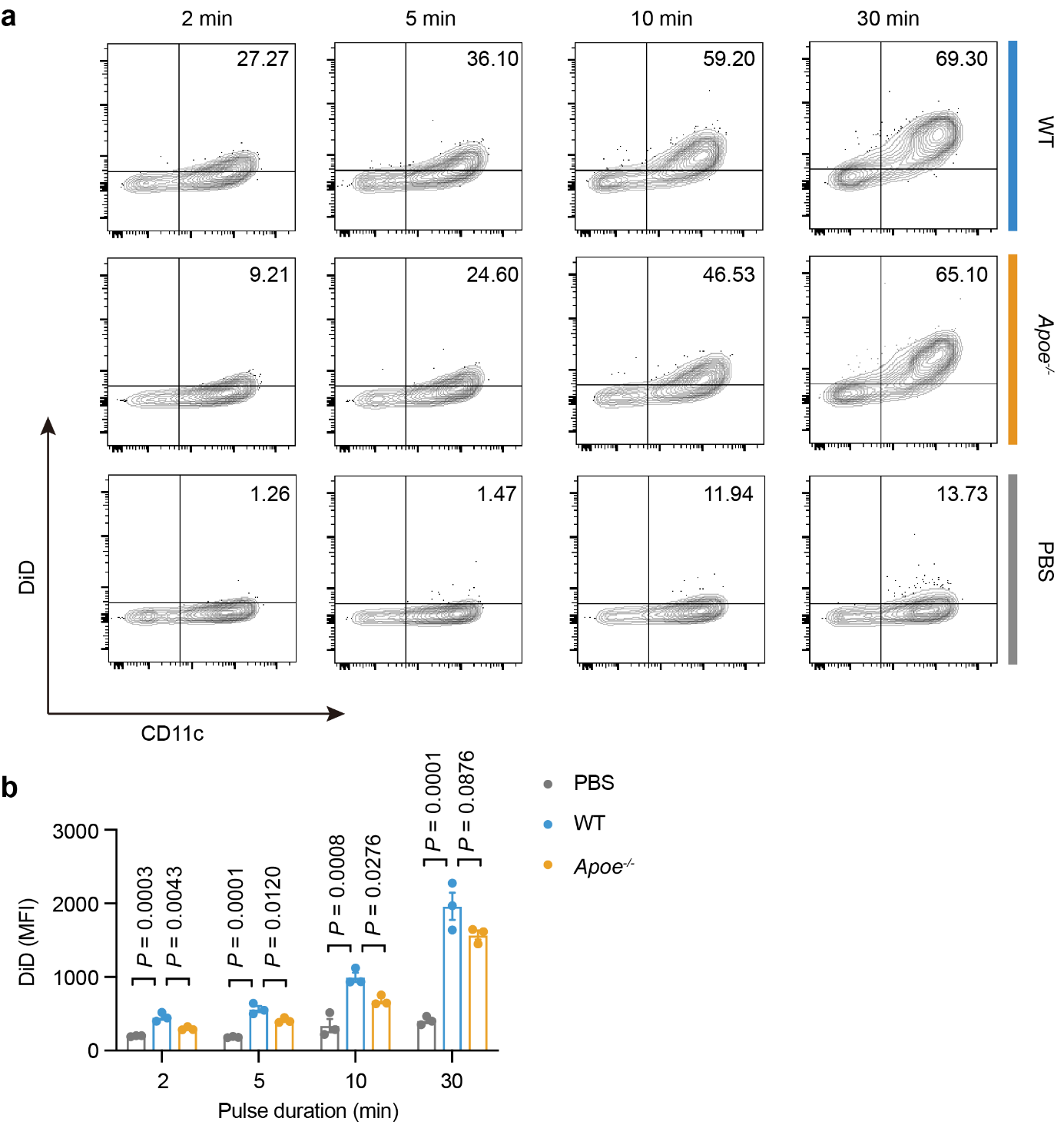


**Figure S16. SSE-DiD uptake by BMDCs after incubation with serum from WT or *Apoe^-/-^* mice.** a) Representative flow cytometry scatter plots, and b) quantitative analyses of SSE-DiD uptake by BMDCs (n = 3 experimental replicates per group). Data represent mean ± SEM. Data were analyzed by two-way ANOVA with Dunnett’s multiple comparisons test (b).


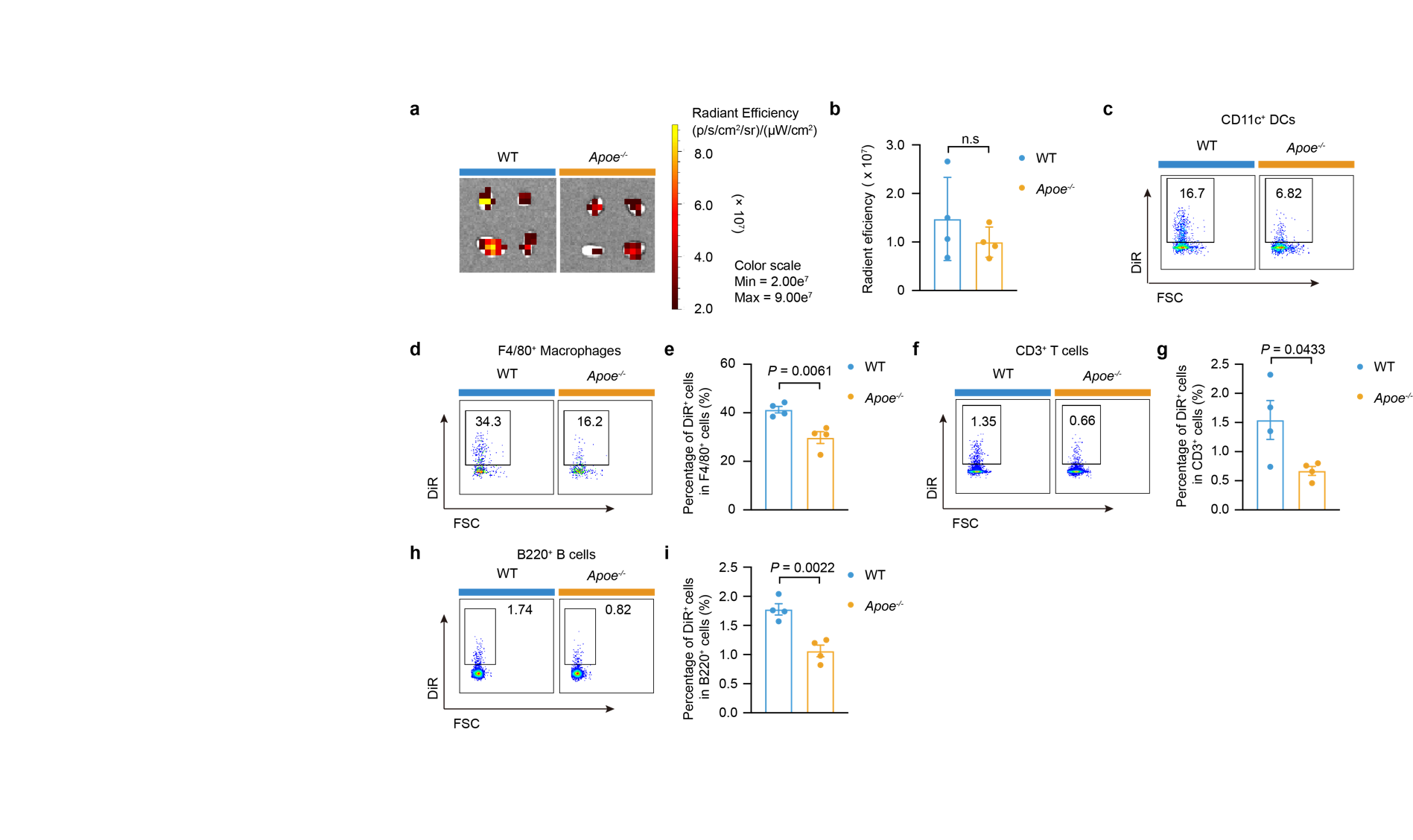


**Figure S17. SSE-DiR accumulation in inguinal lymph nodes of *Apoe^-/-^* mice.** a) The accumulation of SSE-DiR in the inguinal lymph nodes of WT and *Apoe^-/-^* mice 2 hours after injection. b) Quantification of fluorescence signals in (a). (n = 4 experimental replicates per group). c) Representative flow cytometry scatter plots of SSE-DiR uptake by CD11c^+^ DCs. d-i) Representative flow cytometry scatter plots and quantitative analyses of SSE-DiR uptake by F4/80^+^ macrophages (d, e), CD3^+^ T cells (f, g), and B220^+^ B cells (h, i). (n = 4 experimental replicates per group). Data represent mean ± SEM. Data were analyzed by two-sided unpaired Student’s *t*-test (b, e, g, i).


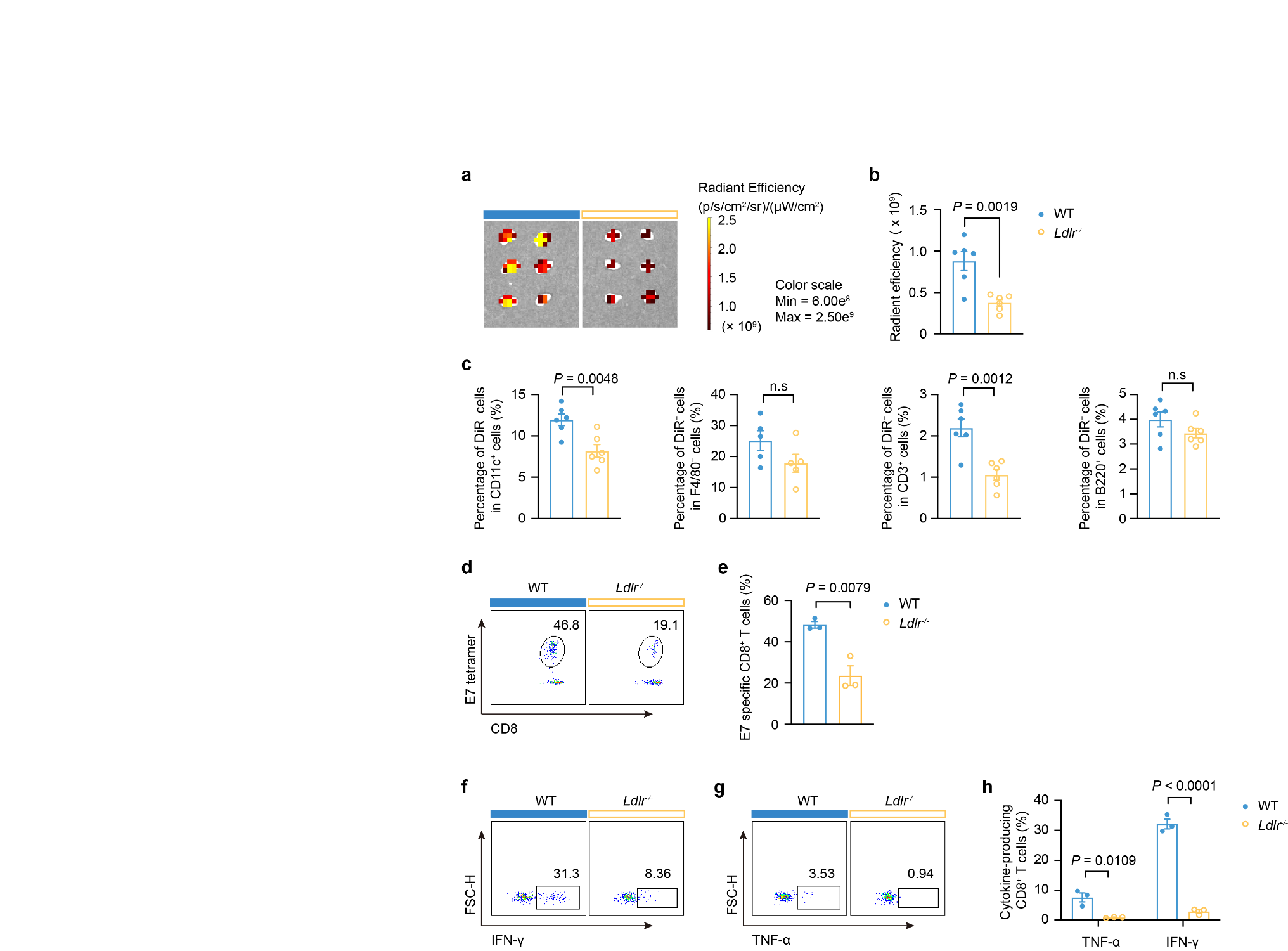


**Figure S18. Effect of knocking out LDLR on the delivery profile and immune responses of SSE-based vaccine.** a) The accumulation of SSE-DiR in the inguinal lymph nodes of WT and *Ldlr^-/-^* mice 24 hours after injection. b) Quantification of fluorescence signals in (a). (n = 6 experimental replicates per group). c) Quantitative analyses of SSE-DiR uptake by CD11c^+^ DCs, F4/80^+^ macrophages, CD3^+^ T cells, and B220^+^ B cells. (n = 6 experimental replicates per group). d-h) WT or *Ldlr^-/-^* mice were vaccinated with SSE-E7/CpG on days 0 and 7, and T-cell responses were analyzed on day 14. (d, e) Representative flow cytometry scatter plots and quantitative analyses of E7-specific CD8^+^ T cells among PBMCs on day 14. (f, g) Representative flow cytometry scatter plots and (h) quantitative analyses of cytokine-producing CD8^+^ T cells among PBMCs on day 14. (n = 3 mice per group). Data represent mean ± SEM. Data were analyzed by two-sided unpaired Student’s *t*-test (b, c, e, h).


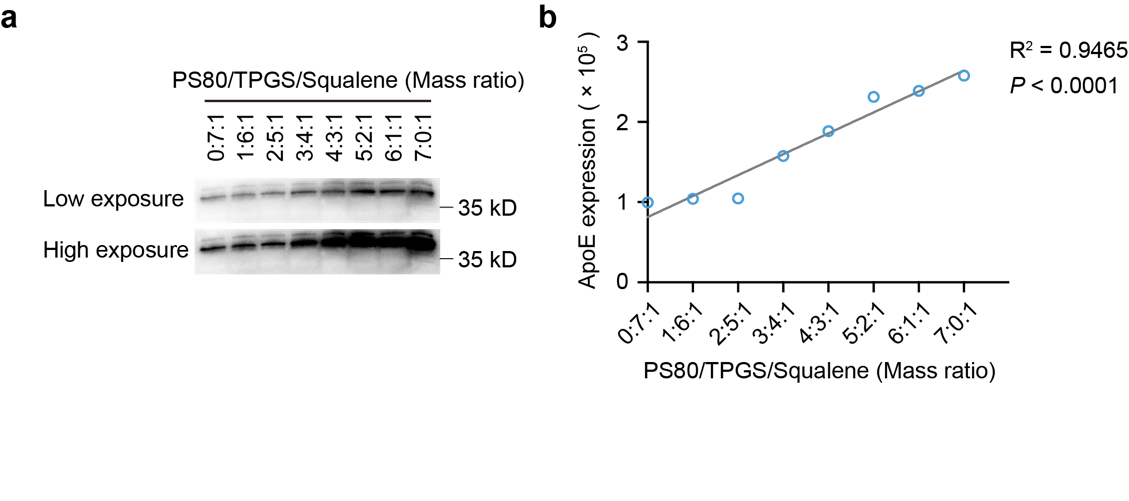


**Figure S19. ApoE adsorbed on SSE relies on PS80.** a) Western blot of ApoE adsorbed on the indicated SSE groups. b) The correlation analyses of ApoE expression and PS80.


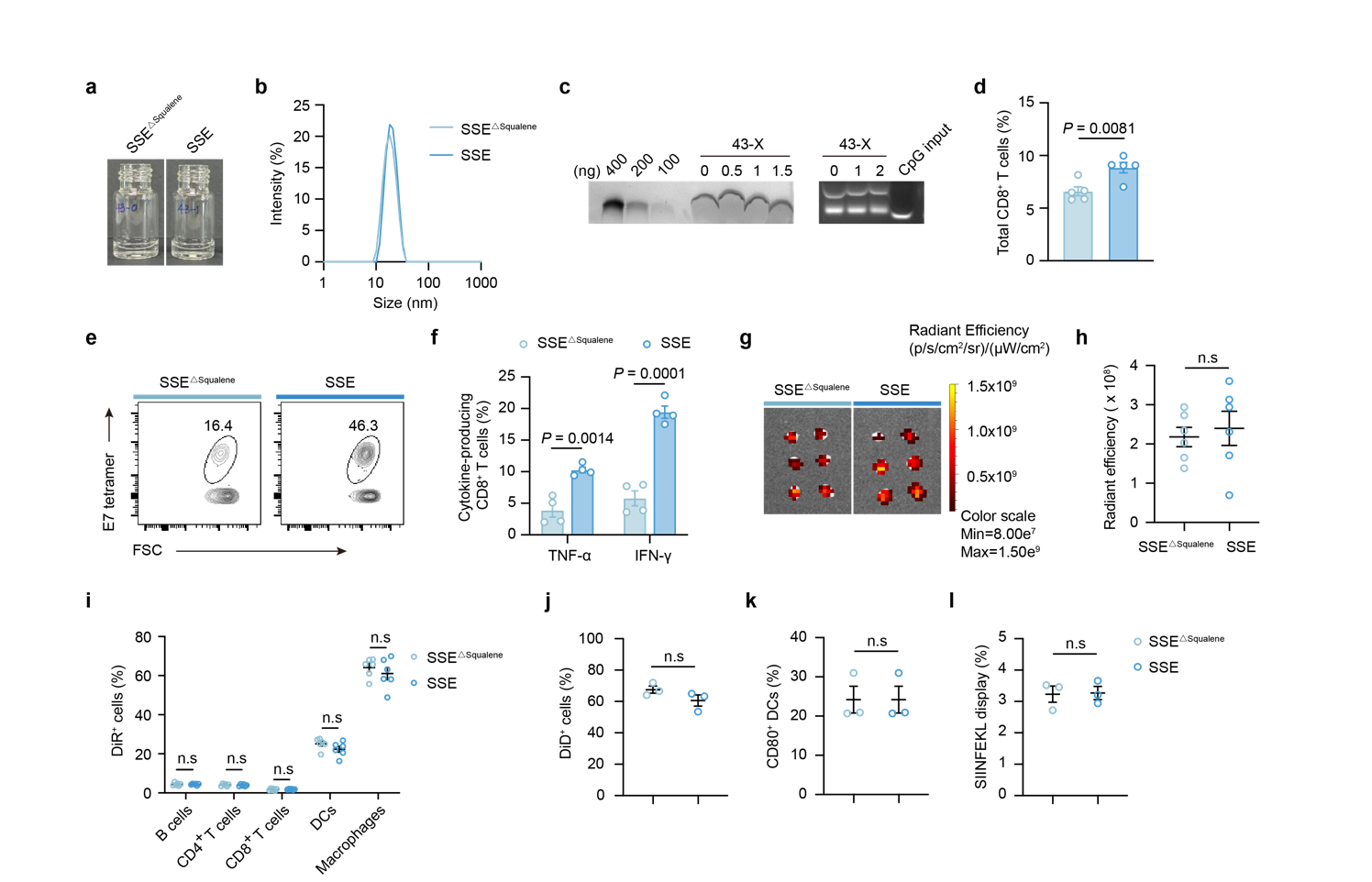


**Figure S20. T-cell activation promoted by squalene is independent of drainage efficiency or APC activation.** a) The image of SSE and SSE^△Squalene^. b) Size distributions of SSE and SSE^△Squalene^. c) Quantifications of E7 and CpG. d-f) Mice were subcutaneously injected with the SSE or SSE^△Squalene^ vaccine on days 0 and 7, and the immune responses were analyzed on day 14. d) quantitative analyses of total CD8^+^ T cells among PBMCs. (n = 5 mice per group). Shown are (e) representative flow cytometry scatter plots of E7-specific CD8^+^ T cells. f) Quantification of cytokine-producing CD8^+^ T cells. (n = 4 mice per group). g, h) Mice were subcutaneously injected with SSE-DiR or SSE^△Squalene^-DiR, and the lymph nodes were harvested at 24 h post-injection for analysis. Shown are (g) the accumulation of SSE-DiR or SSE^△Squalene^-DiR in mice analyzed by the IVIS imaging system, and h, the quantification of the fluorescence signals in (g). i) the uptake of SSE-DiR or SSE^△Squalene^-DiR by immune cells in the inguinal lymph nodes. (n = 6 experimental replicates per group). j) Uptake of SSE-DiD or SSE^△Squalene^-DiD by BMDCs. Mice were subcutaneously injected with the SSE vaccine or the SSE^△Squalene^ vaccine, and the lymph nodes were harvested at 24 h post-injection for analysis. Shown are k) the expression of CD80 on CD11c^+^ DCs, and l) antigen presentation on CD11c^+^ DCs. (n = 3 experimental replicates per group). Data represent mean ± SEM. Data were analyzed by two-sided unpaired Student’s *t*-test.


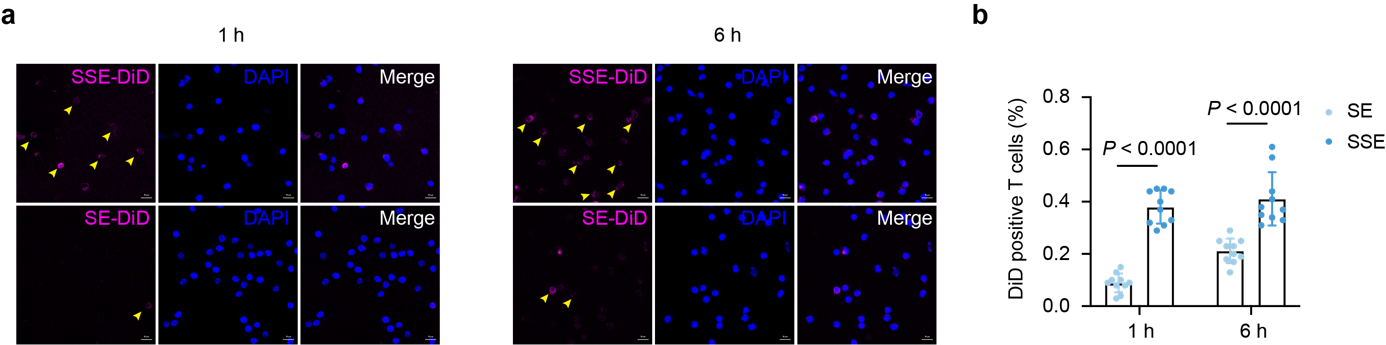


**Figure S21. SE and SSE internalization by CD8^+^ T cells.** a) Confocal images of CD8^+^ T cells treated with SSE-DiD or SE-DiD for the indicated lengths of time. Scale bars, 10 μm. Yellow arrows indicate internalization of formulations by T cells. b) DiD-positive T cells were quantified (n = 10 fields of view in total). Data represent mean ± SEM. Data were analyzed by two-way ANOVA with Sidak’s multiple comparisons test.


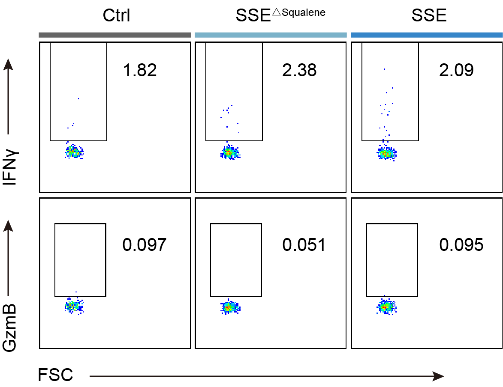


**Figure S22.** Cytokine and cytolytic granule production of naïve CD8^+^ T cells pretreated with SSE or SSE^△Squalene^.


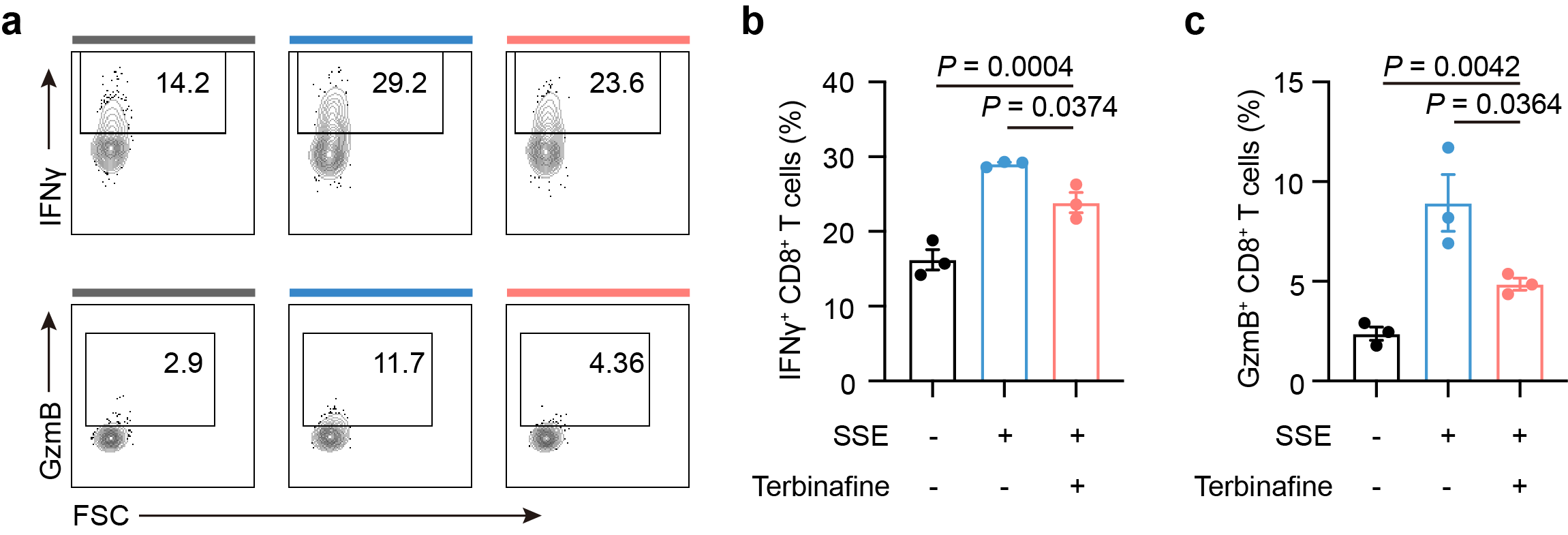


**Figure S23****. Cytokine and cytolytic granule production of CD8^+^ T cells stimulated with 2 μg ml^-1^ anti-CD3/CD28.** a) Representative flow cytometry scatter plots of cytokine- and cytolytic granule-producing CD8^+^ T cells. b and c) Quantitative analyses of cytokine- and cytolytic granule-producing CD8^+^ T cells (n = 3 experimental replicates per group). Data represent mean ± SEM. Data were analyzed by one-way ANOVA with Tukey’s multiple comparisons test (b, c).


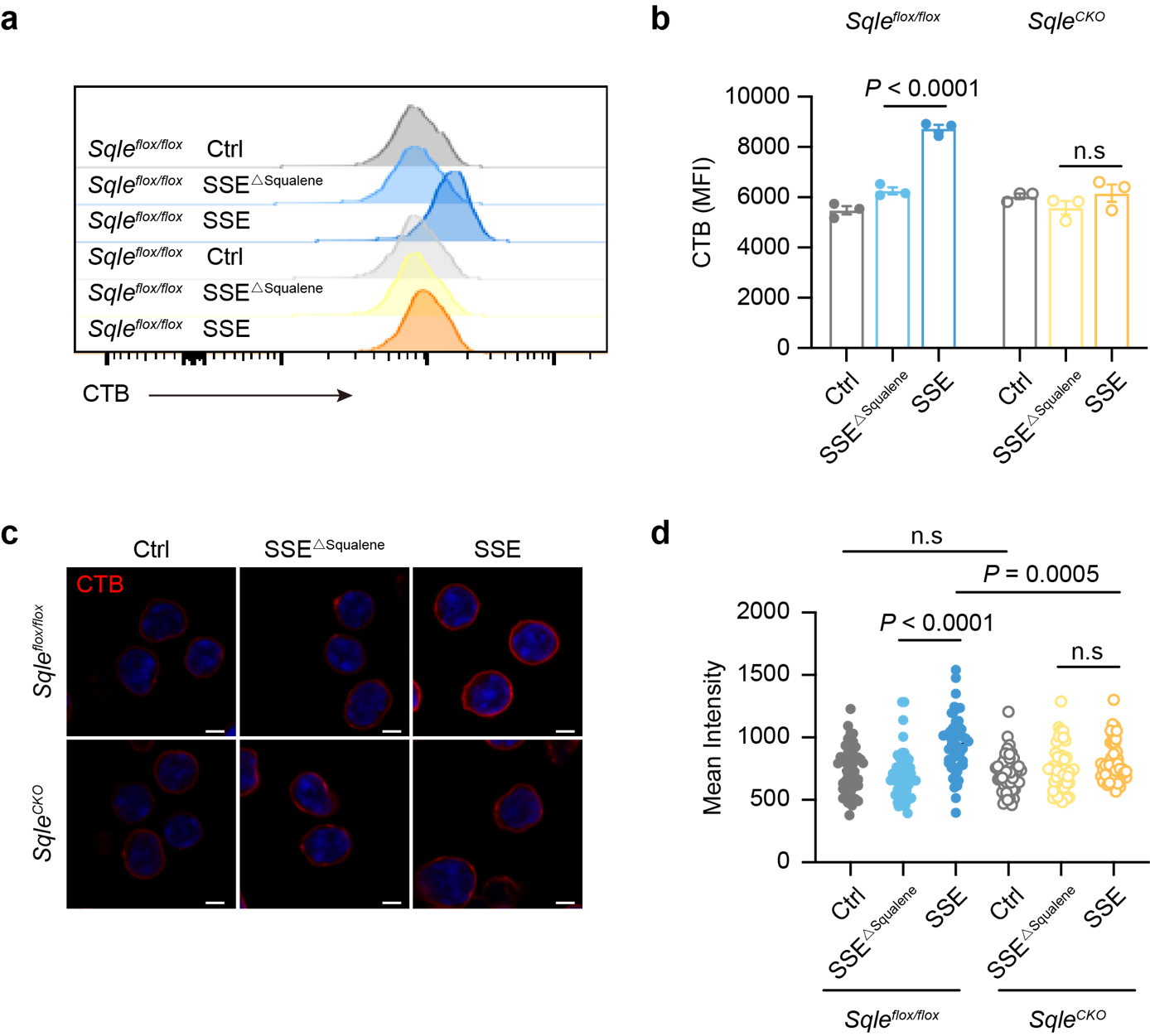


**Figure S24. Membrane lipid raft on CD8^+^ T cells by CTB staining.** a, b) Representative flow cytometry scatter plots and quantitative analyses of CTB on CD8^+^ T cells from *Sqle^flox/flox^* and *Sqle^CKO^* mice by flow cytometry. (n = 3 experimental replicates per group). c) Immunofluorescence staining for CTB on the cell membrane. Scale bars, 5 μm. d) Fluorescence quantification of CTB staining in (c) (n = 50 cells). Data represent mean ± SEM. Data were analyzed by two-way ANOVA with Tukey’s multiple comparisons test (b) and one-way ANOVA with Tukey’s multiple comparisons test (d).


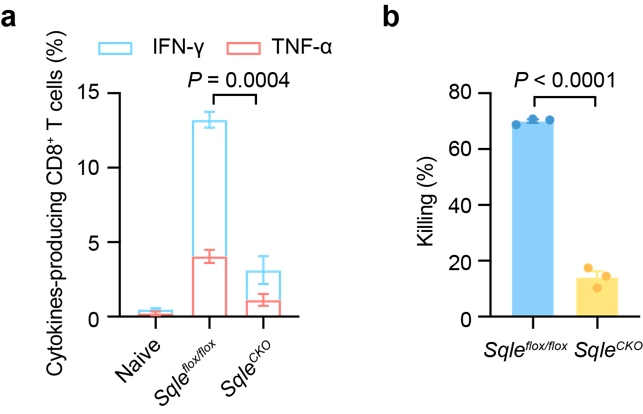


**Figure S25. Immune responses in *Sqle^CKO^* mice after SSE vaccine injection.** Mice were immunized with the SSE vaccine on days 0 and 7, and T-cell responses were analyzed on day 14. a) Cytokine-producing CD8^+^ T-cells from *Sqle^flox/flox^* and *Sqle^CKO^* mice. (n = 3 mice per group). b) Quantitative analyses of E7-specific cytotoxicity *in vivo*. (n = 3 mice per group). Data represent mean ± SEM. Data were analyzed by one-way ANOVA with Tukey’s multiple comparisons test (a) and two-sided unpaired Student’s *t*-test (b).

.


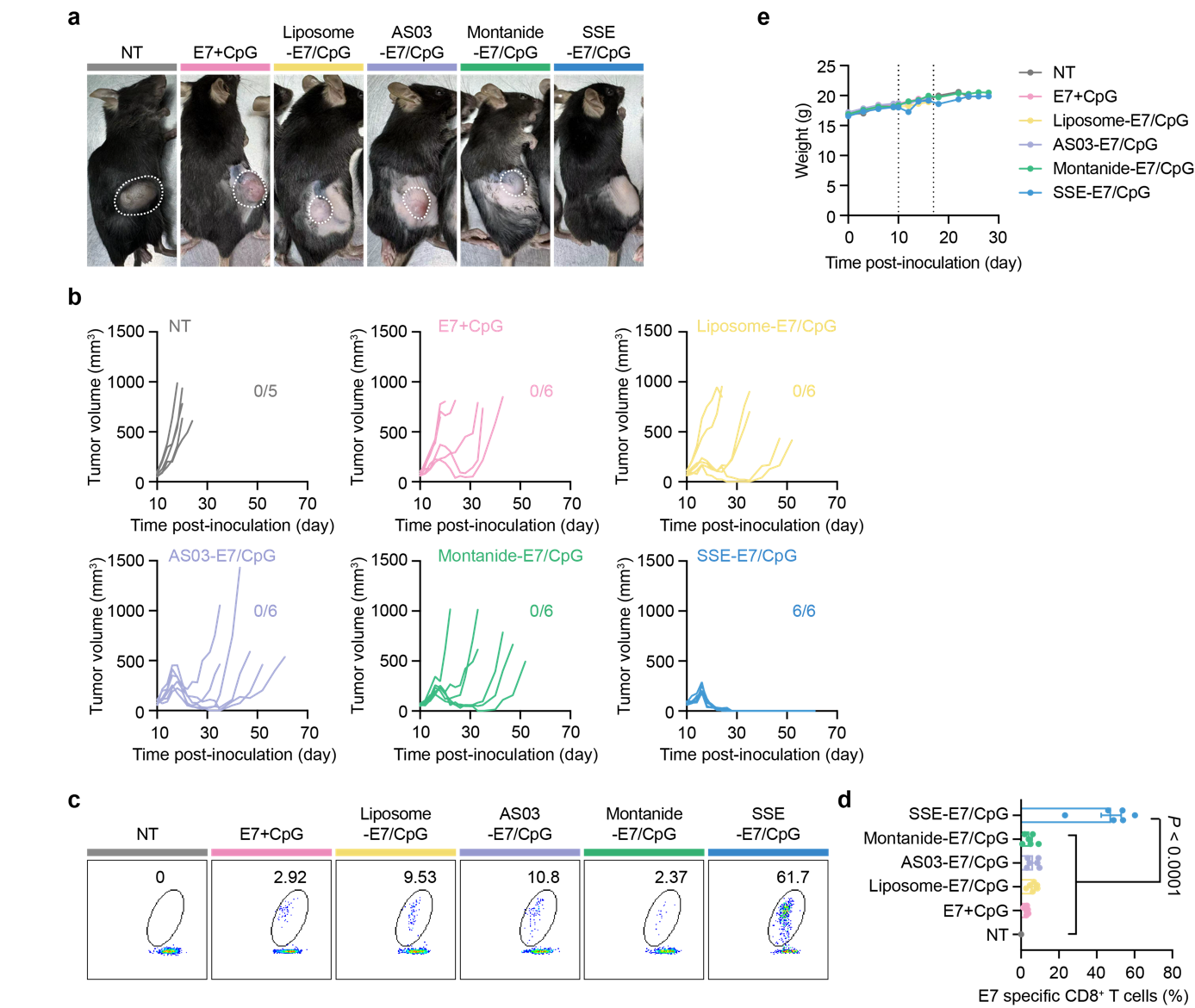


**Figure S26. Therapeutic effect of indicated formulations on the subcutaneous TC-1 tumor model.** C57BL/7 mice were inoculated with TC-1 cells on day 0. On days 10 and 17, animals were subcutaneously vaccinated with the indicated formulations. a) The representative tumor images on day 26. b) Individual tumor growth in C57BL/6 mice treated with the indicated formulations. c) Representative flow cytometry scatter plots, and d) quantitative analyses of E7-specific CD8^+^ T cells among PBMCs from tumor-bearing mice treated with indicated formulations on day 24. (n = 1 – 6 mice per group). e) Body weight changes of mice after treatment with different formulations. Data represent mean ± SEM. Data were analyzed by one-way ANOVA with Tukey’s multiple comparisons test (d).


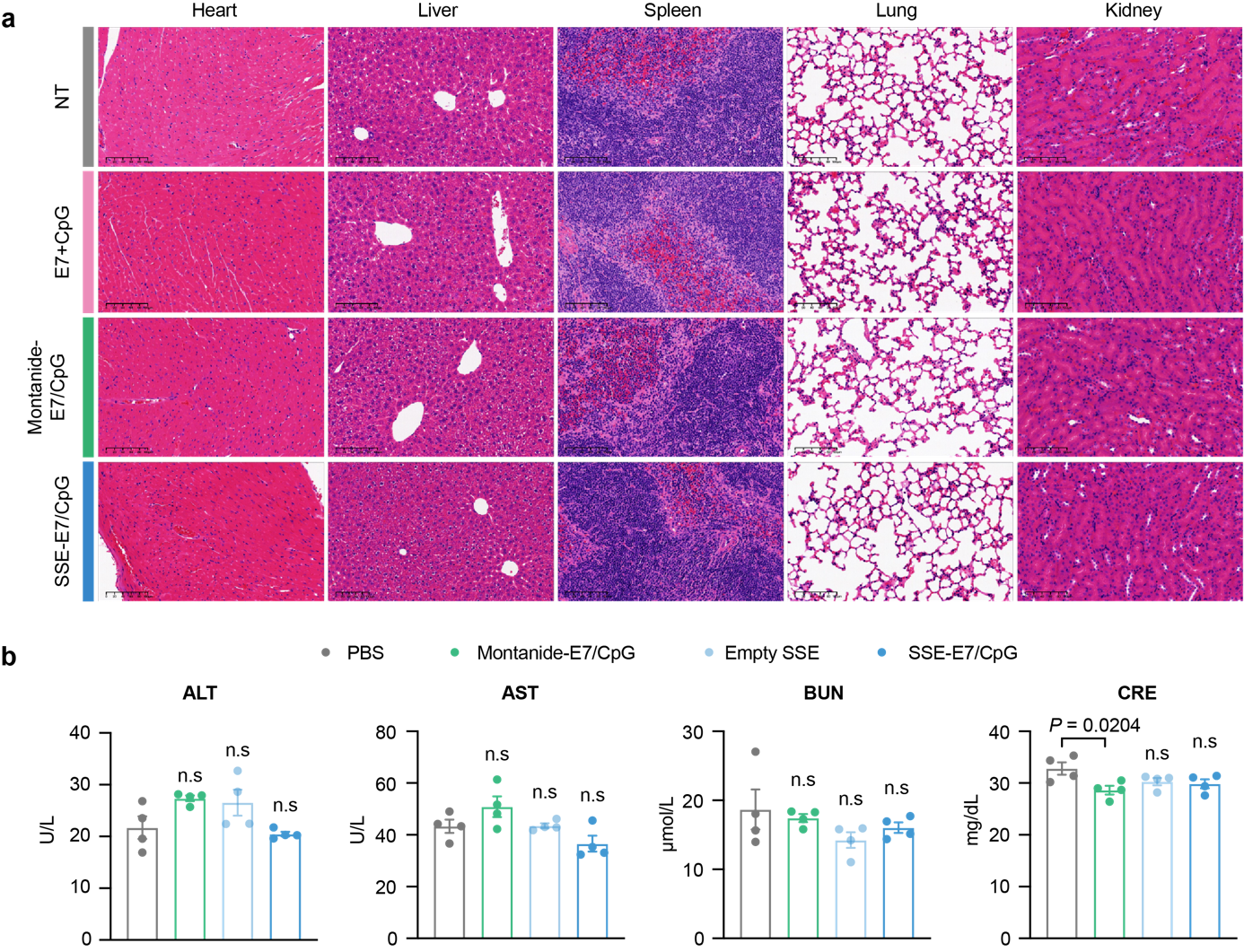


**Figure S27. Safety profiles of SSE vaccines.** a) TC-1 tumor-bearing mice were vaccinated on days 10 and 17. Major organs were harvested on day 28 for histological analyses. Scale bars, 100 μm. b) Naive mice were immunized with the indicated formulations, and blood was collected on day 4 for biochemical analysis. (n = 4 experimental replicates per group). Data represent mean ± SEM. Data were analyzed by one-way ANOVA with Tukey’s multiple comparisons test (b).


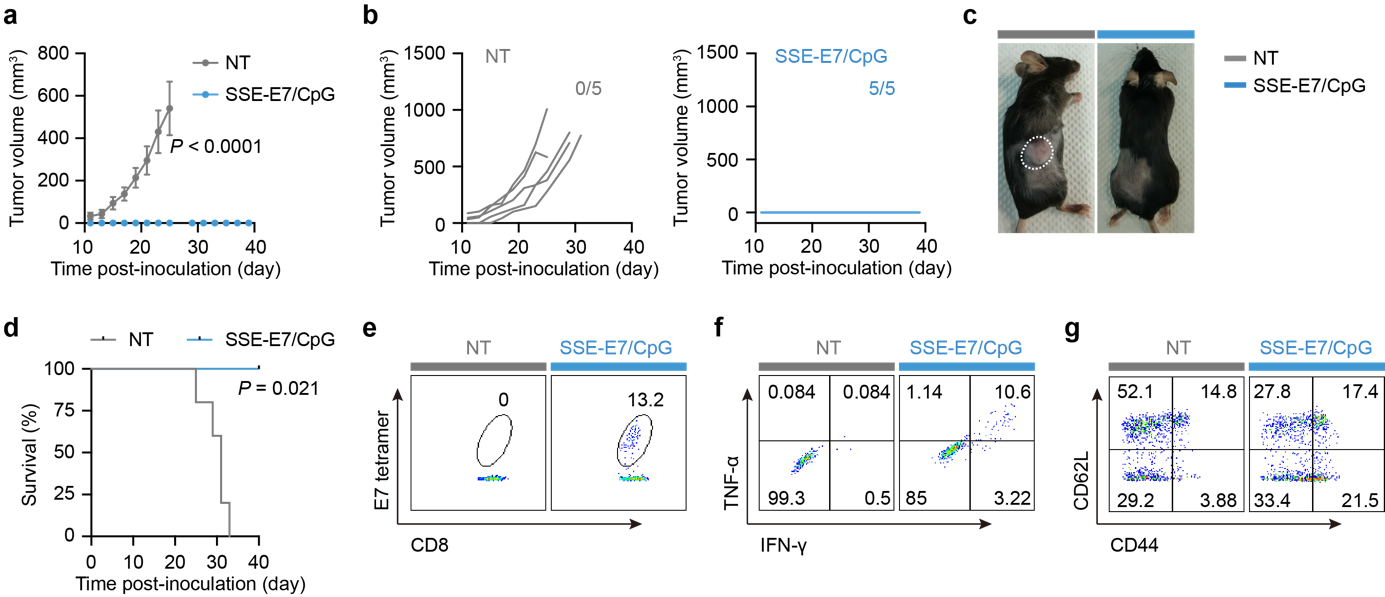


**Figure S28. Immune responses in mice rechallenged with TC-1 tumors.** Cured animals were rechallenged with TC-1 tumors on the contralateral side on day 150. (n = 5 mice per group). a) Average tumor growth curves in mice. b) Individual tumor growth curve. c) The images of mice rechallenged with TC-1 tumor cells on day 25 after rechallenge with TC-1 cells. d) Survival curves of mice rechallenged with TC-1 tumor cells. Representative flow cytometry scatter plots of E7-specific CD8^+^ T cells (e) and cytokine-producing CD8^+^ T cells (f) among PBMCs on day 25 after TC-1 rechallenge. g, Representative flow cytometry scatter plots of CD8^+^ T memory cells. Data represent mean ± SEM. Data were analyzed by two-way ANOVA with Dunnett’s multiple comparisons test (a), the log-rank (Mantel-Cox) test (d).


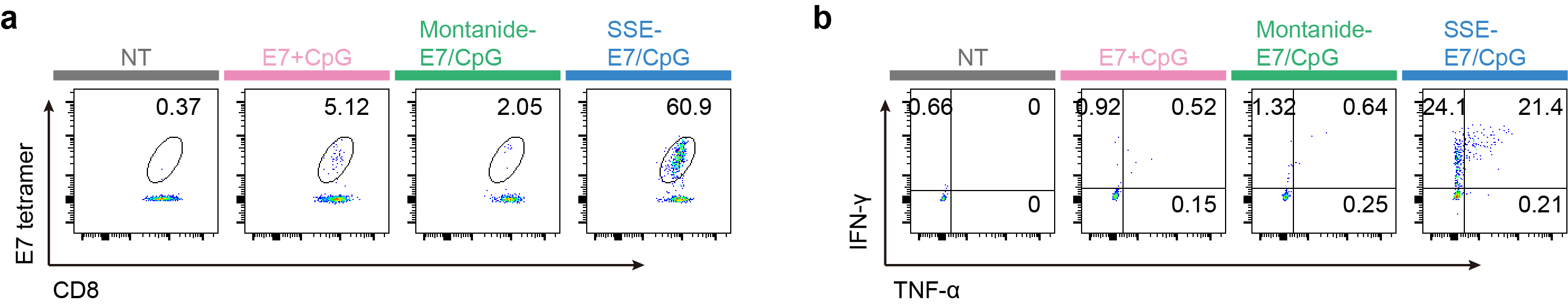


**Figure S29. Therapeutic effect of indicated formulations on the orthotopic TC-1 model.** Mice were immunized with the indicated formulations on days 10 and 17. Representative flow cytometry scatter plots of antigen-specific (a) and cytokine-producing (b) CD8^+^ T cells among PBMCs from mice on day 24.


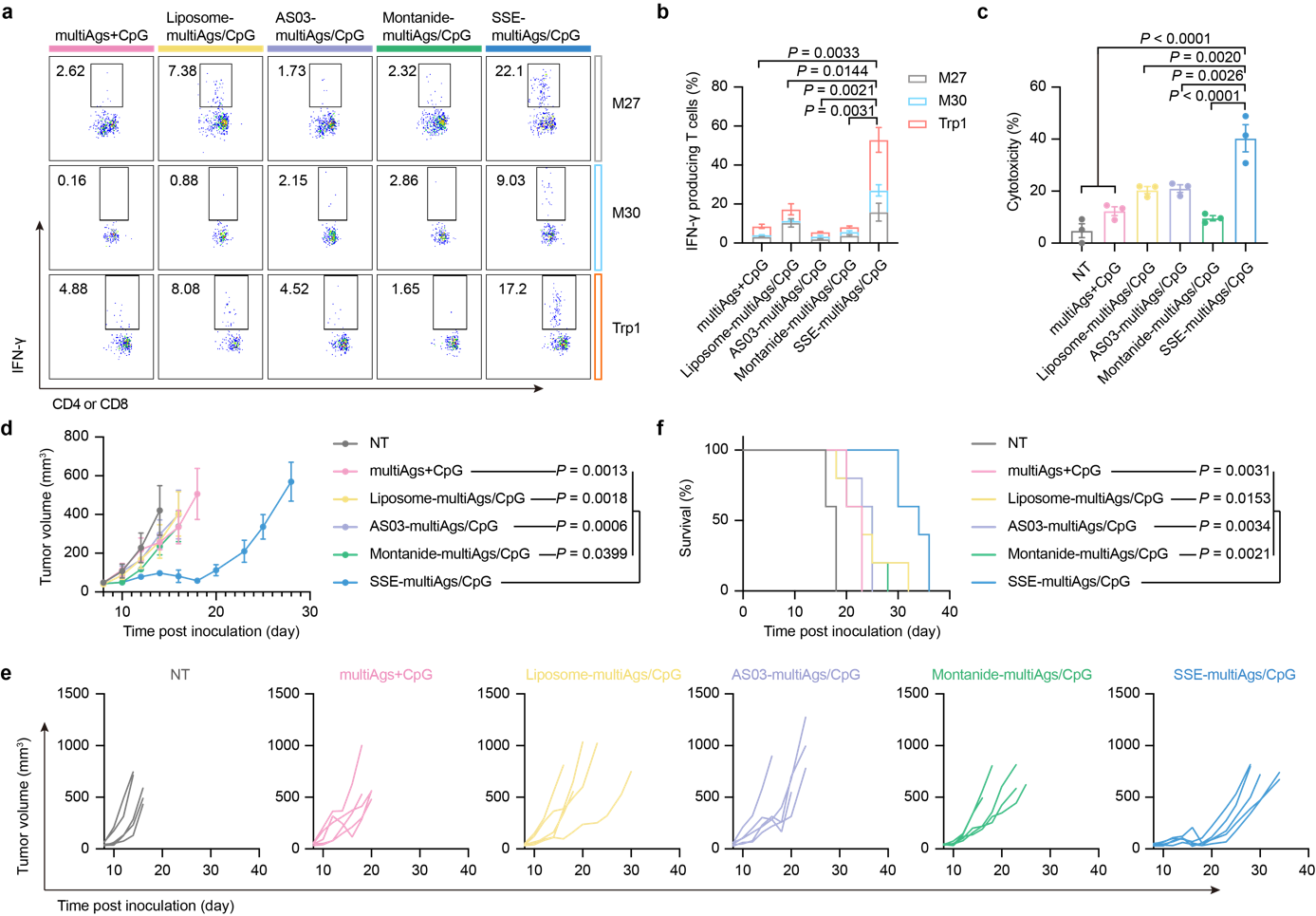


**Figure S30. SSE vaccines induce potent therapeutic effects on B16F10 tumors.** a, b) C57BL/6 mice were treated with the indicated vaccines on days 0 and 7. On day 14, antigen-specific T-cell responses were analyzed using an intracellular cytokine staining assay. Shown are (a) representative flow cytometry scatter plots of IFN-γ-producing T cells among PBMCs, and (b) quantitative analyses of IFN-γ-producing T cells among PBMCs from mice immunized with indicated formulations (n = 3 experimental replicates per group). c) On day 14, splenocytes from mice immunized with indicated formulations were incubated with B16F10 cells for 8 h, and LDH release was measured to assess cytotoxic efficacy (n = 3 experimental replicates per group). d-f) C57BL/6 mice were inoculated with 1 × 10^5^ B16F10 cells on day 0. On days 8 and 15, animals were treated with the indicated formulations. Shown are (d) average tumor growth curves, (e) individual tumor growth in mice, and (f) the survival curves of tumor-bearing mice vaccinated with the indicated preparations (n = 5 mice per group). Data represent mean ± SEM. Data were analyzed by one-way ANOVA with Tukey’s multiple comparisons test (b, c), two-way ANOVA with Dunnett’s multiple comparisons test (d), or the log-rank (Mantel-Cox) test (f).

Table S1 Characteristics of the SSE vaccine and the control groups

| Formulations | | Size (nm) | PDI | Zeta (mV) |
| --- | --- | --- | --- | --- |
| Mass ratio of  (TPGS/PS80/Squalene) | 0:7:1 | 16.78 ± 1.74 | 0.25 ± 0.08 | - |
|  | 1:6:1 | 15.99 ± 1.47 | 0.24 ± 0.06 | - |
|  | 2:5:1 | 16.61 ± 1.58 | 0.24 ± 0.06 | - |
|  | 3:4:1 | 15.84 ± 0.44 | 0.25 ± 0.03 | - |
|  | 4:3:1 | 17.64 ± 1.28 | 0.11 ± 0.04 | - |
|  | 5:2:1 | 18.34 ± 2.12 | 0.21 ± 0.09 | - |
|  | 6:1:1 | 19.24 ± 1.53 | 0.27 ± 0.06 | - |
|  | 7:0:1 | 20.32 ± 1.53 | 0.26 ± 0.06 | - |
| TPGS/PS80/Squalene = 4:3:1 | Empty SE | 167.2 ± 22.82 | 0.16 ± 0.07 | -4.68 ± 0.31 |
|  | Empty SSE | 18.47 ± 0.71 | 0.15 ± 0.01 | -2.46 ± 0.22 |
|  | SE-E7/CpG | 187.07 ± 3.03 | 0.14 ± 0.02 | -9.23 ± 0.39 |
|  | SSE-E7/CpG | 18.49 ± 0.43 | 0.17 ± 0.04 | -8.60 ± 0.75 |
|  | Concentrated SSE-E7/CpG | 20.28 ± 1.26 | 0.25 ± 0.06 | -6.96 ± 1.48 |
| DOPC-E7/CpG | | 140.2 ± 25.82 | 0.23 ± 0.03 | - |
| AS03 + E7 + CpG | | 178.2 ± 3.45 | 0.13 ± 0.02 | - |

Table S2. Antigen and cho-CpG loading efficacies of the formulations

| Loading efficacies (%) | DOPC liposomes | SE vaccines | SSE vaccines | SSE vaccines after lyophilization |
| --- | --- | --- | --- | --- |
| SIINFEKL | 82.10 ± 5.25 | 81.56 ± 2.00 | 55.93 ± 1.41 | - |
| E7 | 89.35 ± 11.43 | 89.54 ± 11.51 | 63.85 ± 4.55 | 58.46 ± 3.12 |
| M27 | 96.48 ± 2.05 | - | 59.19 ± 1.33 | - |
| M30 | 95.50 ± 3.39 | - | 53.37 ± 11.41 | - |
| Trp1 | 82.98 ± 5.40 | - | 36.26 ± 7.94 | - |
| CpG | 95.52 ± 4.02 | 18.57 ± 6.77 | 18.66 ± 9.41 | 19.64 ± 1.61 |

Table S3. Key resources

| **REAGENT** | **SOURCE** | **IDENTIFIER** |
| --- | --- | --- |
| **Antibodies** |  |  |
| Rat monoclonal anti-mCD3-FITC (17A2) | BioLegend | Cat#100204 |
| Rat monoclonal anti-mCD4-FITC (RM4-4) | BioLegend | Cat#116003 |
| Rat monoclonal anti-mCD25-FITC (PC61) | BioLegend | Cat#102005 |
| Rat monoclonal anti-mF4/80-FITC (BM8) | BioLegend | Cat#123107 |
| Rat monoclonal anti-mCD44-FITC (IM7) | BioLegend | Cat#103005 |
| Rat monoclonal anti-mTNFα-FITC (MP6-XT22) | BioLegend | Cat#506304 |
| Rat monoclonal anti-mB220-APC (RA3-6B2) | BioLegend | Cat#103212 |
| Rat monoclonal anti-mCD8α-APC (53-6.7) | BioLegend | Cat#100712 |
| Rat monoclonal anti-mCD4-APC (GK1.5) | BioLegend | Cat#100411 |
| Rat monoclonal anti-mCD80-APC (16-10A1) | BioLegend | Cat#104713 |
| Rat monoclonal anti-mCD11c-PE (N418) | BioLegend | Cat#117308 |
| Rat monoclonal anti-mIFN-γ-PE (XMG1.2) | BioLegend | Cat#505808 |
| Rat monoclonal anti-mCD62L-PE (MEL-14) | BioLegend | Cat#104407 |
| Rat monoclonal anti-mCD86-PE-Cy7 (GL-1) | BioLegend | Cat#105014 |
| Rat monoclonal anti-mCD4-APC-Cy7 (GK1.5) | BioLegend | Cat#100414 |
| Rat monoclonal anti-mCD44-APC-Cy7 (IM7) | BioLegend | Cat#103027 |
| Rat monoclonal anti-mF4/80-BV605 (BM8) | BioLegend | Cat#123133 |
| Rat monoclonal anti-mCD69-BV605 (H1.2F3) | BioLegend | Cat#104530 |
| Rat monoclonal anti-mCD11c-Pacific Blue (N418) | BioLegend | Cat#117322 |
| Rat monoclonal anti-CD16/32 (93) | BioLegend | Cat#101302 |
| OVA_257-264_ (SIINFEKL) peptide bound to H-2Kb monoclonal antibody-APC (25-D1.16) | eBioscience | Cat#17-5743-82 |
| Rabbit monoclonal anti-Apolipoprotein E | Abcam | Cat#ab183596 |
| Goat anti-rabbit IgG H&L (HRP) | Zenbio | Cat#511203 |
| T-select H-2Kb OVA tetramer-SIINFEKL-PE | MBL | TS-5001-1C |
| T-select H-2Kb HPV16 E7 tetramer-RAHYNIVTF-PE | MBL | TB-5008-1 |
| **Chemicals and peptides** |  |  |
| DiR | UElandy | Cat#D4006 |
| DiD | Beyotime | Cat#C1039 |
| DiI | Beyotime | Cat#C1036 |
| DAPI staining solution | Beyotime | Cat#C1005 |
| LysoTracker deep red | Invitrogen | Cat#L12492 |
| Fixable viability dye 450 | Invitrogen | Cat#65-0863-14 |
| Brefeldin A | Sigma-Aldrich | Cat#B5936 |
| Montanide ISA 51 | SEPPIC | N/A |
| Squalene | Sigma-Aldrich | Cat#S3626 |
| Vitamin E-TPGS | Aladdin | Cat#T110277 |
| Polysorbate 80 | Sigma-Aldrich | Cat#59924 |
| α-tocopherol | Sigma-Aldrich | Cat#T3251 |
| Mannitol | Sigma-Aldrich | Cat#1.00419 |
| DOPC | AVT | Cat#C10519 |
| DSPE-MPEG2000 | AVT | Cat#C20128 |
| Tris | Solarbio | Cat#T8060 |
| SDS | Solarbio | Cat#S8010 |
| Tricine | Yuanye | Cat#S16030 |
| CSSSIINFEKL | Genscript | N/A |
| FITC-SIINFEKL | Sangon | N/A |
| CRAHYNIVTF | Genscript | N/A |
| Cholesterol-CpG | General Biol | N/A |
| Cholesterol-CpG-Cy5 | General Biol | N/A |
| CTAPDNLGYM | Genscript | N/A |
| CSSVDWENVSPELNSTDQ | Genscript | N/A |
| LCPGNKYEM | Genscript | N/A |
| **Critical commercial kits** |  |  |
| Murine IL-12 standard TMB ELISA kit | PeproTech | Cat#900-T97 |
| Mouse TNF alpha ELISA kit | Invitrogen | Cat#BMS607-3 |
| Chromogenic LAL endotoxin assay kit | Genscript | Cat#L00350 |
| Pierce silver stain kit | Thermo Scientific | Cat#24612 |
| Tris-Tricine-SDS-PAGE | Solarbio | Cat#P1320 |
| **Experimental models: cell lines and animals** |  |  |
| Cell line: B16F10 | China infrastructure of Cell Line Resources for Cell Ordering Service | N/A |
| Cell line: TC1 | Shanghai Xuanya Bio | N/A |
| Cell line: TC1-luc | Mingzhou Bio | N/A |
| Animal: C57BL/6 wild type | Vital River Laboretory Animal Technology Co., Ltd | N/A |
| Animal: ApoE knockout | GemPharmatech Co., Ltd | T001458 |
| Animal: LDLR knockout | Cyagen | C001392 |
| **Software** |  |  |
| Prism 8 | GraphPad | N/A |
| FlowJo | BD | N/A |
| ImageJ | NIH | N/A |
| IVIS lumina living Image | Caliper | N/A |
| NIS-elements | Nikon | N/A |
| N/A | Zeiss | N/A |
| **Other** |  |  |
| Leica CM3050 cryostat | Leica | N/A |
| Zeiss 780 confocal microscope | Zeiss | N/A |
| A1Rsi HD25 confocal microscope | Nikon | N/A |
| LSRFortessa | BD | N/A |
| HT 7800 TEM | HITACHI | N/A |
| Optima MAX-XP 342 | Beckman | N/A |
| Lumina III | PerkinElmer | N/A |
| 3DHISTECH | Pannoramic SCAN | N/A |
